# Intestinal Stem Cells Exhibit Conditional Circadian Clock Function

**DOI:** 10.1016/j.stemcr.2018.10.010

**Published:** 2018-11-13

**Authors:** Kathyani Parasram, Nathaniel Bernardon, Maha Hammoud, Hanna Chang, Li He, Norbert Perrimon, Phillip Karpowicz

**Affiliations:** 1 Department of Biological Sciences, University of Windsor, Windsor, ON N9B 3P4, Canada; 2 Department of Genetics, Harvard Medical School, Boston, MA 02115, USA

**Keywords:** circadian rhythms, intestinal stem cells, *Drosophila*

## Abstract

The circadian clock is a molecular pacemaker that produces 24-hr physiological cycles known as circadian rhythms. How the clock regulates stem cells is an emerging area of research with many outstanding questions. We tested clock function *in vivo* at the single cell resolution in the *Drosophila* intestine, a tissue that is exquisitely sensitive to environmental cues and has circadian rhythms in regeneration. Our results indicate that circadian clocks function in intestinal stem cells and enterocytes but are downregulated during enteroendocrine cell differentiation. *Drosophila* intestinal cells are principally synchronized by the photoperiod, but intestinal stem cell clocks are highly responsive to signaling pathways that comprise their niche, and we find that the Wnt and Hippo signaling pathways positively regulate stem cell circadian clock function. These data reveal that intestinal stem cell circadian rhythms are regulated by cellular signaling and provide insight as to how clocks may be altered during physiological changes such as regeneration and aging.

## Highlights

• Intestinal epithelial cells have circadian clock function but enteroendocrine cells do not
• Restricted feeding can entrain circadian clocks in the absence of photoperiod
• Circadian clock communication exists between intestinal stem cells and enterocytes
• Notch, Wnt, and Hippo signaling regulate stem cell clock function

## Graphical Abstract

**Figure:**
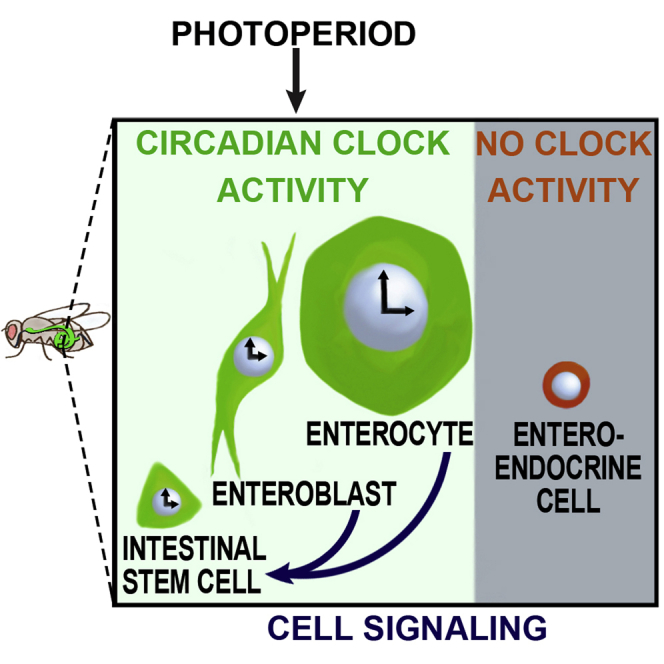

## Teaser

Parasram and colleagues demonstrate that circadian clock function is present throughout the intestinal epithelium, except in the enteroendocrine cell lineage. Cellular communication that influences intestinal stem cell clock function is regulated by pathways in the stem cell niche: Notch, Wnt, and Hippo signaling. Circadian clocks are functional in many physiological contexts, where they can be synchronized by photoperiod or the timing of food intake.

## Introduction

Circadian rhythms are 24-hr oscillations in animal physiology that are a product of the circadian clock. At its core, the circadian clock is composed of a conserved transcription/translation feedback system, whose activity throughout the cells of the body causes rhythms in their molecular functions (Hardin, 2011, Panda, 2016). Many animals contain a hierarchical circadian system, with clocks in brain neurons serving as a central pacemaker to transmit circadian timing to other peripheral tissues (Reppert and Weaver, 2002). In certain animals, such as insects and fish, most cells throughout the body can also directly transduce light/dark (photoperiod) cues to entrain their circadian clock timing (Hardin, 2011, Vatine et al., 2011). However, recent studies in *Drosophila* have shown that some elements of a hierarchical system are present and that signals propagated from the brain can drive rhythms in gene expression in distant organs (Xu et al., 2011). This suggests that inter-cellular signals that coordinate circadian timing throughout the animal body are conserved.

Transcriptomics has provided many insights into the genes that are regulated by the circadian clock, revealing that tissues have specific clock functions that can change under different physiological states (Tognini et al., 2017, Zhang et al., 2014). Most tissues are composed of a heterogeneous mixture of different cell types, and the role of the clock has been primarily studied at the tissue level. Fewer studies have analyzed specific cell populations within a single organ or tissue (Janich et al., 2011, Solanas et al., 2017). This is problematic, since readings would report signals from the average of all cells and obscure differences between different cell types or differences between cells of the same type. It is not clear whether all cells, including stem cells, in a single tissue contain circadian clocks, whether all cells of a specific cell type are homogeneous or heterogeneous in their clock functions, or whether changes occur under different physiological contexts. Although the imaging of cell cultures has provided information about clock function at the single-cell level (Nagoshi et al., 2004, Yeom et al., 2010), *in vitro* conditions contain a milieu of growth factors and cytokines that can affect circadian clock entrainment (Balsalobre et al., 2000). Hence, the synchrony and heterogeneity of circadian rhythms in tissue cells is not clear.

Another long-standing question is at what point the circadian clock arises during development (Agrawal et al., 2017, Brown, 2014, Umemura et al., 2017, Yagita et al., 2010). The clock is absent in mouse embryonic stem cells (Yagita et al., 2010) and only begins to function during embryonic differentiation (Umemura et al., 2017). In adult mice, circadian rhythms have been proposed to occur in certain populations of mouse hair follicle stem cells (Janich et al., 2011) and muscle stem cells (Solanas et al., 2017). *In vitro*, it was recently reported that mouse intestinal stem cells (ISCs) do not exhibit circadian rhythms and that clock function develops in differentiated cell types (Matsu-Ura et al., 2016). It is therefore not clear if tissue stem cells have circadian clock activity.

To answer these questions, we tested clock function *in vivo* at the single-cell resolution in the *Drosophila* intestine, a pseudo-stratified epithelium that contains a well-defined cell population. The *Drosophila* intestine contains a population of ISCs that, like those found in mammals, divide throughout life to produce all of the differentiated epithelial cells of the intestine (Biteau et al., 2011). Previously, we showed that the circadian clock regulates regeneration timing in the *Drosophila* intestine and that circadian gene dysfunction in stem cells is deleterious, suggesting that ISCs have clock activity that is important for their function (Karpowicz et al., 2013). Like mammals, the *Drosophila* intestine contains ISCs that divide to give rise to enteroblasts (EBs), which differentiate into either absorptive enterocytes (ECs) or nutrient-/pathogen-sensing enteroendocrine cells (EEs) that convey information about the intestinal environment to the body (Beebe et al., 2015, Park et al., 2016, Song et al., 2014). *Drosophila* ISCs are an undifferentiated population of cells in the intestinal epithelium, whose progeny terminally differentiate into tissue-specific cells. Because circadian rhythms are proposed to play a critical role in stem cell biology (Brown, 2014), we used this system to answer questions surrounding circadian clock activity in stem cells and their surrounding tissue cells.

Our data reveal that clocks are present in ISCs, EBs, and ECs, but not in EEs, showing that clock function does not necessarily correlate to cellular differentiation status. Circadian clocks in *Drosophila* intestinal cells are subject to signaling cues, including the timing of food intake. During intestinal stress, ISC clock function is dependent on surrounding cells, and the Notch (N), Wnt, and Hippo signaling pathways, important regulators of the ISC niche, also regulate circadian clock function in ISCs. These results shed light on how tissue stem cell clock rhythms are integrated with the surrounding tissue cells and how physiological changes during regeneration and aging can alter these rhythms.

## Results

### Circadian Clock Activity Is Heterogeneous in the Intestine

**Figure 1:**
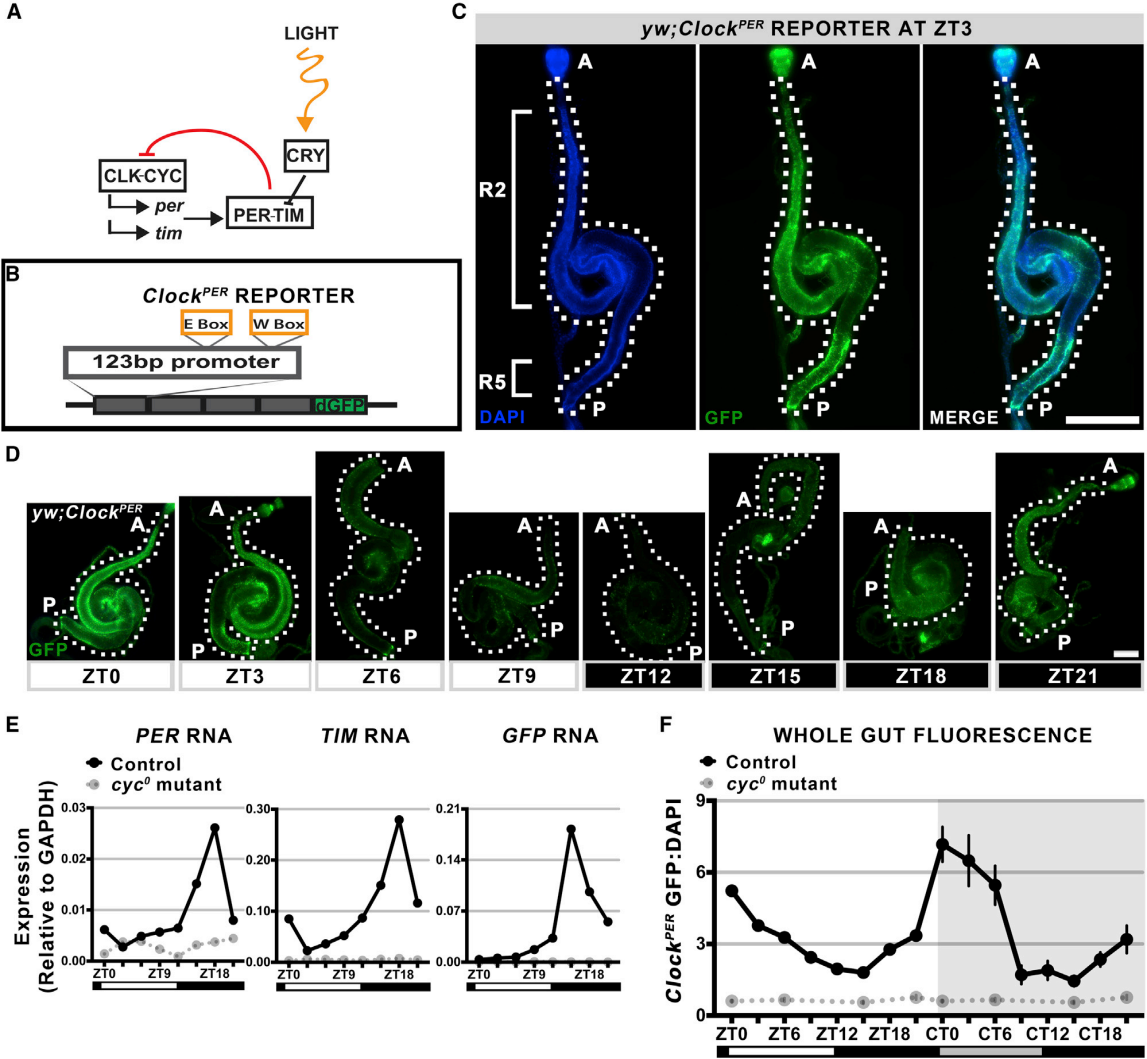
The *Clock*^*PER*^ Reporter Measures Intestinal Circadian Clock Activity (A) Schematic of the circadian clock in *Drosophila* showing that CLK/CYC promotes transcription, and PER/TIM repress this activity. Light acts on CRY degrading TIM to entrain the *Drosophila* clock to photoperiod. (B) Schematic of the *Clock*^*PER*^ reporter where 123bp *PER* promoter drives expression of destabilized GFP (dGFP) to report temporal changes in clock activity. (C) Representative image of a *Drosophila* intestine at ZT3 showing *Clock*^*PER*^ (GFP) in the anterior (R2) region and in the posterior (R5) region. DAPI counterstains nuclei. A, the anterior region; P, posterior. Scale bar represents 500 μm. (D) Representative images of *Clock*^*PER*^ (GFP) over a 24-hr timeline under LD photoperiod shows 24-hr changes in expression with a peak at ZT0. A, anterior region; P, posterior. Scale bar represents 500 μm. (E) RT-qPCR expression of entire *Clock*^*PER*^ intestine for *GFP*, *PER*, and *TIM* shows that these have similar expression phases, hence the *Clock*^*PER*^ reporter reports endogenous CLK/CYC transcriptional activity. Each data point represents a signal obtained from n = 10 intestines. Results of additional qPCR experiments are shown in Figure S1. (F) Graph of *Clock*^*PER*^ GFP signal normalized to DAPI under LD photoperiod, followed by 24 hr in DD for the entire intestine. Circadian rhythms of GFP are present, and the *cyc*^*0*^ mutant has no circadian transactivation and is thus negative at all times. Data presented as mean of n ≥ 10 intestines, error bars show ±SEM (two-way ANOVA F = 9.552, p < 0.0001). See also Figures S1 and S2.

The *Drosophila* circadian clock regulates gene expression and comprises the transactivators *CLK/CYC* and their targets and negative repressors *PER/TIM* (Figure 1A). To visualize clock activity in the *Drosophila* intestine, we constructed two clock reporters: (1) *Clock*^*PER*^ containing 123 bp of the PER promoter (Hao et al., 1997); (2) *Clock*^*TIM*^ containing 174 bp of the TIM promoter (McDonald et al., 2001), both arranged in a 4× tandem series upstream from a nuclear localization signal/superfolder destabilized GFP (Figures 1B and S2A). To quantify circadian transcription of this reporter in the intestine, we synchronized *Drosophila* carrying these reporters to 12-hr light/12-hr dark (LD) for 5 days, then tested gene expression in the intestine of controls versus clock-dead *cyc*^*01*^ null mutants by RT-qPCR. *GFP* RNA expression from both reporters was rhythmic, in phase with both *PER* and *TIM* (peak is zeitgeber time [ZT]15–18), and was *CYC* dependent (Figures 1E and S1). The expression of *PER* and *TIM* in the intestine is consistent with our previous results for clock gene transcription (Karpowicz et al., 2013). These data demonstrate that transcription of *GFP* from the reporter construct recapitulates endogenous CLK/CYC transcriptional activity in the intestine.

Intestines were examined visually: *Clock*^*PER*^ and *Clock*^*TIM*^ intestines both express reporter GFP, but it is strongest in the anterior regions and the posterior regions (Figure 1C). We collected n ≥ 10 individual intestines from synchronized *Drosophila*, imaged them using a fluorescent slide scanner, and quantified GFP signal from tiled whole-tissue scans containing z stacks of all of the cells present throughout the entire epithelium (see Experimental Procedures for details). Under LD conditions, both reporters showed rhythms in the whole intestine that were *CYC* dependent, consistent with their RNA expression (Figures 1D, 1F, and S2B), and the peak of *GFP* RNA expression preceded its signal by approximately 6 hr (ZT18 and ZT0, respectively). To confirm that these fluorescence rhythms were circadian in nature, *Drosophila* containing *Clock*^*PER*^ were shifted to complete dark photoperiod (DD), immediately following LD synchronization, and intestines were collected for an additional 24 hr. GFP rhythms persisted under these conditions in controls but not in *cyc*^*01*^ mutants, indicating that the *Clock*^*PER*^ reporter accurately reflects free-running *CYC*-dependent circadian clock activity in the *Drosophila* intestine (Figure 1F).

**Figure 2:**
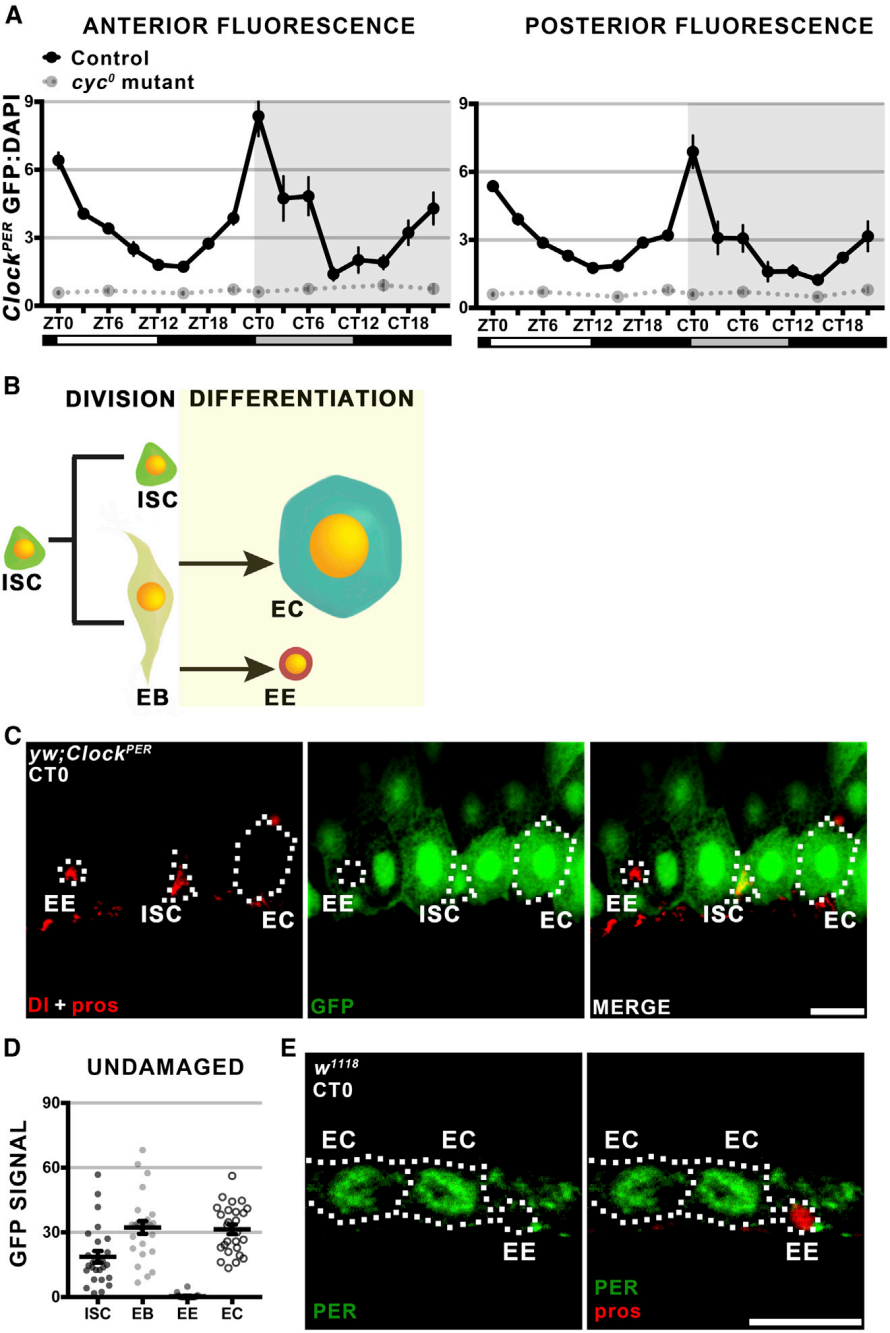
The C*lock*^*PER*^ Reporter Is Not Expressed in All Intestinal Epithelial Cells (A) Graphs of *Clock*^*PER*^ GFP signal normalized to DAPI under LD photoperiod followed by 24 hr in DD for anterior (left) and posterior (right). Both regions display similar circadian rhythms and the *cyc*^*0*^ mutant shows no circadian transactivation. Data presented as mean of n ≥ 10 intestines. Error bars show ±SEM. Control versus *cyc*^*0*^: anterior (two-way ANOVA F = 5.842, p < 0.0001), posterior (two-way ANOVA F = 10.7, p < 0.0001). (B) Schematic of the differentiation of Delta (Dl) positive ISCs, which self-renew and produce differentiated Dl-negative EBs, progenitor cells that differentiate into absorptive ECs or prospero (pros)-positive EE. (C) Representative confocal z stack showing *Clock*^*PER*^ GFP signal in the epithelium. Cells of interest are outlined: Dl+ marks ISCs, and pros+ marks EEs; ECs are the large polyploid cells. Scale bar represents 10 μm. (D) Quantification of GFP intensity from confocal sections shows clock activity is absent in EEs, but present in the other three epithelial cell types (one-way ANOVA F = 42.49, p < 0.0001). Data show n > 25 cells from each cell type, error bars show ±SEM. (E) Confocal section showing nuclear PER antibody staining in ECs but not EEs. Cells of interest are outlined: pros+ marks EEs, ECs are the large polyploid cells. Scale bar represents 10 μm. See also Figure S3.

The *Drosophila* intestine is subdivided into >10 different anterior to posterior sub-regions based on cell composition, morphology, and gene expression (Buchon et al., 2013). Since GFP signal throughout the intestine is heterogeneous (Figure 1C), we asked whether clock activity varied throughout this tissue. To test this, the R2 (anterior) versus R5 (posterior) regions were quantified separately from the same tissue samples. No differences in circadian clock phase were noted between the regions (Figure 2A); both essentially followed the same timed rhythms as the whole tissue (Figure 1F). This suggests that, despite dissimilar physiological functions, circadian clocks in the anterior to posterior intestine of *Drosophila* are synchronously timed.

Genome-wide analysis has previously indicated that ISCs, EBs, EEs, and ECs in the *Drosophila* intestine (Figure 2B) express clock genes at some level (Figure S3A) (Dutta et al., 2015). However, circadian clock function cannot be assumed based on these data, because circadian gene expression must be tested at a specific time of day to determine rhythms in transcript expression. We tested circadian activity in all four cell types at CT0 (the peak of *Clock*^*PER*^ signal), when intestinal epithelial cells are GFP+, indicative of CLK/CYC transcriptional activity (Figure 2C). We quantified the fluorescence signal of single confocal sections through the middle of each cell, revealing that all ISCs, EBs, and ECs express the *Clock*^*PER*^ reporter signal but EEs do not exhibit reporter activity (Figure 2D). The unexpected finding that EEs do not have clock activity was further tested using the *Clock*^*TIM*^ reporter, this time inserted on a different chromosome to avoid possible EE-specific *PER* promoter silencing and/or chromatin silencing effects. EEs do not express GFP from the *Clock*^*TIM*^ reporter either (Figures S3C–S3E). We further asked if EEs might have clock activity out of phase with the other cell types, by testing them at different time points than CT0, but found no signal at any time (Figure S3F). Finally, PER protein was tested by antibody staining, which we have previously shown to be nuclear in ISC and EC cells (Karpowicz et al., 2013). The majority of EEs examined have very low PER signal, especially in the nucleus, compared with EC cells where PER nuclear staining is strong (Figures 2E and S3G). Overall these data suggest that clock activity is present in undifferentiated stem cells and EBs, as well as differentiated ECs, but is turned off during EE differentiation. Clock function does not correlate with increased differentiation in this tissue, and *Drosophila* ISCs exhibit clock activity consistent with previous studies of skin and muscle stem cells in mammals (Janich et al., 2011, Solanas et al., 2017).

### Feeding Can Regulate the *Drosophila* Intestinal Clock

**Figure 3:**
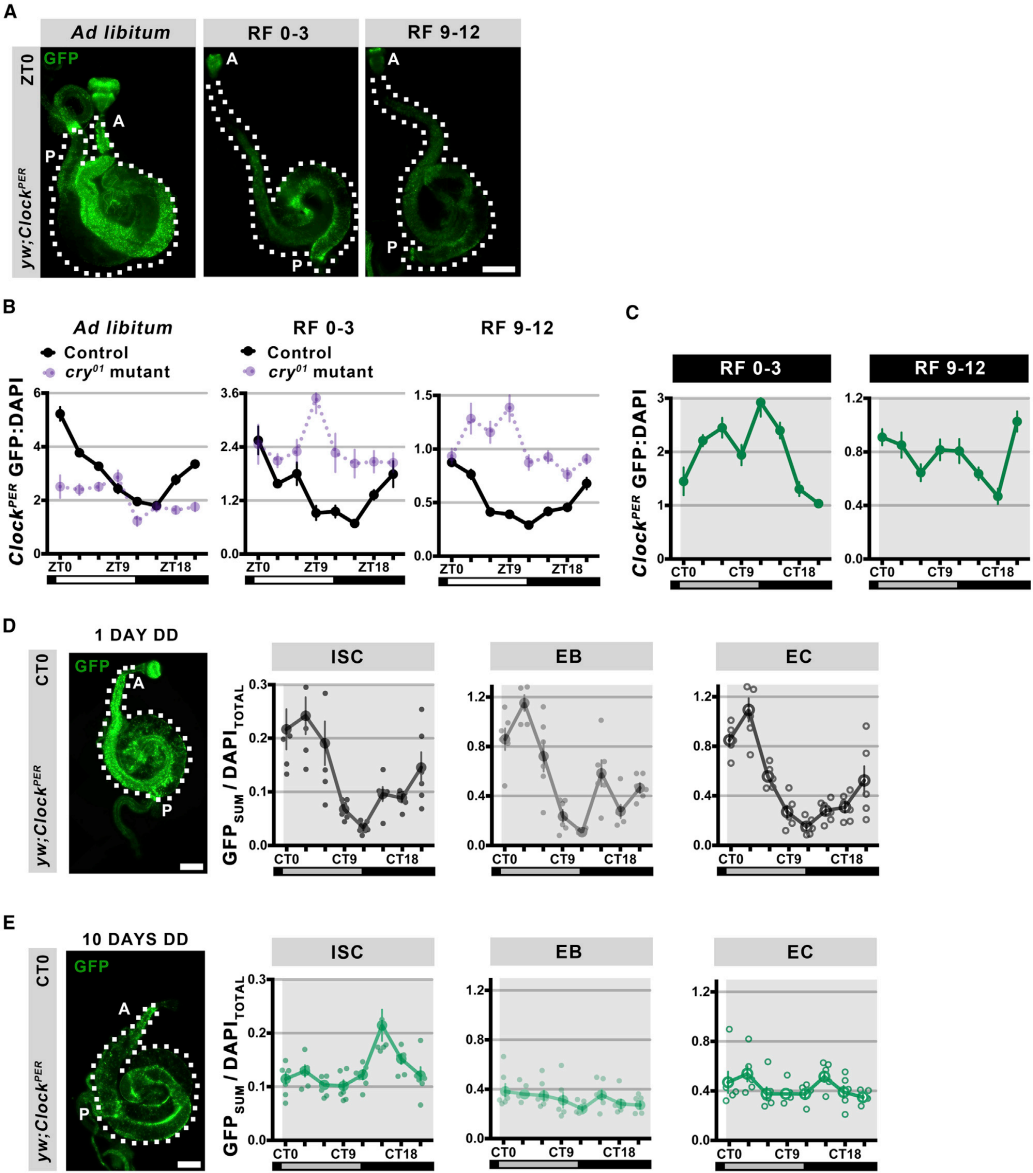
Feeding Time Can Entrain the *Drosophila* Intestinal Clock (A) Representative images of *Clock*^*PER*^ intestines from flies fed *ad libitum* or restricted to ZT0-3 or ZT9-12. GFP+ signal is lower under restricted feeding. A, the anterior region; P, posterior. Scale bar represents 250 μm. (B) Graphs of *Clock*^*PER*^ GFP:DAPI signal in the whole intestine under LD photoperiod under *ad libitum* or restricted feeding. Control data (left graph) are the same as used in Figure 1F. *Ad libitum*-fed *CRY* mutants (*cry*^*01*^) show higher GFP levels during the daytime (ZT0-9) than night (ZT12-21), and are significantly different than controls (two-way ANOVA F = 13.15, p < 0.0001). Under restricted feeding, control intestines follow similar rhythms (albeit with different amplitude), with the same peaks and troughs irrespective of feeding regimen, suggesting that photoperiod is the key entrainment factor in the intestine. *CRY* mutants show distinct rhythms in these different regimens. *cry*^*01*^ are significant in RF0-3 (one-way ANOVA F = 2.417, p = 0.0270) and RF9-12 (one-way ANOVA F = 6.083, p < 0.0001); control versus *cry*^*01*^ is significant: RF0-3 (two-way ANOVA F = 5.092, p < 0.0001), RF9-12 (two-way ANOVA F = 9.63, p < 0.0001). Data presented as mean of n ≥ 10 intestines, error bars show ±SEM. (C) Graphs of *Clock*^*PER*^ GFP:DAPI signal in wild-type flies with 5 days of restricted feeding at CT0-3 versus CT9-12 (DD conditions). Alterations in clock reporter activity suggest the timing of feeding affects clock function in these otherwise free-running conditions. Data presented as mean of n ≥ 10 intestines, error bars show ±SEM. One-way ANOVA: control 0–3 (F = 12.48, p < 0.0001); control 9–12 (F = 2.161, p = 0.0467). (D) Representative image of a *Clock*^*PER*^ GFP+ *Drosophila* intestine at CT0, at 1 day DD. DAPI counterstains nuclei. A, the anterior region; P, posterior. Scale bar represents 250 μm. Graphs show quantification of cell-specific rhythms in the posterior (R5) region with EC, EB, and ISC signals analyzed separately. One day after LD photoperiod, all clock active intestinal cells exhibit synchronous circadian rhythms. GFP_SUM_ is fluorescence from n = 5 cells of each cell type (ISC, EB, or EC) normalized to the DAPI coming from all cells quantified (n = 15 total). Data are the mean of n = 6 intestines, error bars show ±SEM. One-way ANOVA: ISC (F = 8.485, p < 0.0001), EB (F = 21.36, p < 0.0001), EC (F = 26.37, p < 0.0001). (E) The same analysis carried out 10 days after DD. Following a long period of free-running conditions, intestinal cells are no longer synchronous and the average rhythm for each cell type is altered. One-way ANOVA: ISC (F = 6.283, p < 0.0001), EB (F = 1.332, p = 0.2608), EC (F = 2.083, p = 0.0679).

In mammals, signals from the brain synchronize circadian timing throughout the body (Reppert and Weaver, 2002), but restricting feeding time can synchronize peripheral tissues such as the liver directly (Damiola et al., 2000). Unlike mammals, *Drosophila* cells are themselves directly responsive to photoperiod (Hardin, 2011), yet recent studies have shown that feeding time can entrain the fat body (the insect equivalent of the liver) directly (Xu et al., 2011). To test whether the intestine is entrainable by feeding time, we used the *Clock*^*PER*^ reporter in the *cry*^*01*^ mutant background. In *Drosophila*, *CRY* transduces photoperiod entrainment of the clock by targeting *TIM* for degradation (Figure 1A) (Emery et al., 1998), and CRY+ cells are present in the *Drosophila* intestine (Agrawal et al., 2017). Hence we predicted that the *cry*^*01*^ mutant would reveal circadian synchronizing cues that might be normally obscured by the photoperiod. Under LD *ad libitum* feeding conditions, control intestines show a rhythm that peaks at ZT0; *CRY* mutants do not show this rhythm but instead have slightly elevated CLK/CYC activity during the light phase (ZT0-9) (Figures 3A and 3B), perhaps reflecting the time when flies are most active and feeding. Restricting feeding to ZT0–3, the peak of maximal food consumption (Xu et al., 2008), or ZT9–12 in LD conditions, does not affect wild-type intestine rhythms. These maintain the same phase circadian rhythms under restricted feeding as *ad libitum* feeding, although with a lower amplitude, likely due to decreased overall food consumption (Figures 3A and 3B). This indicates that photoperiod is a dominant entraining factor in the *Drosophila* intestine, consistent with the dominant effect of photoperiod in regulating activity rhythms (Oishi et al., 2004). However, *cry*^*01*^ mutant intestines exhibit peaks in CLK/CYC activity, with a peak at ZT9 if fed at ZT0–3, and a peak at ZT3–9 if fed at ZT9–12 (Figures 3A and 3B). Although feeding time did not produce a consistent CLK/CYC activity time (i.e., the reporter peaks 6 hr after ZT0–3 feeding but peaks 15 hr after ZT9–12 feeding), these results suggest that food intake can regulate CLK/CYC transactivation when photoperiod cues are not transduced through *CRY*.

We further tested the ability of restricted feeding to entrain intestinal clocks by maintaining wild-type *Clock*^*PER*^ *Drosophila* for 5 days in the absence of photoperiod under a restricted feeding regimen. In peripheral tissues, it has been noted that circadian clock activity dampens in individuals maintained under free-running conditions for >2 days (Ivanchenko et al., 2001, Stanewsky et al., 1997), hence we predicted that two different restricted feedings would entrain two different *Clock*^*PER*^ rhythms. When fed at CT0–3, reporter activity has a double-peak distribution with a maximum at CT12 (Figure 3D). Feeding at CT9–12 also produces a double-peak distribution but with a maximum at CT21 (Figure 3D). Thus, in both cases, feeding preceded a peak of CLK/CYC activity by 9 hr, suggesting that food intake can regulate circadian clock function in the wild-type intestine when photoperiod is absent. We next tested the importance of photoperiod on synchronizing intestinal cell clocks, by comparing *Drosophila* exposed to different duration of DD free-running conditions. One day following shift from LD to DD, intestinal cells maintain synchronous circadian rhythms, with ISCs, EBs, and ECs in phase (Figure 3D). However, at 10 days DD free running, intestinal cells lose their rhythmicity and ISCs, EBs, and ECs are either arrhythmic or out of phase (Figure 3E). These data suggest that intestinal cells drift out of synchrony if the individual loses long-term photoperiod cues, and behavior and physiological processes lose 24-hr rhythms. Taken together, our data show that photoperiod is a dominant synchronization cue for *Drosophila* intestinal clocks, but, in its absence, intestinal cell clocks can be regulated by timed food intake. This bears resemblance to the *Drosophila* fat body (Xu et al., 2011) and suggests that cell signaling processes may be able to modulate clock activity in the *Drosophila* intestine.

### Intestinal Stem Cell Clock Activity Is Non-autonomous

**Figure 4:**
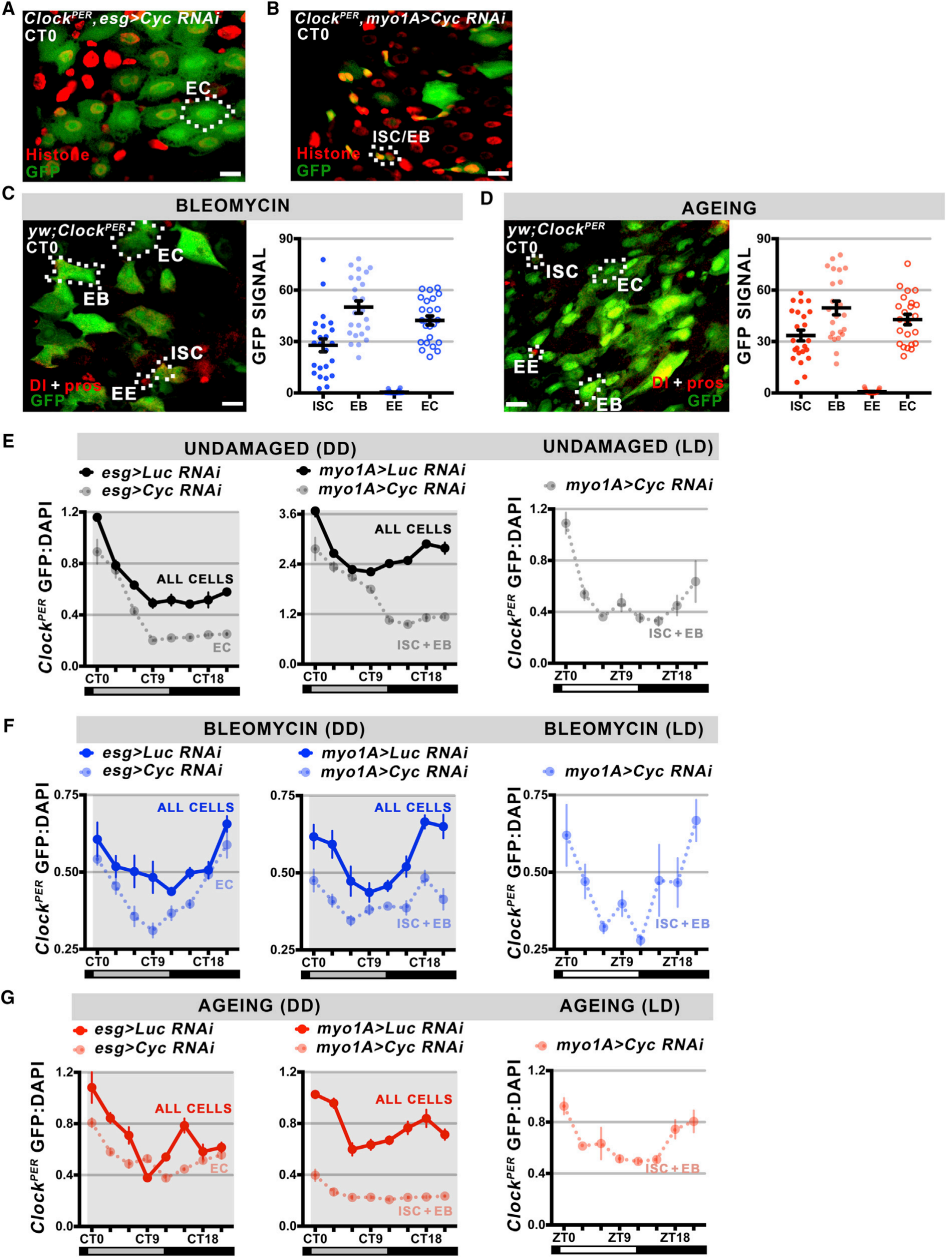
Intestinal Stem Cell Clock Activity Is Regulated by Non-Cell-Autonomous Factors (A) Confocal z stack of *Clock*^*PER*^ reporter in ISC/EB-specific *esg>CYC* knockdown. ECs, ISCs, and EBs are indicated, the smaller GFP+ cells are parts of differentiated ECs (that do not express *esg*) just outside the confocal stack. (B) Confocal z stack of *Clock*^*PER*^ reporter in EC-specific *myo1A>CYC* knockdown. ISC/EBs are indicated, the larger GFP+ cells with processes are most likely EBs differentiating into ECs but that do not yet express *myo1A*. Histone counterstains all nuclei present, scale bar represents 10 μm. (C) Analysis of *Clock*^*PER*^ GFP signal from confocal sections for bleomycin-treated flies. Scale bar represents 10 μm. ISCs, EBs, and ECs are GFP+ while EEs are GFP−. Data presented for >25 cells in each group, error bars show ±SEM (one-way ANOVA F = 56.34, p < 0.0001). (D) Analysis of *Clock*^*PER*^ GFP signal from confocal sections for aged flies. The distribution of cells during aging stress closely mirrors that observed during bleomycin-induced stress. Data presented for >25 cells in each group, error bars show ±SEM (one-way ANOVA F = 53.91, p < 0.0001). (E) Left graph shows *Clock*^*PER*^ GFP:DAPI signal for control (*Luc RNAi*) and *Cyc RNAi* knockdown in ISCs and EBs (*esg*) under DD conditions. ECs are not affected by the knockdown of *CYC* in the surrounding precursors (left) and express similar clock activity (see Figure 3D). *esg>Luc RNAi* versus *Cyc RNAi* (two-way ANOVA F = 2.701, p = 0.0115). Center graph shows *Clock*^*PER*^ GFP:DAPI signal for control (*Luc RNAi*) and *Cyc RNAi* knockdown in ECs (*myo1A*) under DD conditions. When *Cyc* is knocked down in ECs (right), *Clock*^*PER*^ GFP rhythms in control are higher and phase advanced compared with *Cyc RNAi* knockdown in ECs, and compared with their normal rhythms (see Figure 3D). *myo1A>Luc RNAi* versus *Cyc RNAi* (two-way ANOVA F = 20.15, p < 0.0001). Right graph shows *Clock*^*PER*^ GFP:DAPI signal for myo1A > *Cyc RNAi* under normal 12:12 LD photoperiod. Under LD, ISC/EB rhythm (one-way ANOVA F = 9.577, p < 0.0001) is normal (compare with Figure 3D). (F) Analysis of *Clock*^*PER*^ GFP:DAPI signal for the same genotypes as above, in bleomycin-treated flies. Loss of clock activity in ISCs and EBs does not alter rhythms in ECs (left); however, loss of clock in ECs reduces reporter rhythm in ISCs and EBs compared with undamaged conditions (center). *CYC RNAi* undamaged versus *Cyc RNAi* bleomycin-treated (two-way ANOVA F = 18.05, p < 0.0001). Under LD conditions, ISC/EB rhythm is normal (one-way ANOVA F = 3.72, p = 0.0032). (G) Analysis of *Clock*^*PER*^ GFP:DAPI signal in aged flies. During aging, both controls (*Luc RNAi*) have persistent rhythms when clock activity is lost in ISC/EBs. Loss of clock activity in ECs causes the ISC/EB clock to have very low amplitude (one-way ANOVA F = 10.45, p < 0.0001), which is significantly altered compared with undamaged conditions: *CYC RNAi* undamaged versus *Cyc RNAi* aged (two-way ANOVA F = 16.56, p < 0.0001). Under LD conditions, the ISC/EB rhythm is again rescued (one-way ANOVA F = 6.447, p < 0.0001). Data presented as mean (n ≥ 8 guts), error bars show ±SEM.

Previously, we reported that clock function is required in non-dividing differentiated ECs to generate circadian rhythms in stem cell proliferation (Karpowicz et al., 2013). This raised the question of whether a circadian communication system exists between the cells in the epithelium or whether cell-specific clocks function cell autonomously. To test this, we disrupted clock function in specific cell types: the *esg-Gal4* driver to disrupt *CYC* in undifferentiated precursors (ISCs + EBs) and the *Myo1A-Gal4* driver to disrupt *CYC* in differentiated ECs. Because clock activity in the intestinal epithelium is restricted to ISCs, EBs, and ECs (Figures 2C and 2D), these experiments test whether circadian rhythms are present in specific epithelial cell types when those surrounding them are absent. We entrained *Clock*^*PER*^ flies carrying cell-specific *CYC* disruption constructs to LD, then released them into free-running DD conditions and analyzed tissue over 24 hr (Figures 4A and 4E). Loss of clock function in undifferentiated precursors (ISCs + EBs) did not disrupt circadian rhythms in differentiated ECs whose circadian timing persisted (Figure 4E), suggesting that EC clocks are not dependent on clocks in undifferentiated precursors. In contrast, loss of clock function in ECs alters the circadian rhythms of undifferentiated precursors (Figure 4E). This was apparent for two reasons. First, the number of precursors positive for clock activity after EC disruption was lower than the total number of precursors present in the intestine (Figure S3B), and which we previously found to be clock reporter active (Figure 2D). Second, the remaining clock-positive precursors continue to display a peak of clock activity at CT0, but CLK/CYC activity is prolonged (up to CT9) before returning to its baseline levels (Figure 4B; compare with Figure 3D). This suggests that PER/TIM repression is slightly delayed in the few remaining clock-live precursor cells when EC clocks are lost. We then tested whether restoring photoperiod could rescue clock function in ISC/EBs when the circadian clock was disrupted in ECs. Under LD photoperiod, *esg>CYC RNAi* ISC/EBs have normal clock function (Figure 4E), suggesting that indeed this is the case.

ISCs are highly sensitive to signals present in their niche that modulate tissue regeneration (Biteau et al., 2011). We challenged the *Clock*^*PER*^ intestine to chemical damage (bleomycin) that results in the upregulation of signaling pathways involved in the rapid division of stem cells (Amcheslavsky et al., 2009). We first tested whether clock activity was altered in the different intestinal cell types under these conditions or whether they maintained their normal clock function. At CT0, ISCs, EBs, and ECs continued to show CLK/CYC transcriptional activity, while EEs remain clock dead (Figure 4C). Similar to undamaged conditions, cell-specific knockdown of *CYC* in ECs produces a similar rhythm as in controls (Figure 4F), again suggesting that differentiated cells are resistant to the clock status of their undifferentiated neighbors. However, when CYC is disrupted in differentiated ECs, undifferentiated precursors have a phase-shifted and lower clock rhythm than the one present under undamaged conditions (Figure 4F; compare with 3D). However, photoperiod is able to rescue clock function in chemically stressed ISC/EBs (Figure 4F), suggesting direct light is the dominant factor in ISC/EB clock function.

Several recent studies have examined clock function during aging, where alterations in circadian clock target genes are thought to contribute to changes in gene expression and tissue physiology (Kuintzle et al., 2017, Solanas et al., 2017). We repeated our cell-specific knockdown of *CYC* in *Drosophila* aged >35 days, a time when stem cell dysfunction and stress signaling pathways become elevated (Biteau et al., 2008, Biteau et al., 2011). The *Clock*^*PER*^ reporter in aged cells showed very similar profiles to those observed under bleomycin-induced stress: ISCs, EBs, and ECs have CLK/CYC activity, whereas EEs are GFP negative (Figure 4D). Similar to chemical stress, the loss of CYC in ISCs/EBs had little effect on the circadian rhythms exhibited by ECs (Figure 4G); however, the loss of CYC in ECs caused these precursors to exhibit very low rhythmicity (Figure 4G), especially compared with young ISCs (Figure 3D) and undamaged conditions (Figure 4E). Similar to acute stress, photoperiod rescues clock function in ISC/EBs even when the surrounding ECs are clock negative (Figure 4G). These results again show that aged ISCs circadian clocks are responsive to clock activity in the surrounding differentiated ECs when photoperiod is absent. Taken together, these data support the existence of backup unidirectional circadian clock synchronization from differentiated ECs to undifferentiated precursors (ISCs and EBs) that is particularly sensitive to acute environmental or age-related stresses.

### Stem Cell Signaling Regulates Circadian Clock Activity

*Drosophila* stem cells are highly responsive to homeostatic cellular signaling pathways that mediate crosstalk between them and the surrounding tissue (Biteau et al., 2011). N signaling occurring between the ISCs and EBs regulate commitment to the EC lineage (Ohlstein and Spradling, 2007), whereas stress activates both the Hippo and Wnt signaling pathways in stem cells to initiate compensatory proliferation during regeneration (Cordero et al., 2012, Karpowicz et al., 2010, Shaw et al., 2010). To test whether these signaling pathways regulate stem cell circadian clock activity, we activated them using the *Gal4-UAS* system in a temperature-restricted fashion using the *Gal80*^*TS*^ repressor. The *esg-Gal4* driver was used to drive expression in undifferentiated precursors (ISCs + EBs), and flies bearing constructs were raised in restrictive 18°C temperatures (to silence the *Gal4-UAS* system) then shifted to permissive 29°C to activate genes of interest in the adult intestine.

**Figure 5:**
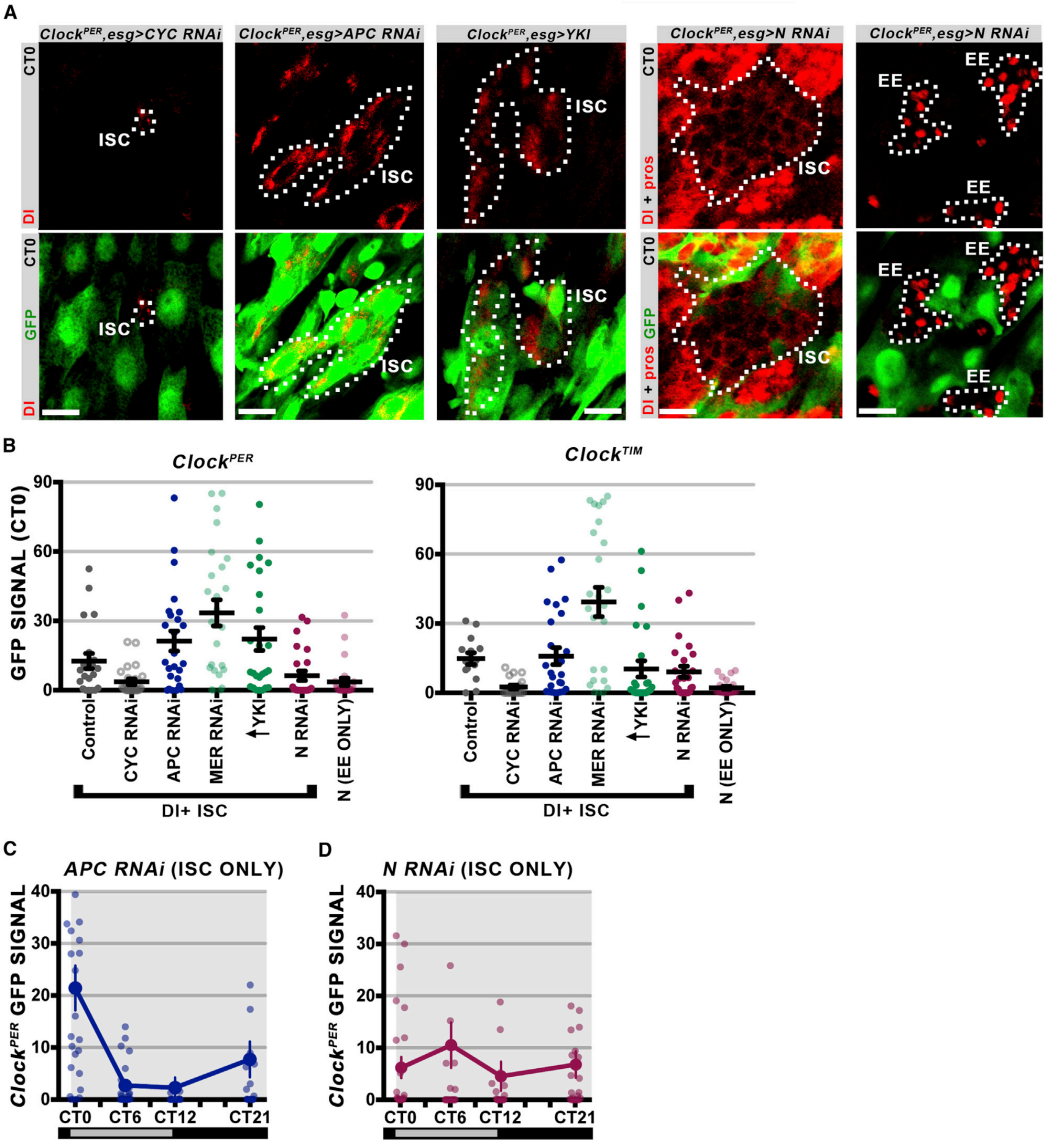
Stem Cell Signaling Pathways Affect ISC Circadian Clock Activity (A) Representative confocal z stacks of *Clock*^*PER*^ reporter for ISC/EB-specific pathway disruption at CT0. Dl+ and Pros+ are used to mark the ISC versus EE tumors, respectively. ISCs expressing *esg* in these tumors are outlined, showing the abnormal Dl+ cells caused by signaling pathway activation. CYC RNAi and N RNAi reduce GFP+, while APC RNAi and Yki overexpression increase it. Scale bar represents 10 μm. (B) Analysis of *Clock*^*PER*^ and *Clock*^*TIM*^ GFP signals in single Dl+ cells from confocal sections at CT0. Activation of the Wnt or the Hippo pathway raises clock activity, and loss of the N pathway lowers clock activity. Data presented for n ≥ 15 cells in each group, error bars show ±SEM. One-way ANOVA for *Clock*^*PER*^ (F = 9.624, p < 0.0001); *Clock*^*TIM*^ (F = 12.99, p < 0.0001). (C) Twenty-four-hour analysis of *Clock*^*PER*^ from *esg>APC RNAi*. Elevated Wnt pathway does not alter the phase of normal circadian clock activity in ISCs (compare with Figure 3D). Data presented for n = 25 cells in each time point, error bars show ±SEM (one-way ANOVA F = 9.153, p < 0.0001). (D) The same analysis on *esg>N RNAi*. In this case, clock activity is not only lowered but becomes arrhythmic. Data presented for n = 25 cells in each time point, error bars show ±SEM (one-way ANOVA F = 0.6754, p = 0.5692).

We first targeted the Wnt pathway repressor *APC*, whose loss leads to uncontrolled hyperplasia by promoting the division of stem cells (Lee et al., 2009). Loss of *APC* increased *Clock*^*PER*^, and to a lesser extent *Clock*^*TIM*^ reporter activity (Figures 5A and 5B). Similarly, both positive (*Yki* overexpression) and negative (*Mer RNAi*) components of the Hippo pathway increased *Clock*^*PER*^, and to a lesser extent *Clock*^*TIM*^, reporter activity (Figures 5A and 5B). This suggests that CLK/CYC activity is increased in ISCs when they are actively proliferating due to active Wnt or Hippo signaling. We further tested the circadian nature of these phenotypes by testing Dl+ ISCs with activated Wnt pathway over 24 hr; ISCs that lose APC function display normal circadian rhythm periodicity (Figure 5C; compare with Figure 3D).

Loss of the N pathway in stem cell precursors leads to the formation of undifferentiated tumors, consistent with previous reports (Ohlstein and Spradling, 2007). Two types of tumors were observed: those composed of EEs (N signaling is low) or stem cells (N signaling is absent) (Figure 5A). ISC tumors show a heterogeneous mixture of reporter GFP+ and GFP− cells, while EE tumors showed lower *Clock*^*PER*^ and *Clock*^*TIM*^ reporter expression than controls, at similar levels to cells expressing CYC RNAi (Figures 5A and 5B). This suggests that the low-GFP+ ISCs are those committing to the EE lineage observed in normal (Figure 2D), damaged (Figure 4C), and aging (Figure 4D) conditions, while the high-GFP+ ISCs are those committing to the EC lineage. Disruption of N signaling in ISCs/EBs using the *esg* driver renders ISCs completely arrhythmic over 24 hr (Figure 5D; compare with Figure 3D). These data suggest that ISC circadian rhythms require some of the cellular signaling pathways that compose the stem cell niche (N signaling).

## Discussion

### Stem Cell Circadian Rhythms Are Regulated by Intestinal Signaling

The circadian clock has emerged as a central regulator of physiological processes and the coordination of those processes throughout the body (Panda, 2016, Xu et al., 2011). To explore questions surrounding the cell-specific nature of circadian rhythms, we tested circadian clock function in the intestinal epithelium during different physiological states. Previous analyses of circadian transcription in the intestine, including those arising from our own studies, have reported gene expression from the average of a heterogeneous mixture of intestinal epithelial cell types (Hoogerwerf et al., 2007, Hoogerwerf et al., 2008, Karpowicz et al., 2013, Sladek et al., 2007, Stokes et al., 2017). However, global analysis of circadian clock transcriptional function is limited as it reports the average of transcripts abundance throughout this tissue. Here, we show that clock activity occurs in only a subset of cells in the intestinal epithelium, which include ISCs, and that stem cell circadian clock activity can be modulated depending on their response to homeostatic signaling.

ISCs are surrounded by differentiated ECs, which compose the bulk of cells in the epithelium. When the circadian clock is specifically disrupted in ECs, the circadian core pacemaker in ISCs (driven by CLK/CYC transactivation) is reduced (Figures 4E–4G). Our results thus highlight that circadian clock communication exists between ECs and ISCs in the intestinal epithelium, with differentiated cells positively regulating the clocks of neighboring stem cells, particularly during conditions of stress. It is also likely that circadian signaling occurs between ISCs and EBs in this tissue, because disruption of the N pathway in ISC/EBs using the *esg* driver results in loss of rhythmicity in ISCs, which do not require N signaling. This ability to synchronize circadian timing between different intestinal cells bears some similarity to neuronal clocks of the brain, whereby clock-harboring cells communicate with one another to achieve a unified circadian output (Liu et al., 2007, Sabado et al., 2017). Although intestinal cells are clearly not so interdependent as neurons, a large repertoire of signaling processes are known to occur between intestinal cells (Biteau et al., 2011). It is important to note that over long-term, free-running conditions, these signaling pathways cannot maintain synchrony among intestinal cells (Figures 3D and 3E) and that photoperiod is a dominant synchronizer of circadian clocks (Figures 3B and 4E–4G). The physiological relevance of ECs to ISC circadian communication is not clear; they may play a minor role in *Drosophila* where cells are directly responsive to light and may simply fine-tune circadian timing between cells of this tissue. It will be important to extend these findings to systems where cells do not detect light (i.e., mammals) but rely exclusively on cellular communication to set clock function.

Stem cells have been noted to have heterogeneous clock activity (Janich et al., 2011), suggesting that circadian rhythm generation is dynamic in these undifferentiated cells. Indeed, our data shed light on how circadian communication is controlled by signaling: the Wnt and Hippo pathways, which are activated during regeneration, can boost CLK/CYC activity in ISCs (Figures 5A–5D). These and perhaps other pathways would link the cells of the intestine to achieve a unified circadian output during critical times of need. Indeed, we have reported that circadian rhythms in proliferation occur only during regeneration but not during normal tissue renewal in both *Drosophila* and mice (Karpowicz et al., 2013, Stokes et al., 2017). We thus propose that, during regeneration and stress, signaling pathways from the stem cell niche cause circadian rhythms to increase in amplitude in stem cells in order to synchronize the circadian output of these cells with the timing of organism physiology. Our data support that clock activity can be modulated dynamically within the same stem cell depending on environmental context. This suggests that, when tissues proliferate with 24-hr rhythms, this is due to both circadian rhythms occurring in the proliferating cells as well as those occurring in the surrounding cells that send signals to activate proliferation (Karpowicz et al., 2013).

### Enteroendocrine Cells Do Not Exhibit Circadian Clock Activity

EEs do not exhibit reporter expression in either the *PER* or *TIM* reporter constructs examined, and do not exhibit nuclear PER localization by antibody, leading us to conclude that EEs do not have circadian clock function (Figures 2C–2E and S3C–S3G). A recent study has revealed a population of EE precursor cells that respond directly to mechanical stimulation through the receptor, *Piezo* (He et al., 2018). The presence of GFP variability in ISCs and EBs may be due to GFP-low EE precursors that lose clock activity as they differentiate. This notion is consistent with our results indicating that in EE tumors arising from perturbation of the N pathway, EE precursor cells exhibit lower clock reporter expression. This may also explain why Dutta et al. (2015) previously found some clock genes to be expressed in EEs; these genes persist in the EE precursors before they fully differentiate. We thus propose that the circadian clock persists in intestinal cells committed to an EC fate and is extinguished in cells committing to the EE lineage. As the circadian clock has been proposed to regulate both metabolic (Panda, 2016) and immune functions (Man et al., 2016), it is somewhat surprising that EEs, which in part regulate these processes, would be clock deficient. Although we found neither clock reporter nor protein expression present, it is possible that EEs express low levels of *PER* and *TIM*, unlike the other cells of the intestine, and require additional regulatory sequences in addition to the CYC/CLK binding sites found in the *PER* and *TIM* promoters used in our study. Future work will further determine how and why EEs do not exhibit clock activity in *Drosophila*, whether EEs can turn on circadian clock activity under certain conditions, and whether these findings extend to other animals.

### Feeding Can Entrain *Drosophila* Intestinal Clocks

Like the circadian clock of the *Drosophila* fat body and the mouse liver (Damiola et al., 2000, Xu et al., 2011), clocks in intestinal cells can be regulated by the timing of food uptake. Specifically, restricting the time of feeding can alter CLK/CYC activity in both *CRY* mutants that are insensitive to photoperiod and in wild-type intestines in the absence of photoperiod. Hence, food intake may serve as a secondary entrainment factor in a tissue subject to circadian behavioral rhythms, which include the time of feeding. Because *Drosophila* cell clocks can directly respond to light, and we find that light-driven entrainment appears to predominate, we propose that food entrainment is a second synchronizing cue regulated by signaling processes that occur in digestive tract tissues. Photoperiod remains the dominant entrainment cue, consistent with findings previously documented in *Drosophila* behavior and metabolism studies (Oishi et al., 2004, Xu et al., 2011).

### Do Circadian Rhythms Occur during Pathological Conditions?

These data also provide insight into how normal circadian activity can be disrupted in pathological contexts, such as the process of tumorigenesis where the role of the circadian clock has been controversial (Sancar et al., 2015). It is likely that in different cancers, which may involve the activation or inactivation of different cellular signaling processes in cancer stem cells, circadian clock function may be either present or absent.

It was recently shown that mouse ISCs do not exhibit robust circadian activity *in vitro* (Matsu-Ura et al., 2016), similar to embryonic stem cells (Yagita et al., 2010), which have been shown to develop circadian rhythmicity as they differentiate (Umemura et al., 2017), yet in hair follicle stem cells and muscle stem cells, circadian clock activity has been observed (Janich et al., 2011, Solanas et al., 2017). This raises the question of when clock activity, which is absent in a pluripotent state, emerges as cells undergo differentiation. Our results support that cellular differentiation increases clock activity in the case of ECs, whose precursors are clock active during differentiation. However, the population of ISCs that spawn these is also clock active, and differentiation can conversely lead to termination of clock activity in the case of EE precursors. Our results shed light on why the stem cell population is heterogeneous for clock activity and why the overall readouts of such a population exhibit lower circadian rhythms than differentiated cells (Matsu-Ura et al., 2016). Circadian clock activity is defined in specific cell lineages rather than cell differentiation status and can be modulated under certain conditions. The surrounding differentiated cells signal to ISCs, and the presence or absence of these signals regulates clock function in stem cells. Our *in vivo* data also raise an important caveat to *in vitro* studies of stem cell biology: cellular signals, often present *in vitro* at non-physiological levels, may artificially perturb stem cell clock function. Stem cell clock function examined *in vivo*, in particular experiments involving live imaging of clock activity, would resolve these issues. The reporters generated in this study may be suitable for this purpose.

## Experimental Procedures

Flies were housed at 25°C under an LD cycle on standard media, unless otherwise noted. At each time point ∼10 intestines from female flies <14 days were dissected in PBS (Fisher) and fixed in 4% paraformaldehyde (Electron Microscopy Sciences) in PBS and then counterstained with DAPI (Thermo Fisher Scientific, 1:5,000) in PBS-T (PBS + 0.2% Triton X-100, Fisher). Intestines were then blocked in 1% BSA (Bio Basic) + 0.2%Triton X-100 (Fisher) and incubated in the same at room temperature for 2 hr with primary antibodies: mouse anti-Delta (Developmental Studies Hybridoma Bank [DSHB], 1:50), mouse anti-prospero (DSHB, 1:50), mouse anti-histone (Millipore, 1:2000), or rabbit anti-PER (generously provided by Patrick Emery, 1:1,500), then incubated at room temperature for 1 hr in secondary goat anti-mouse/rabbit antibodies (Life Technologies, 1:2000), and counterstained with DAPI (Thermo Fisher Scientific, 1:5,000). Samples were imaged using a slide scanner (Zeiss Axio Scan.Z1) that assembled single images consisting of merged and tiled z stacks of the entire tissue sample in a single plane of focus, or by confocal microscopy (Olympus IX81 FV1000) with a 60× water-immersion lens. Images were analyzed using Zen Blue Edition software (Zeiss) and processed using Photoshop CS5 (Adobe).

### Generation of Clock Reporters

The enhancer of Per and Tim were synthesized as gBlocks (Integrated DNA Technologies) in 2× tandem multiplexes. Then two copies of the gBlocks were amplified by PCR and inserted into a reporter vector using In-Fusion HD Cloning (Clontech) to generate 4× Per and 4× Tim enhancers (Figure S4). The reporter vector contains fly heat shock mini promoter and an nlsGFP fused with PEST domain at the C terminus (destabilized GFP) as previously used for the construction of Notch reporter(Hunter et al., 2016).

### RT-qPCR

Flies were synchronized to LD cycles as described above. At each time point, ∼10 intestines were dissected in PBS (Fisher) and stored in RNAlater reagent (Qiagen). Intestines were homogenized in RLT Buffer (Qiagen) using a Bullet blender as directed by the manufacturers protocol (Next Advance). RNA was extracted using the RNeasy Mini Kit (Qiagen). cDNA was prepared using ISCRIPT RT Supermix (Bio-Rad). qPCR was carried out using iTaq Universal SYBR Green Supermix (Bio-Rad) on the ViiA7 PCR plate reader.

### Quantification and Statistical Analysis

Fluorescence intensities were obtained using Zen Blue Software (Zeiss) and examined for changes in the ratio of average GFP:DAPI signal over time, except for Figure 3D, in which the total GFP:DAPI for only n = 5 individual cells of each cell type in a single 2-μm section was used. Whole-gut measurements include regions R1 to R5, anterior measurements include region R2 only, and posterior include R5 only. For confocal images, GFP measurements from single optical sections were quantified using ImageJ. Cell types were identified by antibody staining and morphology. Results are reported as mean ± SEM, and statistical significance was established using one-way ANOVA unless otherwise stated. All results were analyzed using Prism (GraphPad); n values refer to number of intestines, except for confocal image analysis, where n = number of cells.

For details of fly stocks and primers see Supplemental Experimental Procedures.

## Author Contributions

K.P. and N.B. conducted experiments, analyzed data, and wrote the paper. M.H., H.C., and L.H. conducted experiments. L.H. and N.P. designed the clock reporter vectors and helped write the figures. P.K. designed the clock reporter promoters, designed the experiments, and wrote the paper.

## Supplemental Information

Document S1. Supplemental Experimental Procedures and Figures S1–S3

Document S2. Article plus Supplemental Information
