# Supplementary material for: Intestinal Stem Cells Exhibit Conditional Circadian Clock Function
Source: Stem Cell Reports. 2018 Nov 13;11(5):1287–301. doi: 10.1016/j.stemcr.2018.10.010 (PMC6235668; doi:10.1016/j.stemcr.2018.10.010)
Supplement: Document S1. Supplemental Experimental Procedures and Figures S1–S3 [file mmc1.pdf]

Stem Cell Reports, Volume *11*

Supplemental Information

Intestinal Stem Cells Exhibit Conditional Circadian Clock Function

Kathyani Parasram, Nathaniel Bernardon, Maha Hammoud, Hanna Chang, Li He, Norbert Perrimon, and Phillip Karpowicz

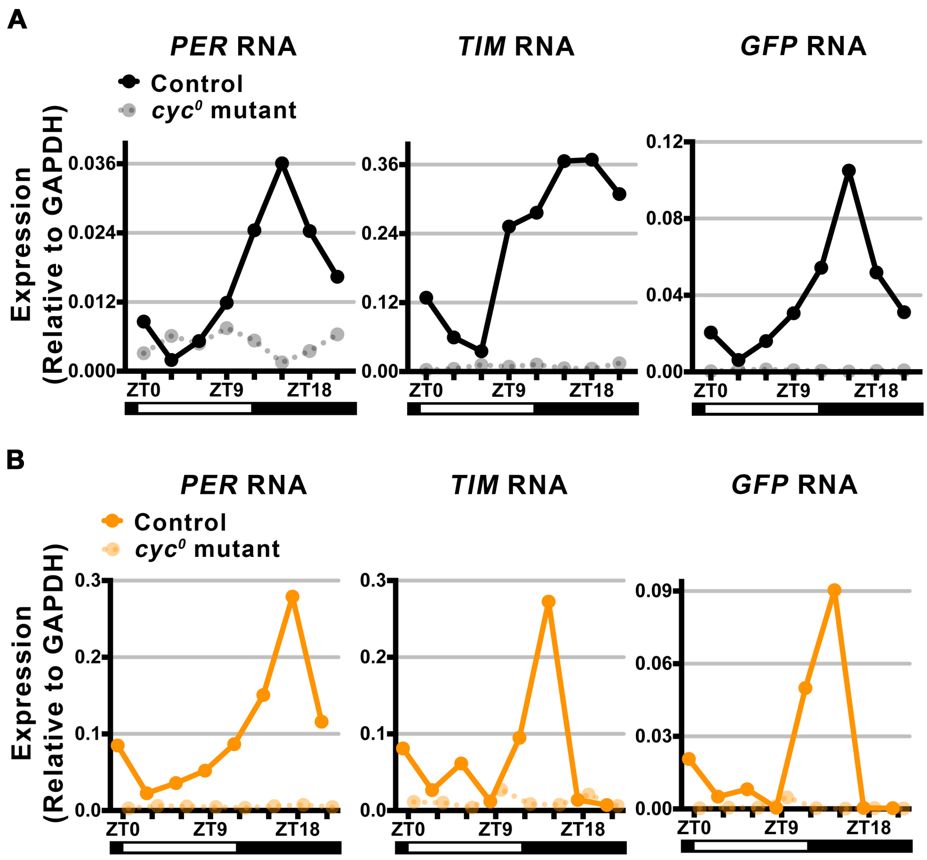

Figure S1: Analysis of ***Clock***^{PER} **and** ***Clock***^{TIM} **reporter transcription. Related to Figure 1. (A)** A second representative experiment showing RT-qPCR expression for *Clock*^{PER} (*GFP* RNA), *PER* and *TIM* RNA. Each data point represents a signal obtained from n=10 intestines. Data confirms the expression rhythms shown in Fig 1E. **(B)** RT-qPCR expression for *Clock*^{TIM} (*GFP* RNA), *PER* and *TIM* RNA. Each data point represents a signal obtained from n=10 intestines. As in Fig 1E and above, all three genes in show similar expression phases indicating similar timing of CLK/CYC activity on the *TIM* promoter as on the *PER* promoter.

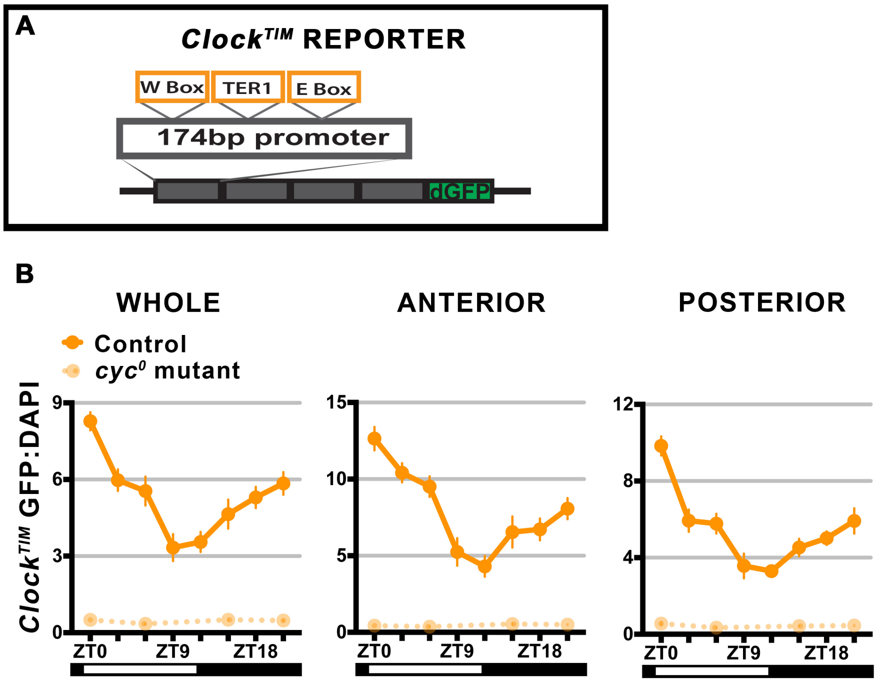

Figure S2: The *Clock*^{TIM} reporter reports circadian clock activity. Related to Figure 1 and Figure 2A. (A) Schematic of the *Clock*^{TIM} reporter shows four copies of the 174bp minimal promoter upstream of destabilized GFP (dGFP) to report temporal changes in circadian clock activity through *TIM* expression. **(B)** Analysis of *Clock*^{TIM} GFP:DAPI signal under LD photoperiod for the whole intestine, anterior (R2), and posterior (R5) regions. All regions show similar phase of clock reporter activity, and the *cyc*^{0} mutant has no circadian transactivation and is GFP low. Data presented as mean of n≥10 intestines, error bars show ± SEM. Control *vs*. *cyc*^{0}: whole gut (2-way ANOVA F=6.52, p=0.0006), anterior (2-way ANOVA F=7.172, p=0.0003), posterior (2-way ANOVA F=11.2, p<0.0001).

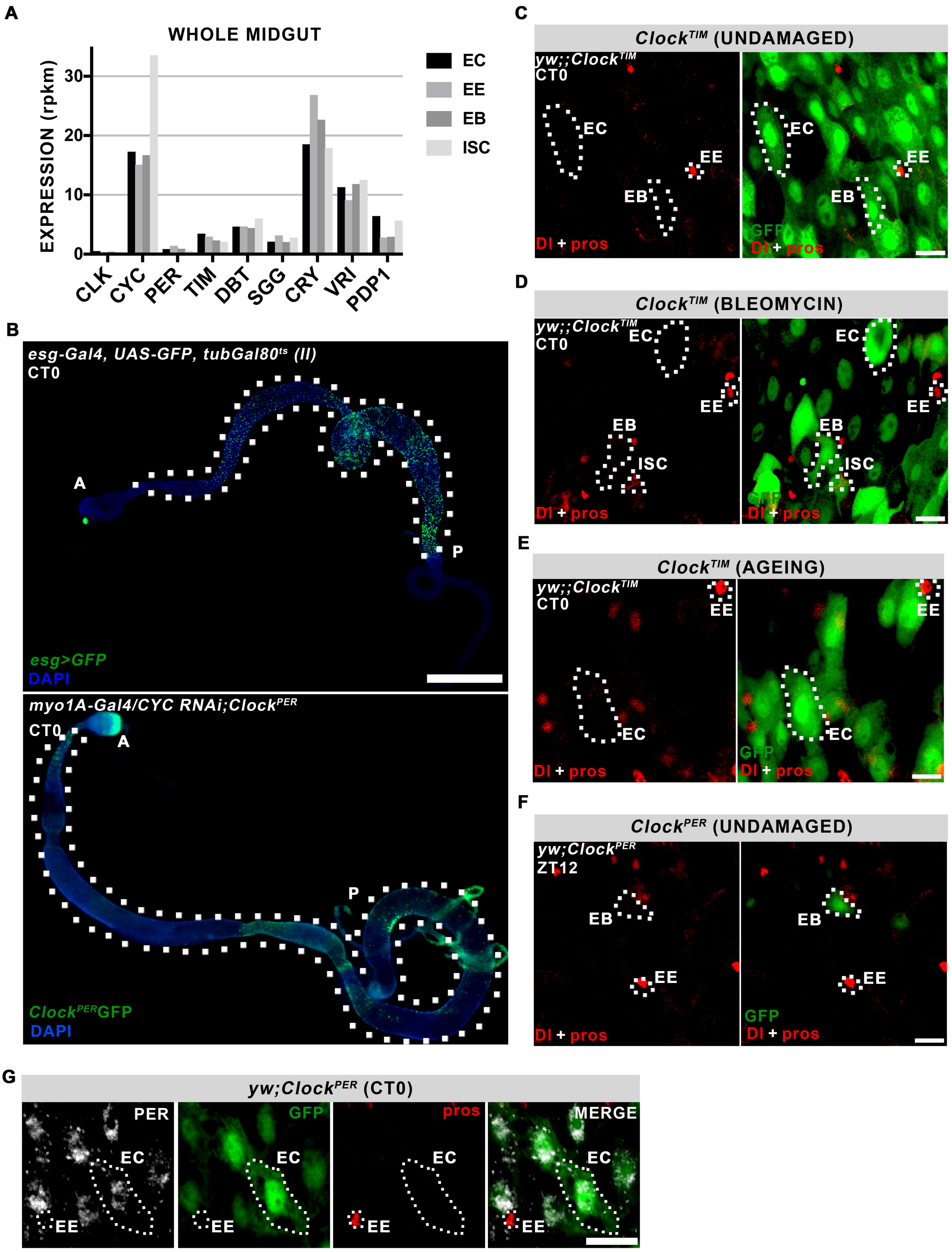

Figure S3: Single cell analysis of EE and ISC/EB cells in intestine. Related to Figure 2. (see next page)

Figure S3: Single cell analysis of EE and ISC/EB cells in intestine. Related to Figure 2. (A) Graph depicting the RNA-sequencing expression of clock genes modified from *flygutseq.buchonlab.com*[26]. Data shows similar gene expression levels for all clock genes in all four intestinal epithelial cell types, although we note that the expression of CLK is particularly low in this dataset. The phase of circadian clock activity and the dissection time is not available from this dataset which could greatly affect clock gene levels. **(B)** Representative images of *Drosophila* intestines at CT0 comparing the number of undifferentiated precursors marked by GFP in the *esg-Gal4, UAS-GFP, tubGal80^{TS}* genotype which marks all ISC/EBs when flies are shifted to 29°C temperature, *vs*. the number of undifferentiated precursors marked by *Clock^{PER}* GFP+ when *CYC* is knocked down in ECs (*Myo1A>CYC*) at its highest time of expression. Note that many small GFP+ cells that are present in the former, are absent in the latter. DAPI counterstains nuclei, A: indicates the anterior region, P: posterior. Scale bar is 500µm. **(C)** Representative confocal Z-stack of *Clock^{TIM}* at CT0 shows that EEs do not express GFP+ reporter signal from the *TIM* reporter either. **(D and E)** Confocal section of *Clock^{TIM}* at CT0 shows that EE cells do not express GFP from the *TIM* reporter under bleomycin-treated or ageing conditions. Dl+ and pros+ marking the ISCs and EEs, respectively, are outlined, as is a large polyploid EC and differentiating EB, as indicated, scale bar is 10µm. **(F)** Confocal section of *Clock^{PER}* at ZT12, the trough of GFP+ expression, shows that EE cells do not express *PER* reporter activity in an anti-phasic fashion either. An EE is outlined, as is an EB showing low levels of GFP+ signal at this time, scale bar is 10µm. **(G)** Confocal section showing nuclear PER antibody staining in ECs, but not EEs in the *Clock^{PER}* reporter that co-expresses PER and GFP simultaneously, indicating no circadian clock function. EEs do not show either expression of PER or GFP. Cells of interest are outlined: pros+ marks EEs, ECs are the large polyploid cells. Scale bar is 10µm.

Supplemental Experimental Procedures

Fly Strains

Flies were housed at 25°C under a 12-h light:12-h dark cycle (LD) on media containing 1.2% w/v dry yeast, 0.7% w/v soyflour, 5% w/v cornmeal, 0.4% w/v malt, 0.4% v/v agar, 5.3% v/v with propionic acid, and tegosept (Diamed). Experiments with temperature-induced expression (Fig 5) were carried out in the exact same conditions except at 29°C for 7 days. In all experiments, flies were tested following >5 days synchronization to LD schedules. In the free-running experiments, flies were moved to 24h dark conditions (DD) and tested the following day after moving, or 5 or 10 days after free-running DD conditions (as indicated in the Figures). Female flies <14 days old (at time of dissection) were used for experiments, except for ageing experiments which used female flies aged 35-40 days. For bleomycin-treatment, 2 days prior to the experiment flies were put on food as above but containing 25µg/mL bleomycin (Millipore). Restricted feeding was performed by moving flies from hydration-supplemented vials that contained 2.4g of agar in 500mL water to vials containing food (at the times indicated in the text).

CYC RNAi constructs, which have been previously validated for efficient cell-specific circadian disruption[S1], were used with the *esg-Gal4* driver to disrupt *CYC* in undifferentiated precursors (ISCs + EBs) or with the *Myo1A-Gal4* driver to disrupt *CYC* in differentiated ECs. Our previous work has also shown these *Gal4* drivers are not expressed in clock neurons, and hence do not affect circadian behavior [S1].

The following strains were used:
ClockPER on II (attP40)
ClockPER on III (attP2)
ClockTIM on II (attP40)
ClockTIM on III (attP2)
cyc^{0}, ry^{506}
$w^{1118};;cry^{01}$
esg-Gal4 (II)
myo1A-Gal4 (II)
yw; PER-HGM
w;TIM-HGM
w;;TIM-HGM
Luc RNAi (Bloomington stock center #31603)
cyc RNAi (Bloomington stock center #42563)
Mer RNAi (Bloomington stock center #28007)
APC RNAi (Bloomington stock center #34869)
N RNAi (Bloomington stock center #33611)
UAS-Yki

Experimental fly strains, organized by figure:

Figure		Fly Strain
1	C	$yw;Clock^{PER};$
	D	$yw;Clock^{PER};$
	E	$yw;Clock^{PER};$<br>$yw;Clock^{PER}/CyO;cyc^{0},ry^{506}/TM6B$
	F	$yw;Clock^{PER};$<br>$yw;Clock^{PER}/CyO;cyc^{0},ry^{506}/TM6B$
2	A	$yw;Clock^{PER};$<br>$yw;Clock^{PER}/CyO;cyc^{0},ry^{506}/TM6B$
	C	$yw;Clock^{PER};$
	D	$yw;Clock^{PER};$
	E	w^{1118}
	A	$yw;Clock^{PER};$

B	*yw;Clock*^{PER}*;*<br>*;Clock*^{PER}*;cry*^{01}
C	*yw;Clock*^{PER}*;*
D	*yw;Clock*^{PER}*;*
E	*yw;Clock*^{PER}*;*
A	*esg-Gal4,UAS-CD8RFP/Valium20-Cyc;Clock*^{PER}*/MKRS*
B	*Myo1A-Gal4/Valium20-Cyc;Clock*^{PER}*/MKRS*
C	*yw;Clock*^{PER}*;*
D	*yw;Clock*^{PER}*;*
E	*Myo1A-Gal4/IF;Clock*^{PER}*/Valium1-Luc*<br>*Myo1A-Gal4/Valium20-Cyc;Clock*^{PER}*/MKRS*<br>*esg-Gal4,UAS-CD8RFP/IF;Clock*^{PER}*/Valium1-Luc*<br>*esg-Gal4,UAS-CD8RFP/Valium20-Cyc;Clock*^{PER}*/MKRS*
F	same as 4E
G	same as 4E
A	*esg-Gal4/Clock*^{PER}*;tubGal80*^{TS}*/Valium20-Cyc*<br>*esg-Gal4/Clock*^{PER}*;tubGal80*^{TS}*/Valium20-APC*<br>*esg-Gal4/Clock*^{PER}*;tubGal80*^{TS}*/UAS-Yki*<br>*esg-Gal4/Clock*^{PER}*;tubGal80*^{TS}*/Valium20-N*
B	*esg-Gal4/Clock*^{PER}*;tubGal80*^{TS}*/+*<br>*esg-Gal4/Clock*^{PER}*;tubGal80*^{TS}*/Valium20-Cyc*<br>*esg-Gal4/Clock*^{PER}*;tubGal80*^{TS}*/Valium20-APC*<br>*esg-Gal4/Clock*^{PER}*;tubGal80*^{TS}*/Valium10-Mer*<br>*esg-Gal4/Clock*^{PER}*;tubGal80*^{TS}*/UAS-Yki*<br>*esg-Gal4/Clock*^{PER}*;tubGal80*^{TS}*/Valium20-N*<br>*esg-Gal4/Clock*^{TIM}*;tubGal80*^{TS}*/+*<br>*esg-Gal4/Clock*^{TIM}*;tubGal80*^{TS}*/Valium20-Cyc*<br>*esg-Gal4/Clock*^{TIM}*;tubGal80*^{TS}*/Valium20-APC*<br>*esg-Gal4/Clock*^{TIM}*;tubGal80*^{TS}*/Valium10-Mer*<br>*esg-Gal4/Clock*^{TIM}*;tubGal80*^{TS}*/UAS-Yki*<br>*esg-Gal4/Clock*^{TIM}*;tubGal80*^{TS}*/Valium20-N*
C	*esg-Gal4/Clock*^{PER}*;tubGal80*^{TS}*/Valium20-APC*
D	*esg-Gal4/Clock*^{PER}*;tubGal80*^{TS}*/Valium20-N*
A	*yw;Clock*^{PER}*;*<br>*yw;Clock*^{PER}*/CyO;cyc*^{0}*,ry*^{506}*/TM6B*
B	*w;Clock*^{TIM}<br>*yw;Clock*^{TIM}*/CyO;cyc*^{0}*,ry*^{506}*/TM6B*
B	*w;Clock*^{TIM}<br>*yw;Clock*^{TIM}*/CyO;cyc*^{0}*,ry*^{506}*/TM6B*
B	*esg-Gal4,UAS-GFP,tubGal80*^{TS}*;*<br>*Myo1A-Gal4/Valium20-Cyc;Clock*^{PER}*/MKRS*
C	*w;;Clock*^{TIM}
D	*w;;Clock*^{TIM}
E	*w;;Clock*^{TIM}
F	*yw;Clock*^{PER}
G	*yw;Clock*^{PER}

Generation of Clock reporters

The following primers were used:

gBlock1 forward 5' AACCAACAACTCTAGACTACTGC 3'

gBlock1 reverse 5' ACTGCTGAGTCAGACTATCTGGATCCTCTAGACGTGC 3'

gBlock1 forward 5' AGTCTGACTCAGCAGTAACCAACAACTCTAGACTACTGC 3'

gBlock1 reverse 5'ATCTGGATCCTCTAGACGTGC 3'

Sequence of Per enhancer:

CGAGTTCGCACAACTCATGGTGGGGCAGGGACGGGTCCGAGTGCAAAAAGCGTCCGAGAAACCGTAG
GCAGTGAAAAGCCGCCGCTCACGTGGCGAACTGCGTGACTTGGCCAGCAAATCCGC

Sequence of Tim enhancer:

TTCCGGCGCTGGTTATTCATGTTATCGAACATCGTAGTGGCGGTTGGCAAATAAACGTGCGGCACGTT
GTGATTACACGTGAGCCGATTTCCCCGGCCGTCCGGCATTGAGTGCGAGCGGGACGGAGCAGCGCGGC
GAGAGCTTTCGGCTAGCGTCTTTCGTTCGCACAAACGC

The full-length sequences for the plasmids can be found in the supplementary data file.

RT-qPCR

The following primers were used:

GFP Forward: TTTCATCACCCTCCTCCAAG

GFP Reverse: TTATGCCCGAGTTCTTTTCG

PER Forward: TCATCCAGAACGGTTGCTACG

PER Reverse: CCTGAAAGACGCGATGGTGT

TIM Forward: CCAGCATTCATTCCAAGCAG

TIM Reverse: GCGTGGCAAACTGTGTTATG

GAPDH Forward: CCAATGTCTCCGTTGTGGA

GAPDH Reverse: TCGGTGTAGCCCAGGATT

Tissue Fluorescence Imaging

Flies were synchronized to LD cycles as described above. At each time point ~10 intestines were dissected in PBS (Fisher), and fixed in 4% PFA (Electron microscopy sciences) in PBS for 40 minutes at room temperature. Intestines were rinsed 3x using PBS, and then counterstained with DAPI (ThermoFisher Scientific, 1:5000) in PBS-T (PBS + 0.2% Triton X-100, Fisher) for 5 minutes. Intestines were then washed with PBS-T and mounted on slides with ProLong Gold antifade reagent (ThermoFisher Scentific). Samples were imaged using a slide scanner (Zeiss Axio Scan.Z1) that assembled single images consisting of merged and tiled Z-stacks of the entire tissue sample in a single plane of focus. Images were analyzed using Zen Blue Edition software (Zeiss). Images were processed using Photoshop CS5 (Adobe).

Supplemental Reference

S1. Karpowicz, P., Zhang, Y., Hogenesch, J.B., Emery, P., and Perrimon, N. (2013). The circadian clock gates the intestinal stem cell regenerative state. Cell reports *3*, 996-1004.

Additional Items

A) 4xPER Sequence

```
LOCUS       Exported               11609 bp ds-DNA     circular SYN 17-SEP-2018
DEFINITION  synthetic circular DNA
ACCESSION   .
VERSION     .
KEYWORDS    4XPer nls-sfGFP-MODC
SOURCE      synthetic DNA construct
  ORGANISM  synthetic DNA construct
REFERENCE   1  (bases 1 to 11609)
  AUTHORS   Li
  TITLE     Direct Submission
  JOURNAL   Exported Sep 17, 2018 from SnapGene Viewer 4.1.5
            http://www.snapgene.com
FEATURES             Location/Qualifiers
     source          1..11609
                     /organism="synthetic DNA construct"
                     /mol_type="other DNA"
     protein_bind    77..507
                     /label=gypsy insulator
                     /bound_moiety="Su(Hw), CP190, and Mod(mdg4)67.2"
                     /note="chromatin insulator from Drosophila"
     misc_feature    522..643
                     /label=Per
     misc_feature    659..780
                     /label=Per
     misc_feature    807..928
                     /label=Per
     misc_feature    944..1065
                     /label=Per
     promoter        1122..1360
                     /label=hsp70 promoter
                     /note="Drosophila melanogaster hsp70Bb promoter"
     intron          1375..1441
                     /label=Mhc intron
                     /note="modified Drosophila myosin heavy chain intron 16
                     (Pfeiffer et al., 2010)"
     CDS             1467..2345
                     /codon_start=1
                     /label=nls-sfGFP-MODC
                     /translation="MPKKKRKVVSKGEELFTGVVPILVELDGDVNGHKFSVRGEGEGDA
                     TNGKLTLKFICTTGKLPVPWPTLVTTLTYGVQCFSRYPDHMKQHDFFKSAMPEGYVQER
                     TISFKDDGTYKTRAEVKFEGDTLVNRIELKGIDFKEDGNILGHKLEYNFNSHNVYITAD
                     KQKNGIKANFKIRHNVEDGSVQLADHYQQNTPIGDGPVLLPDNHYLSTQSVLSKDPNEK
                     RDHMVLLEFVTAAGITLGMDELYKTSYKKLSHGFPPEVEEQDDGTLPMSCAQESGMDRH
                     PAACASARINV"
     CDS             1470..1490
                     /codon_start=1
                     /product="nuclear localization signal of SV40 (simian virus
                     40) large T antigen"
                     /label=SV40 NLS
                     /translation="PKKKRKV"
```

```
     misc_feature    2363..3038
                     /label=p10
     protein_bind    3044..3474
                     /label=gypsy insulator
                     /bound_moiety="Su(Hw), CP190, and Mod(mdg4)67.2"
                     /note="chromatin insulator from Drosophila"
     misc_feature    8031..8616
                     /label=P element 5' end
     rep_origin      complement(8851..9439)
                     /direction=LEFT
                     /label=ori
                     /note="high-copy-number ColE1/pMB1/pBR322/pUC origin of
                     replication"
     CDS             complement(9610..10470)
                     /codon_start=1
                     /gene="bla"
                     /product="beta-lactamase"
                     /label=AmpR
                     /note="confers resistance to ampicillin, carbenicillin, and
                     related antibiotics"
                     /translation="MSIQHFRVALIPFFAAFCLPVFAHPETLVKVKDAEDQLGARVGYI
                     ELDLNSGKILESFRPEERFPMMSTFKVLLCGAVLSRIDAGQEQLGRRIHYSQNDLVEYS
                     PVTEKHLTDGMTVRELCSAAITMSDNTAANLLLTTIGGPKELTAFLHNMGDHVTRLDRW
                     EPELNEAIPNDERDTTMPVAMATTLRKLLTGELLTLASRQQLIDWMEADKVAGPLLRSA
                     LPAGWFIADKSGAGERGSRGIIAALGPDGKPSRIVVIYTTGSQATMDERNRQIAEIGAS
                     LIKHW"
     promoter        complement(10471..10575)
                     /gene="bla"
                     /label=AmpR promoter
     misc_feature    11377..11609
                     /label=P element 3' end
                     /note="P element 3' end"
ORIGIN
        1 aagcttgggc tgcaggtcga cctcgaggcc tcgagttaac gttacgttaa cgttaacgtt
       61 cgaggtcgac tctagttggc cacgtaataa gtgtgcgttg aatttattcg caaaaacatt
      121 gcatattttc ggcaaagtaa aattttgttg catacttat caaaaaataa gtgctgcata
      181 ctttttagag aaaccaaata attttttatt gcatacccgt ttttaataaa atacattgca
      241 taccctcttt taataaaaaa tattgcatac tttgacgaaa caaattttcg ttgcataccc
      301 aataaaagat tattatattg catacccgtt tttaataaaa tacattgcat accctctttt
      361 aataaararat attgcatacg ttgacgaaac aaattttcgt tgcataccca ataaaagatt
      421 attatattgc ataccttttc ttgccatacc atttagccga tcaattgtgc tcggcaacag
      481 tatatttgtg gtgtgccaac caacaactct agactactgc cgagttcgca caactcatgg
      541 tggggcaggg acgggtccga gtgcaaaaag cgtccgagaa accgtaggca gtgaaaagcc
      601 gccgctcacg tggcgaactg cgtgacttgg ccagcaaatc cgcacatggt acctcgacga
      661 gttcgcacaa ctcatggtgg ggcagggacg ggtccgagtg caaaaagcgt ccgagaaacc
      721 gtaggcagtg aaaagccgcc gctcacgtgg cgaactgcgt gacttggcca gcaaatccgc
      781 gcacgaagga tctctagact actgccgagt tcgcacaact catggtgggg cagggacggg
      841 tccgagtgca aaaagcgtcc gagaaaccgt aggcagtgaa aagccgccgc tcacgtggcg
      901 aactgcgtga cttggccagc aaatccgcac atggtacctc gacgagttcg cacaactcat
      961 ggtggggcag ggacgggtcc gagtgcaaaa agcgtccgag aaaccgtagg cagtgaaaag
     1021 ccgccgctca cgtggcgaac tgcgtgactt ggccagcaaa tccgcgcacg agatccagat
     1081 ccactagcga gcggagactc tagcgctagc gacgtcgagc gccggagtat aaatagaggc
     1141 gcttcgtcta cggagcgaca attcaattca aacaagcaaa gtgaacacgt cgctaagcga
     1201 aagctaagca aataaacaag cgcagctgaa caagctaaac aatctgcagt aaagtgcaag
```

1261 ttaaagtgaa tcaattaaaa gtaaccagca accaagtaaa tcaactgcaa ctactgaaat
1321 ctgccaagaa gtaattattg aatacaagaa gagaactctg aatagatcta aaaggtaggt
1381 tcaaccactg atgcctaggc acaccgaaac gactaaccct aattcttatc ctttacttca
1441 ggcggaactt aaaaaaaaaa atcaaaatgc ccaagaagaa gcgcaaggtg gtgtccaagg
1501 gcgaggagct gttcaccggc gtggtgccca tcctggtgga gctggatggc gacgtgaacg
1561 gccacaagtt cagcgtgcgc ggcgagggcg agggcgacgc caccaacggc aagctgaccc
1621 tgaagttcat ctgcaccacc ggcaagctgc ccgtgccctg gcccaccctg gtgaccaccc
1681 tgacctacgg cgtgcagtgc ttcagccgct accccgatca catgaagcag cacgatttct
1741 tcaagagcgc catgcccgag ggctacgtgc aggagcgcac catcagcttc aaggatgacg
1801 gcacctacaa gacccgcgcc gaggtgaagt tcgagggcga taccctggtg aaccgcatcg
1861 agctgaaggg catcgatttc aaggaggatg gcaacatcct gggccacaag ctggagtaca
1921 acttcaacag ccacaacgtg tacatcaccg ccgataagca gaagaacggc atcaaggcca
1981 acttcaagat ccgccacaat gtggaggatg gctccgtgca gctggccgat cactaccagc
2041 agaacacccc catcggcgac ggcccagtgc tgctgcccga taaccactac ctgagcaccc
2101 agagcgtgct gtccaaggac cccaacgaga agcgcgatca catggtgctg ctggagttcg
2161 tgaccgccgc cggcatcacc ctgggcatgg atgagctgta caagactagt tacaagaagc
2221 ttagccatgg cttcccgccg gaggtggagg agcaggatga tggcacgctg cccatgtctt
2281 gtgcccagga gagcgggatg gaccgtcacc ctgcagcctg tgcttctgct aggatcaatg
2341 tgtagatgcg cctagttaat agaatgaatc gtttttaaaa taacaaatca attgttttat
2401 aatattcgta cgattctttg attatgtaat aaaatgtgat cattaggaag attacgaaaa
2461 atataaaaaa tatgagttct gtgtgtataa caaatgctgt aaacgccaca attgtgtttg
2521 ttgcaaataa acccatgatt atttgattaa aattgttgtt ttctttgttc atagacaata
2581 gtgtgttttg cctaaacgtg tactgcataa actccatgcg agtgtatagc gagctagtgg
2641 ctaacgcttg ccccaccaaa gtagattcgt caaaatcctc aatttcatca ccctcctcca
2701 agtttaacat ttggccgtcg gaattaactt ctaaagatgc cacataatct aataaatgaa
2761 atagagattc aaacgtggcg tcatcgtccg tttcgaccat ttccgaaaag aactcgggca
2821 taaactctat gatttctctg gacgtggtgt tgtcgaaact ctcaaagtac gcagtcagga
2881 acgtgcgcga catgtcgtcg ggaaactcgc gcggaaacat gttgttgtaa ccgaacgggt
2941 cccatagcgc caaaaccaaa tctgccagcg tcaatagaat gagcacgatg ccgacaatgg
3001 agctggcttg gatagcgatt cgagttaacg gccggccgct agttggccac gtaataagtg
3061 tgcgttgaat ttattcgcaa aaacattgca tattttcggc aaagtaaaat tttgttgcat
3121 accttatcaa aaaataagtg ctgcatactt tttagagaaa ccaaataatt ttttattgca
3181 tacccgtttt taataaaata cattgcatac cctcttttaa taaaaaatat tgcatacttt
3241 gacgaaacaa attttcgttg catacccaat aaaagattat tatattgcat acccgttttt
3301 aataaaatac attgcatacc ctcttttaat aaaraatatt gcatacgttg acgaaacaaa
3361 ttttcgttgc atacccaata aaagattatt atattgcata ccttttcttg ccataccatt
3421 tagccgatca attgtgctcg gcaacagtat atttgtggtg tgccaaccaa caactctagt
3481 gctgcatcca acgcgttggg agctctccgg atcaattcgg cttcaggtac cgtcgacgat
3541 gtaggtcacg gtctcgaagc cgcggtgcgg gtgccagggc gtgcccttgg gctccccggg
3601 cgcgtactcc acctcaccca tctggtccat catgatgaac gggtcgaggt ggcggtagtt
3661 gatcccggcg aacgcgcggc gcaccgggaa gccctcgccc tcgaaaccgc tgggcgcggt
3721 ggtcacggtg agcacgggac gtgcgacggc gtcggcgggt gcggatacgc ggggcagcgt
3781 cagcgggttc tcgacggtca cggcgggcat gtcgacaagc cgaattgatc cactagaagg
3841 cctaattcgc ggccgcggat ctgatcccgg gcgggtaccg aattctagta tgtatgtaag
3901 ttaataaaac cctttttttgg agaatgtaga tttaaaaaaa catatttttt ttttattttt
3961 tactgcactg gatatcattg aacttatctg atcagtttta aatttacttc gatccaaggg
4021 tatttgaagt accaggttct ttcgattacc tctcactcaa aatgacattc cactcaaagt
4081 cagcgctgtt tgcctccttc tctgtccaca gaaatatcgc cgtctctttc gccgctgcgt
4141 ccgctatctc tttcgccacc gtttgtagcg ttacctagcg tcaatgtccg ccttcagttg
4201 cactttgtca gcggtttcgt gacgaagctc caagcggttt acgccatcaa ttaaacacaa
4261 agtgctgtgc caaaactcct ctcgcttctt atttttgttt gtttttttgag tgattggggt
4321 ggtgattggt tttgggtggg taagcagggg aaagtgtgaa aaatcccggc aatggccaa
4381 gaggatcagg agctattaat tcgcggaggc agcaaacacc catctgccga gcatctgaac
4441 aatgtgagta gtacatgtgc atacatctta agttcacttg atctatagga actgcgattg

4501 caacatcaaa ttgtctgcgg cgtgagaact gcgacccaca aaaatcccaa accgcaatcg
4561 cacaaacaaa tagtgacacg aaacagatta ttctggtagc tgtgctcgct atataagaca
4621 atttttaaga tcatatcatg atcaagacat ctaaaggcat tcattttcga ctacattctt
4681 ttttacaaaa aatataacaa ccagatattt taagctgatc ctagatgcac aaaaaataaa
4741 taaaagtata aacctacttc gtaggatact tcgttttgtt cggggttaga tgagcataac
4801 gcttgtagtt gatatttgag atcccctatc attgcagggt gacagcggac gcttcgcaga
4861 gctgcattaa ccagggcttc gggcaggcca aaaactacgg cacgctcctg ccacccagtc
4921 cgccggagga ctccggttca gggagcggcc aactagccga gaacctcacc tatgcctggc
4981 acaatatgga catctttggg gcggtcaatc agccgggctc cggatggcgg cagctggtca
5041 accggacacg cggactattc tgcaacgagc gacacatacc ggcgcccagg aaacatttgc
5101 tcaagaacgg tgagtttcta ttcgcagtcg gctgatctgt gtgaaatctt aataaagggt
5161 ccaattacca atttgaaact cagtttgcgg cgtggcctat ccgggcgaac ttttggccgt
5221 gatgggcagt tccggtgccg gaaagacgac cctgctgaat gcccttgcct ttcgatcgcc
5281 gcagggcatc caagtatcgc catccgggat gcgactgctc aatggccaac ctgtggacgc
5341 caaggagatg caggccaggt gcgcctatgt ccagcaggat gacctcttta tcggctccct
5401 aacggccagg gaacacctga ttttccaggc catggtgcgg atgccacgac atctgaccta
5461 tcggcagcga gtggcccgcg tggatcaggt gatccaggag ctttcgctca gcaaatgtca
5521 gcacacgatc atcggtgtgc ccggcagggt gaaaggtctg tccggcggag aaaggaagcg
5581 tctggcattc gcctccgagg cactaaccga tccgccgctt ctgatctgcg atgagcccac
5641 ctccggactg gactcattta ccgcccacag cgtcgtccag gtgctgaaga agctgtcgca
5701 gaagggcaag accgtcatcc tgaccattca tcagccgtct tccgagctgt ttgagctctt
5761 tgacaagatc cttctgatgg ccgagggcag ggtagctttc ttgggcactc ccagcgaagc
5821 cgtcgacttc ttttcctagt gagttcgatg tgtttattaa gggtatctag cattacatta
5881 catctcaact cctatccagc gtgggtgccc agtgtcctac caactacaat ccggcggact
5941 tttacgtaca ggtgttggcc gttgtgcccg gacgggagat cgagtcccgt gatcggatcg
6001 ccaagatatg gcgacaattt tgctattagc aaagtagccc gggatatgga gcagttgttg
6061 gccaccaaaa atttggagaa gccactggag cagccggaga atgggtacac ctacaaggcc
6121 acctggttca tgcagttccg ggcggtcctg tggcgatcct ggctgtcggt gctcaaggaa
6181 ccactcctcg taaaagtgcg acttattcag acaacggtga gtggttccag tggaaacaaa
6241 tgatataacg cttacaattc ttggaaacaa attcgctaga ttttagttag aattgcctga
6301 ttccacaccc ttcttagttt ttttcaatga gatgtatagt ttatagtttt gcagaaaata
6361 aataaatttc atttaactcg cgaacatgtt gaagatatga atattaatga gatgcgagta
6421 acattttaat ttgcagatgg ttgccatctt gattggcctc atctttttgg gccaacaact
6481 cacgcaagtg ggcgtgatga atatcaacgg agccatcttc ctcttcctga ccaacatgac
6541 ctttcaaaac gtctttgcca cgataaatgt aagtcttgtt tagaatacat ttgcatatta
6601 ataatttact aactttctaa tgaatcgatt cgatttaggt gttcacctca gagctgccag
6661 tttttatgag ggaggcccga agtcgacttt atcgctgtga cacatacttt ctgggcaaaa
6721 cgattgccga attaccgctt tttctcacag tgccactggt cttcacggcg attgcctatc
6781 cgatgatcgg actgcgggcc ggagtgctgc acttcttcaa ctgcctggcg ctggtcactc
6841 tggtggccaa tgtgtcaacg tccttcggat atctaatatc ctgcgccagc tcctcgacct
6901 cgatggcgct gtctgtgggt ccgccggtta tcataccatt cctgctcttt ggcggcttct
6961 tcttgaactc gggctcggtg ccagtatacc tcaaatggtt gtcgtacctc tcatggttcc
7021 gttacgccaa cgagggtctg ctgattaacc aatgggcgga cgtggagccg ggcgaaatta
7081 gctgcacatc gtcgaacacc acgtgcccca gttcgggcaa ggtcatcctg gagacgctta
7141 acttctccgc cgccgatctg ccgctggact acgtgggtct ggccattctc atcgtgagct
7201 tccgggtgct cgcatatctg gctctaagac ttcgggcccg acgcaaggag tagccgacat
7261 atatccgaaa taactgcttg tttttttttt taccattatt accatcgtgt ttactgttta
7321 ttgccccctc aaaaagctaa tgtaattata tttgtgccaa taaaaacaag atatgaccta
7381 tagaatacaa gtatttcccc ttcgaacatc cccacaagta gactttggat ttgtcttcta
7441 accaaaagac ttacacacct gcataccttа catcaaaaac tcgtttatcg ctacataaaa
7501 caccgggata tatttttat atacatactt ttcaaatcgc gcgccctctt cataattcac
7561 ctccaccaca ccacgtttcg tagttgctct ttcgctgtct cccacccgct ctccgcaaca
7621 cattcacctt ttgttcgacg accttggagc gactgtcgtt agttccgcgc gattcggttc
7681 gctcaaatgg ttccgagtgg ttcatttcgt ctcaatagaa attagtaata aatatttgta

7741 tgtacaattt atttgctcca atatatttgt atatatttcc ctcacagcta tatttattct
7801 aatttaatat tatgactttt taaggtaatt ttttgtgacc tgttcggagt gattagcgtt
7861 acaatttgaa ctgaaagtga catccagtgt ttgttccttg tgtagatgca tctcaaaaaa
7921 atggtgggca taatagtgtt gtttatatat atcaaaaata acaactataa taataagaat
7981 acatttaatt tagaaaatgc ttggatttca ctggaactag aattaattcg gctgctgctc
8041 taaacgacgc atttcgtact ccaaagtacg aatttttttcc ctcaagctct tattttcatt
8101 aaacaatgaa caggacctaa cgcacagtca cgttattgtt tacataaatg atttttttta
8161 ctattcaaac ttactctgtt tgtgtactcc cactggtata gccttctttt atcttttctg
8221 gttcaggctc tatcacttta ctaggtacgg catctgcgtt gagtcgcctc cttttaaatg
8281 tctgaccttt tgcaggtgca gccttccact gcgaatcttt aaagtgggta tcacaaattt
8341 gggagttttc accaaggctg cacccaaggc tctgctccca caattttctc ttaatagcac
8401 acttcggcac gtgaattaat tttactccag tcacagcttt gcagcaaaat ttgcaatatt
8461 tcattttttt ttattccacg taagggttaa tgttttcaaa aaaaaattcg tccgcacaca
8521 acctttcctc tcaacaagca aacgtgcact gaatttaagt gtatacttcg gtaagcttcg
8581 gctatcgacg ggaccacctt atgttatttc atcatgggcc agacccacgt agtccagcgg
8641 cagatcggcg gcggagaagt taagcgtctc caggatgacc ttgcccgaac tggggcacgt
8701 ggtgttcgac gatgtgcagc taatttcgcc cggctccacg tccgcccatt ggttaatcag
8761 cagaccctcg ttggcgtaac ggaaccatga gaggtacgac aaccatttga ggtatactgg
8821 caccgagccc gagttcaaga agaaggcgtt tttccatagg ctccgccccc ctgacgagca
8881 tcacaaaaat cgacgctcaa gtcagaggtg gcgaaacccg acaggactat aaagatacca
8941 ggcgtttccc cctggaagct ccctcgtgcg ctctcctgtt ccgaccctgc cgcttaccgg
9001 atacctgtcc gcctttctcc cttcgggaag cgtggcgctt tctcaatgct cacgctgtag
9061 gtatctcagt tcggtgtagg tcgttcgctc caagctgggc tgtgtgcacg aaccccccgt
9121 tcagcccgac cgctgcgcct tatccggtaa ctatcgtctt gagtccaacc cggtaagaca
9181 cgacttatcg ccactggcag cagccactgg taacaggatt agcagagcga ggtatgtagg
9241 cggtgctaca gagttcttga agtggtggcc taactacggc tacactagaa ggacagtatt
9301 tggtatctgc gctctgctga agccagttac cttcggaaaa agagttggta gctcttgatc
9361 cggcaaacaa accaccgctg gtagcggtgg tttttttgtt tgcaagcagc agattacgcg
9421 cagaaaaaaa ggatctcaag aagatccttt gatcttttct acggggtctg acgctcagtg
9481 gaacgaaaac tcacgttaag ggattttggt catgagatta tcaaaaagga tcttcaccta
9541 gatcctttta aattaaaaat gaagttttaa atcaatctaa agtatatatg agtaaacttg
9601 gtctgacagt taccaatgct taatcagtga ggcacctatc tcagcgatct gtctatttcg
9661 ttcatccata gttgcctgac tccccgtcgt gtagataact acgatacggg agggcttacc
9721 atctggcccc agtgctgcaa tgataccgcg agacccacgc tcaccggctc cagatttatc
9781 agcaataaac cagccagccg gaagggccga gcgcagaagt ggtcctgcaa ctttatccgc
9841 ctccatccag tctattaatt gttgccggga agctagagta agtagttcgc cagttaatag
9901 tttgcgcaac gttgttgcca ttgctacagg catcgtggtg tcacgctcgt cgtttggtat
9961 ggcttcattc agctccggtt cccaacgatc aaggcgagtt acatgatccc ccatgttgtg
10021 caaaaaagcg gttagctcct tcggtcctcc gatcgttgtc agaagtaagt tggccgcagt
10081 gttatcactc atggttatgg cagcactgca taattctctt actgtcatgc catccgtaag
10141 atgcttttct gtgactggtg agtactcaac caagtcattc tgagaatagt gtatgcggcg
10201 accgagttgc tcttgcccgg cgtcaatacg ggataatacc gcgccacata gcagaacttt
10261 aaaagtgctc atcattggaa aacgttcttc ggggcgaaaa ctctcaagga tcttaccgct
10321 gttgagatcc agttcgatgt aacccactcg tgcacccaac tgatcttcag catcttttac
10381 tttcaccagc gtttctgggt gagcaaaaac aggaaggcaa aatgccgcaa aaaagggaat
10441 aagggcgaca cggaaatgtt gaatactcat actcttcctt tttcaatatt attgaagcat
10501 ttatcagggt tattgtctca tgagcggata catatttgaa tgtatttaga aaaataaaca
10561 aataggggtt ccgcgcacat ttccccgaaa agtgccacct gacgtctaag aaaccattat
10621 tatcatgaca ttaacctata aaaataggcg tatcacgagg ccctttcgtc tcgcgcgttt
10681 cggtgatgac ggtgaaaacc tctgacacat gcagctcccg gagacggtca cagcttgtct
10741 gtaagcggat gccgggagca gacaagcccg tcagggcgcg tcagcgggtg ttggcgggtg
10801 tcggggctgg cttaactatg cggcatcaga gcagattgta ctgagagtgc accatatgcg
10861 gtgtgaaata ccgcaccgaa tcgcgcggaa ctaacgacag tcgctccaag gtcgtcgaac
10921 aaaaggtgaa tgtgttgcgg agagcgggtg ggagacagcg aaagagcaac tacgaaacgt

```
    10981 ggtgtggtgg aggtgaatta tgaagagggc gcgcgatttg aaaagtatgt atataaaaaa
    11041 tatatcccgg tgttttatgt agcgataaac gagtttttga tgtaaggtat gcaggtgtgt
    11101 aagtcttttg gttagaagac aaatccaaag tctacttgtg gggatgttcg aaggggaaat
    11161 acttgtattc tataggtcat atcttgtttt tattggcaca aatataatta cattagcttt
    11221 ttgagggggc aataaacagt aaacacgatg gtaataatgg taaaaaaaaa aacaagcagt
    11281 tatttcggat atatgtcggc tactccttgc gtcgggcccg aagtcttaga gccagatatg
    11341 cgagcacccg gaagctcacg atgagaatgg ccagaccatg atgaaataac ataaggtggt
    11401 cccgtcggca agagacatcc acttaacgta tgcttgcaat aagtgcgagt gaaaggaata
    11461 gtattctgag tgtcgtattg agtctgagtg agacagcgat atgattgttg attaaccctt
    11521 agcatgtccg tggggtttga attaactcat aatattaatt agacgaaatt atttttaaag
    11581 ttttattttt aataatttgc gagtacgca
//
```

B) 4xTIM Sequence

```
LOCUS       Exported               11816 bp ds-DNA     circular SYN 17-SEP-2018
DEFINITION  synthetic circular DNA
ACCESSION   .
VERSION     .
KEYWORDS    4XTim nls-sfGFP-MODC
SOURCE      synthetic DNA construct
  ORGANISM  synthetic DNA construct
REFERENCE   1  (bases 1 to 11816)
  AUTHORS   Li
  TITLE     Direct Submission
  JOURNAL   Exported Sep 17, 2018 from SnapGene Viewer 4.1.5
            http://www.snapgene.com
FEATURES             Location/Qualifiers
     source          1..11816
                     /organism="synthetic DNA construct"
                     /mol_type="other DNA"
     protein_bind    77..507
                     /label=gypsy insulator
                     /bound_moiety="Su(Hw), CP190, and Mod(mdg4)67.2"
                     /note="chromatin insulator from Drosophila"
     misc_feature    521..694
                     /label=Tim
     misc_feature    709..882
                     /label=Tim
     misc_feature    904..1077
                     /label=Tim
     misc_feature    1092..1265
                     /label=Tim
     promoter        1329..1567
                     /label=hsp70 promoter
                     /note="Drosophila melanogaster hsp70Bb promoter"
     intron          1582..1648
                     /label=Mhc intron
                     /note="modified Drosophila myosin heavy chain intron 16
                     (Pfeiffer et al., 2010)"
     CDS             1674..2552
                     /codon_start=1
                     /label=nls-GFP-MODC
                     /translation="MPKKKRKVVSKGEELFTGVVPILVELDGDVNGHKFSVRGEGEGDA
                     TNGKLTLKFICTTGKLPVPWPTLVTTLTYGVQCFSRYPDHMKQHDFFKSAMPEGYVQER
                     TISFKDDGTYKTRAEVKFEGDTLVNRIELKGIDFKEDGNILGHKLEYNFNSHNVYITAD
                     KQKNGIKANFKIRHNVEDGSVQLADHYQQNTPIGDGPVLLPDNHYLSTQSVLSKDPNEK
                     RDHMVLLEFVTAAGITLGMDELYKTSYKKLSHGFPPEVEEQDDGTLPMSCAQESGMDRH
                     PAACASARINV"
     CDS             1677..1697
                     /codon_start=1
                     /product="nuclear localization signal of SV40 (simian virus
                     40) large T antigen"
                     /label=SV40 NLS
                     /translation="PKKKRKV"
     misc_feature    2570..3245
                     /label=p10
```

```
     protein_bind    3251..3681
                     /label=gypsy insulator
                     /bound_moiety="Su(Hw), CP190, and Mod(mdg4)67.2"
                     /note="chromatin insulator from Drosophila"
     misc_feature    8238..8823
                     /label=P element 5' end
     rep_origin      complement(9058..9646)
                     /direction=LEFT
                     /label=ori
                     /note="high-copy-number ColE1/pMB1/pBR322/pUC origin of
                     replication"
     CDS             complement(9817..10677)
                     /codon_start=1
                     /gene="bla"
                     /product="beta-lactamase"
                     /label=AmpR
                     /note="confers resistance to ampicillin, carbenicillin, and
                     related antibiotics"
                     /translation="MSIQHFRVALIPFFAAFCLPVFAHPETLVKVKDAEDQLGARVGYI
                     ELDLNSGKILESFRPEERFPMMSTFKVLLCGAVLSRIDAGQEQLGRRIHYSQNDLVEYS
                     PVTEKHLTDGMTVRELCSAAITMSDNTAANLLLTTIGGPKELTAFLHNMGDHVTRLDRW
                     EPELNEAIPNDERDTTMPVAMATTLRKLLTGELLTLASRQQLIDWMEADKVAGPLLRSA
                     LPAGWFIADKSGAGERGSRGIIAALGPDGKPSRIVVIYTTGSQATMDERNRQIAEIGAS
                     LIKHW"
     promoter        complement(10678..10782)
                     /gene="bla"
                     /label=AmpR promoter
     misc_feature    11584..11816
                     /label=P element 3' end
                     /note="P element 3' end"
ORIGIN
        1 aagcttgggc tgcaggtcga cctcgaggcc tcgagttaac gttacgttaa cgttaacgtt
       61 cgaggtcgac tctagttggc cacgtaataa gtgtgcgttg aatttattcg caaaaacatt
      121 gcatattttc ggcaaagtaa aattttgttg catacettat caaaaaataa gtgctgcata
      181 ctttttagag aaaccaaata attttttatt gcatacccgt ttttaataaa atacattgca
      241 taccctcttt taataaaaaa tattgcatac tttgacgaaa caaattttcg ttgcataccc
      301 aataaaagat tattatattg catacccgtt tttaataaaa tacattgcat accctctttt
      361 aataaaraat attgcatacg ttgacgaaac aaattttcgt tgcataccca ataaaagatt
      421 attatattgc ataccttttc ttgccatacc atttagccga tcaattgtgc tcggcaacag
      481 tatatttgtg gtgtgccaac caacaactct agactactgc ttccggcgct ggttattcat
      541 gttatcgaac atcgtagtgg cggttggcaa ataaacgtgc ggcacgttgt gattacacgt
      601 gagccgattt ccccggccgt ccggcattga gtgcgagcgg gacggagcag cgcggcgaga
      661 gctttcggct agcgtctttc gttcgcacaa acgcacatgg tacctcgatt ccggcgctgg
      721 ttattcatgt tatcgaacat cgtagtggcg gttggcaaat aaacgtgcgg cacgttgtga
      781 ttacacgtga gccgatttcc ccggccgtcc ggcattgagt gcgagcggga cggagcagcg
      841 cggcgagagc tttcggctag cgtctttcgt tcgcacaaac gcgcacgatc tagagactac
      901 tgcttccggc gctggttatt catgttatcg aacatcgtag tggcggttgg caaataaacg
      961 tgcggcacgt tgtgattaca cgtgagccga tttccccggc cgtccggcat tgagtgcgag
     1021 cgggacggag cagcgcggcg agagctttcg gctagcgtct ttcgttcgca caaacgcaca
     1081 tggtacctcg attccggcgc tggttattca tgttatcgaa catcgtagtg gcggttggca
     1141 aataaacgtg cggcacgttg tgattacacg tgagccgatt tccccggccg tccggcattg
     1201 agtgcgagcg ggacggagca gcgcggcgag agctttcggc tagcgtcttt cgttcgcaca
     1261 aacgcgcacg atctagagga tccagatcca ctagcgagcg gagactctag cgctagcgac
     1321 gtcgagcgcc ggagtataaa tagaggcgct tcgtctacgg agcgacaatt caattcaaac
```

1381 aagcaaagtg aacacgtcgc taagcgaaag ctaagcaaat aaacaagcgc agctgaacaa
1441 gctaaacaat ctgcagtaaa gtgcaagtta aagtgaatca attaaaagta accagcaacc
1501 aagtaaatca actgcaacta ctgaaatctg ccaagaagta attattgaat acaagaagag
1561 aactctgaat agatctaaaa ggtaggttca accactgatg cctaggcaca ccgaaacgac
1621 taaccctaat tcttatcctt tacttcaggc ggaacttaaa aaaaaaaatc aaaatgccca
1681 agaagaagcg caaggtggtg tccaagggcg aggagctgtt caccggcgtg gtgcccatcc
1741 tggtggagct ggatggcgac gtgaacggcc acaagttcag cgtgcgcggc gagggcgagg
1801 gcgacgccac caacggcaag ctgaccctga agttcatctg caccaccggc aagctgcccg
1861 tgccctggcc caccctggtg accaccctga cctacggcgt gcagtgcttc agccgctacc
1921 ccgatcacat gaagcagcac gatttcttca agagcgccat gcccgagggc tacgtgcagg
1981 agcgcaccat cagcttcaag gatgacggca cctacaagac ccgcgccgag gtgaagttcg
2041 agggcgatac cctggtgaac cgcatcgagc tgaagggcat cgatttcaag gaggatggca
2101 acatcctggg ccacaagctg gagtacaact tcaacagcca caacgtgtac atcaccgccg
2161 ataagcagaa gaacggcatc aaggccaact tcaagatccg ccacaatgtg gaggatggct
2221 ccgtgcagct ggccgatcac taccagcaga acacccccat cggcgacggc ccagtgctgc
2281 tgcccgataa ccactacctg agcacccaga gcgtgctgtc caaggacccc aacgagaagc
2341 gcgatcacat ggtgctgctg gagttcgtga ccgccgccgg catcaccctg ggcatggatg
2401 agctgtacaa gactagttac aagaagctta gccatggctt cccgccggag gtggaggagc
2461 aggatgatgg cacgctgccc atgtcttgtg cccaggagag cgggatggac cgtcaccctg
2521 cagcctgtgc ttctgctagg atcaatgtgt agatgcgcct agttaataga atgaatcgtt
2581 tttaaaataa caaatcaatt gttttataat attcgtacga ttctttgatt atgtaataaa
2641 atgtgatcat taggaagatt acgaaaaata taaaaaatat gagttctgtg tgtataacaa
2701 atgctgtaaa cgccacaatt gtgtttgttg caaataaacc catgattatt tgattaaaat
2761 tgttgttttc tttgttcata gacaatagtg tgttttgcct aaacgtgtac tgcataaact
2821 ccatgcgagt gtatagcgag ctagtggcta acgcttgccc caccaaagta gattcgtcaa
2881 aatcctcaat ttcatcaccc tcctccaagt ttaacatttg gccgtcggaa ttaacttcta
2941 aagatgccac ataatctaat aaatgaaata gagattcaaa cgtggcgtca tcgtccgttt
3001 cgaccatttc cgaaaagaac tcgggcataa actctatgat ttctctggac gtggtgttgt
3061 cgaaactctc aaagtacgca gtcaggaacg tgcgcgacat gtcgtcggga aactcgcgcg
3121 gaaacatgtt gttgtaaccg aacgggtccc atagcgccaa aaccaaatct gccagcgtca
3181 atagaatgag cacgatgccg acaatggagc tggcttggat agcgattcga gttaacggcc
3241 ggccgctagt tggccacgta ataagtgtgc gttgaattta ttcgcaaaaa cattgcatat
3301 tttcggcaaa gtaaaatttt gttgcatacc ttatcaaaaa ataagtgctg catacttttt
3361 agagaaacca aataattttt tattgcatac ccgtttttaa taaaatacat tgcataccct
3421 cttttaataa aaaatattgc atactttgac gaaacaaatt ttcgttgcat acccaataaa
3481 agattattat attgcatacc cgtttttaat aaaatacatt gcataccctc ttttaataaa
3541 raatattgca tacgttgacg aaacaaattt tcgttgcata cccaataaaa gattattata
3601 ttgcatacct tttcttgcca taccatttag ccgatcaatt gtgctcggca acagtatatt
3661 tgtggtgtgc caaccaacaa ctctagtgct gcatccaacg cgttgggagc tctccggatc
3721 aattcggctt caggtaccgt cgacgatgta ggtcacggtc tcgaagccgc ggtgcgggtg
3781 ccagggcgtg cccttgggct ccccgggcgc gtactccacc tcacccatct ggtccatcat
3841 gatgaacggg tcgaggtggc ggtagttgat cccggcgaac gcgcggcgca ccgggaagcc
3901 ctcgccctcg aaaccgctgg gcgcggtggt cacggtgagc acgggacgtg cgacggcgtc
3961 ggcgggtgcg gatacgcggg gcagcgtcag cgggttctcg acggtcacgg cgggcatgtc
4021 gacaagccga attgatccac tagaaggcct aattcgcggc cgcggatctg atcccgggcg
4081 ggtaccgaat tctagtatgt atgtaagtta ataaaaccct tttttggaga atgtagattt
4141 aaaaaaacat attttttttt tattttttac tgcactggat atcattgaac ttatctgatc
4201 agttttaaat ttacttcgat ccaagggtat ttgaagtacc aggttctttc gattacctct
4261 cactcaaaat gacattccac tcaaagtcag cgctgtttgc ctccttctct gtccacagaa
4321 atatcgccgt ctctttcgcc gctgcgtccg ctatctcttt cgccaccgtt tgtagcgtta
4381 cctagcgtca atgtccgcct tcagttgcac tttgtcagcg gtttcgtgac gaagctccaa
4441 gcggtttacg ccatcaatta aacacaaagt gctgtgccaa aactcctctc gcttcttatt
4501 tttgtttgtt ttttgagtga ttggggtggt gattggtttt gggtgggtaa gcaggggaaa
4561 gtgtgaaaaa tcccggcaat gggccaagag gatcaggagc tattaattcg cggaggcagc

4621 aaacacccat ctgccgagca tctgaacaat gtgagtagta catgtgcata catcttaagt
4681 tcacttgatc tataggaact gcgattgcaa catcaaattg tctgcggcgt gagaactgcg
4741 acccacaaaa atcccaaacc gcaatcgcac aaacaaatag tgacacgaaa cagattattc
4801 tggtagctgt gctcgctata taagacaatt tttaagatca tatcatgatc aagacatcta
4861 aaggcattca ttttcgacta cattcttttt tacaaaaaat ataacaacca gatattttaa
4921 gctgatccta gatgcacaaa aaataaataa aagtataaac ctacttcgta ggatacttcg
4981 ttttgttcgg ggttagatga gcataacgct tgtagttgat atttgagatc ccctatcatt
5041 gcagggtgac agcggacgct tcgcagagct gcattaacca gggcttcggg caggccaaaa
5101 actacggcac gctcctgcca cccagtccgc cggaggactc cggttcaggg agcggccaac
5161 tagccgagaa cctcacctat gcctggcaca atatggacat ctttggggcg gtcaatcagc
5221 cgggctccgg atggcggcag ctggtcaacc ggacacgcgg actattctgc aacgagcgac
5281 acataccggc gcccaggaaa catttgctca agaacggtga gtttctattc gcagtcggct
5341 gatctgtgtg aaatcttaat aaagggtcca attaccaatt tgaaactcag tttgcggcgt
5401 ggcctatccg ggcgaacttt tggccgtgat gggcagttcc ggtgccggaa agacgaccct
5461 gctgaatgcc cttgcctttc gatcgccgca gggcatccaa gtatcgccat ccgggatgcg
5521 actgctcaat ggccaacctg tggacgccaa ggagatgcag gccaggtgcg cctatgtcca
5581 gcaggatgac ctctttatcg gctccctaac ggccagggaa cacctgattt tccaggccat
5641 ggtgcggatg cccacgacat tgacctatcg gcagcgagtg gcccgcgtgg atcaggtgat
5701 ccaggagctt tcgctcagca aatgtcagca cacgatcatc ggtgtgcccg gcagggtgaa
5761 aggtctgtcc ggcggagaaa ggaagcgtct ggcattcgcc tccgaggcac taaccgatcc
5821 gccgcttctg atctgcgatg agcccacctc cggactggac tcatttaccg cccacagcgt
5881 cgtccaggtg ctgaagaagc tgtcgcagaa gggcaagacc gtcatcctga ccattcatca
5941 gccgtcttcc gagctgtttg agctctttga caagatcctt ctgatggccg agggcagggt
6001 agctttcttg ggcactccca gcgaagccgt cgacttcttt tcctagtgag ttcgatgtgt
6061 ttattaaggg tatctagcat tacattacat ctcaactcct atccagcgtg ggtgcccagt
6121 gtcctaccaa ctacaatccg gcggactttt acgtacaggt gttggccgtt gtgcccggac
6181 gggagatcga gtcccgtgat cggatcgcca agatatggcg acaattttgc tattagcaaa
6241 gtagcccggg atatggagca gttgttggcc accaaaaatt tggagaagcc actggagcag
6301 ccggagaatg ggtacaccta caaggccacc tggttcatgc agttccgggc ggtcctgtgg
6361 cgatcctggc tgtcggtgct caaggaacca ctcctcgtaa agtgcgact tattcagaca
6421 acggtgagtg gttccagtgg aaacaaatga tataacgctt acaattcttg gaaacaaatt
6481 cgctagattt tagttagaat tgcctgattc cacacccttc ttagtttttt tcaatgagat
6541 gtatagttta tagttttgca gaaaataaat aaatttcatt taactcgcga acatgttgaa
6601 gatatgaata ttaatgagat gcgagtaaca ttttaatttg cagatggttg ccatcttgat
6661 tggcctcatc tttttgggcc aacaactcac gcaagtgggc gtgatgaata tcaacggagc
6721 catcttcctc ttcctgacca acatgacctt tcaaaacgtc tttgccacga taaatgtaag
6781 tcttgtttag aatacatttg catattaata atttactaac tttctaatga atcgattcga
6841 tttaggtgtt cacctcagag ctgccagttt ttatgaggga ggcccgaagt cgactttatc
6901 gctgtgacac atactttctg ggcaaaacga ttgccgaatt accgcttttt ctcacagtgc
6961 cactggtctt cacggcgatt gcctatccga tgatcggact gcgggccgga gtgctgcact
7021 tcttcaactg cctggcgctg gtcactctgg tggccaatgt gtcaacgtcc ttcggatatc
7081 taatatcctg cgccagctcc tcgacctcga tggcgctgtc tgtgggtccg ccggttatca
7141 taccattcct gctctttggc ggcttcttct tgaactcggg ctcggtgcca gtatacctca
7201 aatggttgtc gtacctctca tggttccgtt acgccaacga gggtctgctg attaaccaat
7261 gggcggacgt ggagccgggc gaaattagct gcacatcgtc gaacaccacg tgccccagtt
7321 cgggcaaggt catcctggag acgcttaact tctccgccgc cgatctgccg ctggactacg
7381 tgggtctggc cattctcatc gtgagcttcc gggtgctcgc atatctggct ctaagacttc
7441 gggcccgacg caaggagtag ccgacatata tccgaaataa ctgcttgttt tttttttac
7501 cattattacc atcgtgttta ctgtttattg ccccctcaaa aagctaatgt aattatattt
7561 gtgccaataa aaacaagata tgacctatag aatacaagta tttccccttc gaacatcccc
7621 acaagtagac tttggatttg tcttctaacc aaaagactta cacacctgca taccttacat
7681 caaaaactcg tttatcgcta cataaaacac cgggatatat tttttatata catacttttc
7741 aaatcgcgcg ccctcttcat aattcacctc caccacacca cgtttcgtag ttgctctttc
7801 gctgtctccc acccgctctc cgcaacacat tcaccttttg ttcgacgacc ttggagcgac

7861 tgtcgttagt tccgcgcgat tcggttcgct caaatggttc cgagtggttc atttcgtctc
7921 aatagaaatt agtaataaat atttgtatgt acaatttatt tgctccaata tatttgtata
7981 tatttccctc acagctatat ttattctaat ttaatattat gactttttaa ggtaattttt
8041 tgtgacctgt tcggagtgat tagcgttaca atttgaactg aaagtgacat ccagtgtttg
8101 ttccttgtgt agatgcatct caaaaaaatg gtgggcataa tagtgttgtt tatatatatc
8161 aaaaataaca actataataa taagaataca tttaatttag aaaatgcttg gatttcactg
8221 gaactagaat taattcggct gctgctctaa acgacgcatt tcgtactcca aagtacgaat
8281 tttttccctc aagctcttat tttcattaaa caatgaacag gacctaacgc acagtcacgt
8341 tattgtttac ataaatgatt tttttacta ttcaaactta ctctgtttgt gtactcccac
8401 tggtatagcc ttcttttatc ttttctggtt caggctctat cactttacta ggtacggcat
8461 ctgcgttgag tcgcctcctt ttaaatgtct gacctttgc aggtgcagcc ttccactgcg
8521 aatctttaaa gtgggtatca caaatttggg agttttcacc aaggctgcac ccaaggctct
8581 gctcccacaa ttttctctta atagcacact tcggcacgtg aattaatttt actccagtca
8641 cagctttgca gcaaaatttg caatatttca ttttttttta ttccacgtaa gggttaatgt
8701 tttcaaaaaa aaattcgtcc gcacacaacc tttcctctca acaagcaaac gtgcactgaa
8761 tttaagtgta tacttcggta agcttcggct atcgacggga ccaccttatg ttatttcatc
8821 atgggccaga cccacgtagt ccagcggcag atcggcggcg gagaagttaa gcgtctccag
8881 gatgaccttg cccgaactgg ggcacgtggt gttcgacgat gtgcagctaa tttcgcccgg
8941 ctccacgtcc gcccattggt taatcagcag accctcgttg gcgtaacgga accatgagag
9001 gtacgacaac catttgaggt atactggcac cgagcccgag ttcaagaaga aggcgttttt
9061 ccataggctc cgcccccctg acgagcatca caaaaatcga cgctcaagtc agaggtggcg
9121 aaacccgaca ggactataaa gataccaggc gtttccccct ggaagctccc tcgtgcgctc
9181 tcctgttccg accctgccgc ttaccggata cctgtccgcc tttctccctt cgggaagcgt
9241 ggcgctttct caatgctcac gctgtaggta tctcagttcg gtgtaggtcg ttcgctccaa
9301 gctgggctgt gtgcacgaac cccccgttca gcccgaccgc tgcgccttat ccggtaacta
9361 tcgtcttgag tccaacccgg taagacacga cttatcgcca ctggcagcag ccactggtaa
9421 caggattagc agagcgaggt atgtaggcgg tgctacagag ttcttgaagt ggtggcctaa
9481 ctacggctac actagaagga cagtatttgg tatctgcgct ctgctgaagc cagttacctt
9541 cggaaaaaga gttggtagct cttgatccgg caaacaaacc accgctggta gcggtggttt
9601 ttttgtttgc aagcagcaga ttacgcgcag aaaaaaagga tctcaagaag atcctttgat
9661 cttttctacg gggtctgacg ctcagtggaa cgaaaactca cgttaaggga ttttggtcat
9721 gagattatca aaaaggatct tcacctagat ccttttaaat taaaaatgaa gttttaaatc
9781 aatctaaagt atatatgagt aaacttggtc tgacagttac caatgcttaa tcagtgaggc
9841 acctatctca gcgatctgtc tatttcgttc atccatagtt gcctgactcc ccgtcgtgta
9901 gataactacg atacgggagg gcttaccatc tggccccagt gctgcaatga taccgcgaga
9961 cccacgctca ccggctccag atttatcagc aataaaccag ccagccggaa gggccgagcg
10021 cagaagtggt cctgcaactt tatccgcctc catccagtct attaattgtt gccgggaagc
10081 tagagtaagt agttcgccag ttaatagttt gcgcaacgtt gttgccattg ctacaggcat
10141 cgtggtgtca cgctcgtcgt ttggtatggc ttcattcagc tccggttccc aacgatcaag
10201 gcgagttaca tgatccccca tgttgtgcaa aaaagcggtt agctccttcg gtcctccgat
10261 cgttgtcaga agtaagttgg ccgcagtgtt atcactcatg gttatggcag cactgcataa
10321 ttctcttact gtcatgccat ccgtaagatg cttttctgtg actggtgagt actcaaccaa
10381 gtcattctga gaatagtgta tgcggcgacc gagttgctct tgcccggcgt caatacggga
10441 taataccgcg ccacatagca gaactttaaa agtgctcatc attggaaaac gttcttcggg
10501 gcgaaaactc tcaaggatct taccgctgtt gagatccagt tcgatgtaac ccactcgtgc
10561 acccaactga tcttcagcat cttttacttt caccagcgtt tctgggtgag caaaaacagg
10621 aaggcaaaat gccgcaaaaa agggaataag ggcgacacgg aaatgttgaa tactcatact
10681 cttccttttt caatattatt gaagcattta tcagggttat tgtctcatga gcggatacat
10741 atttgaatgt atttagaaaa ataaacaaat aggggttccg cgcacatttc cccgaaaagt
10801 gccacctgac gtctaagaaa ccattattat catgacatta acctataaaa ataggcgtat
10861 cacgaggccc tttcgtctcg cgcgtttcgg tgatgacggt gaaaacctct gacacatgca
10921 gctcccggag acggtcacag cttgtctgta agcggatgcc gggagcagac aagcccgtca
10981 gggcgcgtca gcgggtgttg gcgggtgtcg gggctggctt aactatgcgg catcagagca
11041 gattgtactg agagtgcacc atatgcggtg tgaaataccg caccgaatcg cgcggaacta

```
11101 acgacagtcg ctccaaggtc gtcgaacaaa aggtgaatgt gttgcggaga gcgggtggga
11161 gacagcgaaa gagcaactac gaaacgtggt gtggtggagg tgaattatga agagggcgcg
11221 cgatttgaaa agtatgtata taaaaaatat atcccggtgt tttatgtagc gataaacgag
11281 tttttgatgt aaggtatgca ggtgtgtaag tcttttggtt agaagacaaa tccaaagtct
11341 acttgtgggg atgttcgaag gggaaatact tgtattctat aggtcatatc ttgtttttat
11401 tggcacaaat ataattacat tagctttttg agggggcaat aaacagtaaa cacgatggta
11461 ataatggtaa aaaaaaaaac aagcagttat ttcggatata tgtcggctac tccttgcgtc
11521 gggcccgaag tcttagagcc agatatgcga gcacccggaa gctcacgatg agaatggcca
11581 gaccatgatg aaataacata aggtggtccc gtcggcaaga gacatccact taacgtatgc
11641 ttgcaataag tgcgagtgaa aggaatagta ttctgagtgt cgtattgagt ctgagtgaga
11701 cagcgatatg attgttgatt aacccttagc atgtccgtgg ggtttgaatt aactcataat
11761 attaattaga cgaaattatt tttaaagttt tatttttaat aatttgcgag tacgca
//
```
