# Supplementary material for: Intestinal Stem Cells Exhibit Conditional Circadian Clock Function
Source: Stem Cell Reports. 2018 Nov 13;11(5):1287–301. doi: 10.1016/j.stemcr.2018.10.010 (PMC6235668; doi:10.1016/j.stemcr.2018.10.010)
Supplement: Document S2. Article plus Supplemental Information [file mmc2.pdf]

Stem Cell Reports
Article
OPEN ACCESS

# Intestinal Stem Cells Exhibit Conditional Circadian Clock Function

Kathyani Parasram,[1,3] Nathaniel Bernardon,[1,3] Maha Hammoud,[1] Hanna Chang,[1] Li He,[2] Norbert Perrimon,[2] and Phillip Karpowicz[1,*]
[1]Department of Biological Sciences, University of Windsor, Windsor, ON N9B 3P4, Canada
[2]Department of Genetics, Harvard Medical School, Boston, MA 02115, USA
[3]Co-first author
*Correspondence: phillip.karpowicz@uwindsor.ca
https://doi.org/10.1016/j.stemcr.2018.10.010

## SUMMARY

The circadian clock is a molecular pacemaker that produces 24-hr physiological cycles known as circadian rhythms. How the clock regulates stem cells is an emerging area of research with many outstanding questions. We tested clock function *in vivo* at the single cell resolution in the *Drosophila* intestine, a tissue that is exquisitely sensitive to environmental cues and has circadian rhythms in regeneration. Our results indicate that circadian clocks function in intestinal stem cells and enterocytes but are downregulated during enteroendocrine cell differentiation. *Drosophila* intestinal cells are principally synchronized by the photoperiod, but intestinal stem cell clocks are highly responsive to signaling pathways that comprise their niche, and we find that the Wnt and Hippo signaling pathways positively regulate stem cell circadian clock function. These data reveal that intestinal stem cell circadian rhythms are regulated by cellular signaling and provide insight as to how clocks may be altered during physiological changes such as regeneration and aging.

## INTRODUCTION

Circadian rhythms are 24-hr oscillations in animal physiology that are a product of the circadian clock. At its core, the circadian clock is composed of a conserved transcription/translation feedback system, whose activity throughout the cells of the body causes rhythms in their molecular functions (Hardin, 2011; Panda, 2016). Many animals contain a hierarchical circadian system, with clocks in brain neurons serving as a central pacemaker to transmit circadian timing to other peripheral tissues (Reppert and Weaver, 2002). In certain animals, such as insects and fish, most cells throughout the body can also directly transduce light/dark (photoperiod) cues to entrain their circadian clock timing (Hardin, 2011; Vatine et al., 2011). However, recent studies in *Drosophila* have shown that some elements of a hierarchical system are present and that signals propagated from the brain can drive rhythms in gene expression in distant organs (Xu et al., 2011). This suggests that inter-cellular signals that coordinate circadian timing throughout the animal body are conserved.

Transcriptomics has provided many insights into the genes that are regulated by the circadian clock, revealing that tissues have specific clock functions that can change under different physiological states (Tognini et al., 2017; Zhang et al., 2014). Most tissues are composed of a heterogeneous mixture of different cell types, and the role of the clock has been primarily studied at the tissue level. Fewer studies have analyzed specific cell populations within a single organ or tissue (Janich et al., 2011; Solanas et al., 2017). This is problematic, since readings would report signals from the average of all cells and obscure differences between different cell types or differences between cells of the same type. It is not clear whether all cells, including stem cells, in a single tissue contain circadian clocks, whether all cells of a specific cell type are homogeneous or heterogeneous in their clock functions, or whether changes occur under different physiological contexts. Although the imaging of cell cultures has provided information about clock function at the single-cell level (Nagoshi et al., 2004; Yeom et al., 2010), *in vitro* conditions contain a milieu of growth factors and cytokines that can affect circadian clock entrainment (Balsalobre et al., 2000). Hence, the synchrony and heterogeneity of circadian rhythms in tissue cells is not clear.

Another long-standing question is at what point the circadian clock arises during development (Agrawal et al., 2017; Brown, 2014; Umemura et al., 2017; Yagita et al., 2010). The clock is absent in mouse embryonic stem cells (Yagita et al., 2010) and only begins to function during embryonic differentiation (Umemura et al., 2017). In adult mice, circadian rhythms have been proposed to occur in certain populations of mouse hair follicle stem cells (Janich et al., 2011) and muscle stem cells (Solanas et al., 2017). *In vitro*, it was recently reported that mouse intestinal stem cells (ISCs) do not exhibit circadian rhythms and that clock function develops in differentiated cell types (Matsu-Ura et al., 2016). It is therefore not clear if tissue stem cells have circadian clock activity.

To answer these questions, we tested clock function *in vivo* at the single-cell resolution in the *Drosophila* intestine, a pseudo-stratified epithelium that contains a well-defined cell population. The *Drosophila* intestine contains a population of ISCs that, like those found in mammals, divide throughout life to produce all of the differentiated


This is an open access article under the CC BY license (http://creativecommons.org/licenses/by/4.0/).

epithelial cells of the intestine (Biteau et al., 2011). Previously, we showed that the circadian clock regulates regeneration timing in the *Drosophila* intestine and that circadian gene dysfunction in stem cells is deleterious, suggesting that ISCs have clock activity that is important for their function (Karpowicz et al., 2013). Like mammals, the *Drosophila* intestine contains ISCs that divide to give rise to enteroblasts (EBs), which differentiate into either absorptive enterocytes (ECs) or nutrient-/pathogen-sensing enteroendocrine cells (EEs) that convey information about the intestinal environment to the body (Beebe et al., 2015; Park et al., 2016; Song et al., 2014). *Drosophila* ISCs are an undifferentiated population of cells in the intestinal epithelium, whose progeny terminally differentiate into tissue-specific cells. Because circadian rhythms are proposed to play a critical role in stem cell biology (Brown, 2014), we used this system to answer questions surrounding circadian clock activity in stem cells and their surrounding tissue cells.

Our data reveal that clocks are present in ISCs, EBs, and ECs, but not in EEs, showing that clock function does not necessarily correlate to cellular differentiation status. Circadian clocks in *Drosophila* intestinal cells are subject to signaling cues, including the timing of food intake. During intestinal stress, ISC clock function is dependent on surrounding cells, and the Notch (N), Wnt, and Hippo signaling pathways, important regulators of the ISC niche, also regulate circadian clock function in ISCs. These results shed light on how tissue stem cell clock rhythms are integrated with the surrounding tissue cells and how physiological changes during regeneration and aging can alter these rhythms.

## RESULTS

### Circadian Clock Activity Is Heterogeneous in the Intestine

The *Drosophila* circadian clock regulates gene expression and comprises the transactivators *CLK/CYC* and their targets and negative repressors *PER/TIM* (Figure 1A). To visualize clock activity in the *Drosophila* intestine, we constructed two clock reporters: (1) *Clock$^{PER}$* containing 123 bp of the PER promoter (Hao et al., 1997); (2) *Clock$^{TIM}$* containing 174 bp of the TIM promoter (McDonald et al., 2001), both arranged in a 4× tandem series upstream from a nuclear localization signal/superfolder destabilized GFP (Figures 1B and S2A). To quantify circadian transcription of this reporter in the intestine, we synchronized *Drosophila* carrying these reporters to 12-hr light/12-hr dark (LD) for 5 days, then tested gene expression in the intestine of controls versus clock-dead *cyc$^{01}$* null mutants by RT-qPCR. *GFP* RNA expression from both reporters was rhythmic, in phase with both *PER* and *TIM* (peak is zeitgeber time [ZT]15–18), and was *CYC* dependent (Figures 1E and S1). The expression of *PER* and *TIM* in the intestine is consistent with our previous results for clock gene transcription (Karpowicz et al., 2013). These data demonstrate that transcription of *GFP* from the reporter construct recapitulates endogenous CLK/CYC transcriptional activity in the intestine.

Intestines were examined visually: *Clock$^{PER}$* and *Clock$^{TIM}$* intestines both express reporter GFP, but it is strongest in the anterior regions and the posterior regions (Figure 1C). We collected n $\geq$ 10 individual intestines from synchronized *Drosophila*, imaged them using a fluorescent slide scanner, and quantified GFP signal from tiled whole-tissue scans containing z stacks of all of the cells present throughout the entire epithelium (see Experimental Procedures for details). Under LD conditions, both reporters showed rhythms in the whole intestine that were *CYC* dependent, consistent with their RNA expression (Figures 1D, 1F, and S2B), and the peak of *GFP* RNA expression preceded its signal by approximately 6 hr (ZT18 and ZT0, respectively). To confirm that these fluorescence rhythms were circadian in nature, *Drosophila* containing *Clock$^{PER}$* were shifted to complete dark photoperiod (DD), immediately following LD synchronization, and intestines were collected for an additional 24 hr. GFP rhythms persisted under these conditions in controls but not in *cyc$^{01}$* mutants, indicating that the *Clock$^{PER}$* reporter accurately reflects free-running *CYC*-dependent circadian clock activity in the *Drosophila* intestine (Figure 1F).

The *Drosophila* intestine is subdivided into >10 different anterior to posterior sub-regions based on cell composition, morphology, and gene expression (Buchon et al., 2013). Since GFP signal throughout the intestine is heterogeneous (Figure 1C), we asked whether clock activity varied throughout this tissue. To test this, the R2 (anterior) versus R5 (posterior) regions were quantified separately from the same tissue samples. No differences in circadian clock phase were noted between the regions (Figure 2A); both essentially followed the same timed rhythms as the whole tissue (Figure 1F). This suggests that, despite dissimilar physiological functions, circadian clocks in the anterior to posterior intestine of *Drosophila* are synchronously timed.

Genome-wide analysis has previously indicated that ISCs, EBs, EEs, and ECs in the *Drosophila* intestine (Figure 2B) express clock genes at some level (Figure S3A) (Dutta et al., 2015). However, circadian clock function cannot be assumed based on these data, because circadian gene expression must be tested at a specific time of day to determine rhythms in transcript expression. We tested circadian activity in all four cell types at CT0 (the peak of *Clock$^{PER}$* signal), when intestinal epithelial cells are GFP+,

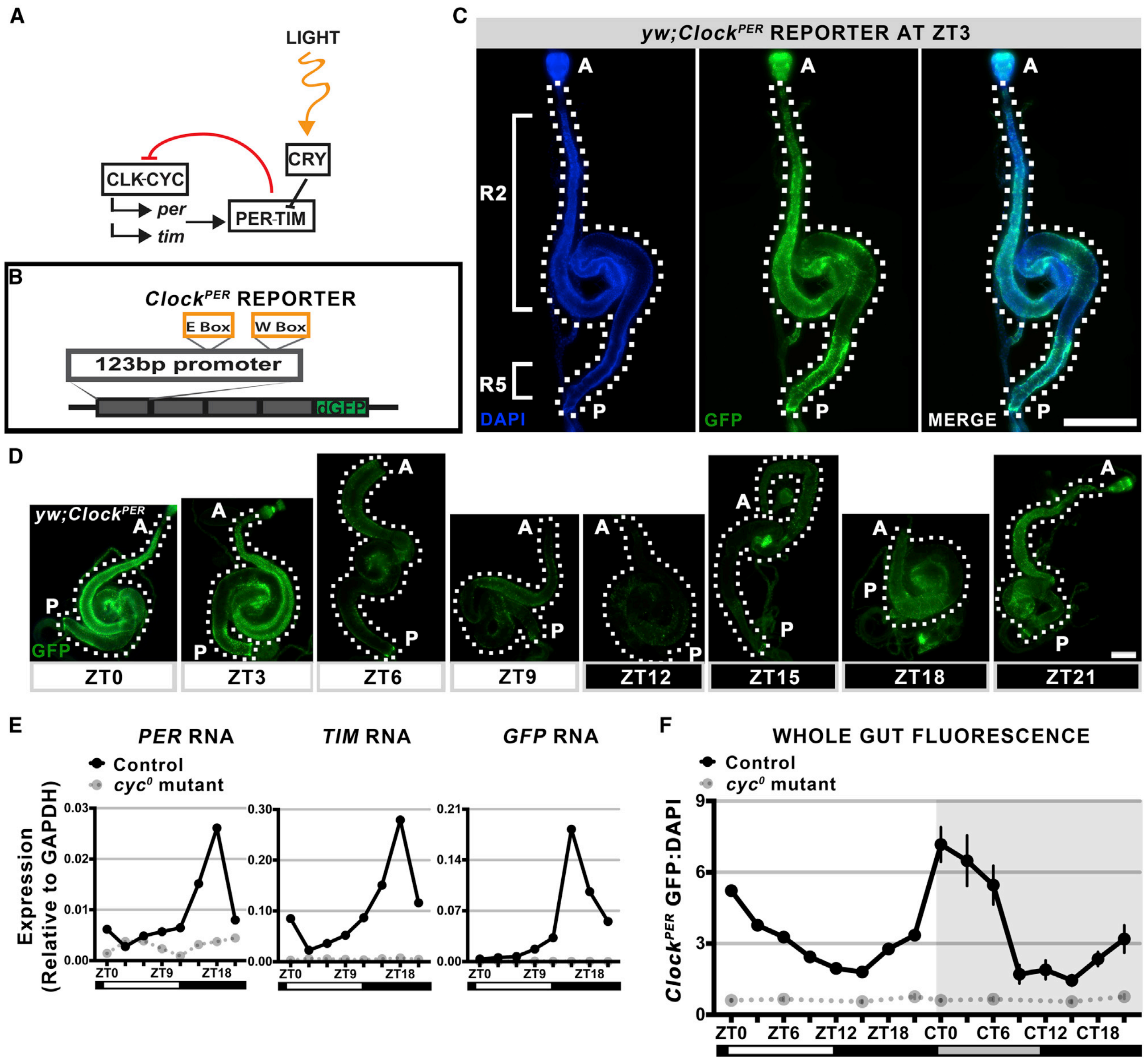


**Figure 1. The $Clock^{PER}$ Reporter Measures Intestinal Circadian Clock Activity**

(A) Schematic of the circadian clock in *Drosophila* showing that CLK/CYC promotes transcription, and PER/TIM repress this activity. Light acts on CRY degrading TIM to entrain the *Drosophila* clock to photoperiod.

(B) Schematic of the $Clock^{PER}$ reporter where 123bp *PER* promoter drives expression of destabilized GFP (dGFP) to report temporal changes in clock activity.

(C) Representative image of a *Drosophila* intestine at ZT3 showing $Clock^{PER}$ (GFP) in the anterior (R2) region and in the posterior (R5) region. DAPI counterstains nuclei. A, the anterior region; P, posterior. Scale bar represents 500 μm.

(D) Representative images of $Clock^{PER}$ (GFP) over a 24-hr timeline under LD photoperiod shows 24-hr changes in expression with a peak at ZT0. A, anterior region; P, posterior. Scale bar represents 500 μm.

(E) RT-qPCR expression of entire $Clock^{PER}$ intestine for *GFP*, *PER*, and *TIM* shows that these have similar expression phases, hence the $Clock^{PER}$ reporter reports endogenous CLK/CYC transcriptional activity. Each data point represents a signal obtained from n = 10 intestines. Results of additional qPCR experiments are shown in Figure S1.

(F) Graph of $Clock^{PER}$ GFP signal normalized to DAPI under LD photoperiod, followed by 24 hr in DD for the entire intestine. Circadian rhythms of GFP are present, and the $cyc^{0}$ mutant has no circadian transactivation and is thus negative at all times.

Data presented as mean of $n \geq 10$ intestines, error bars show ±SEM (two-way ANOVA $F = 9.552$, $p < 0.0001$). See also Figures S1 and S2.

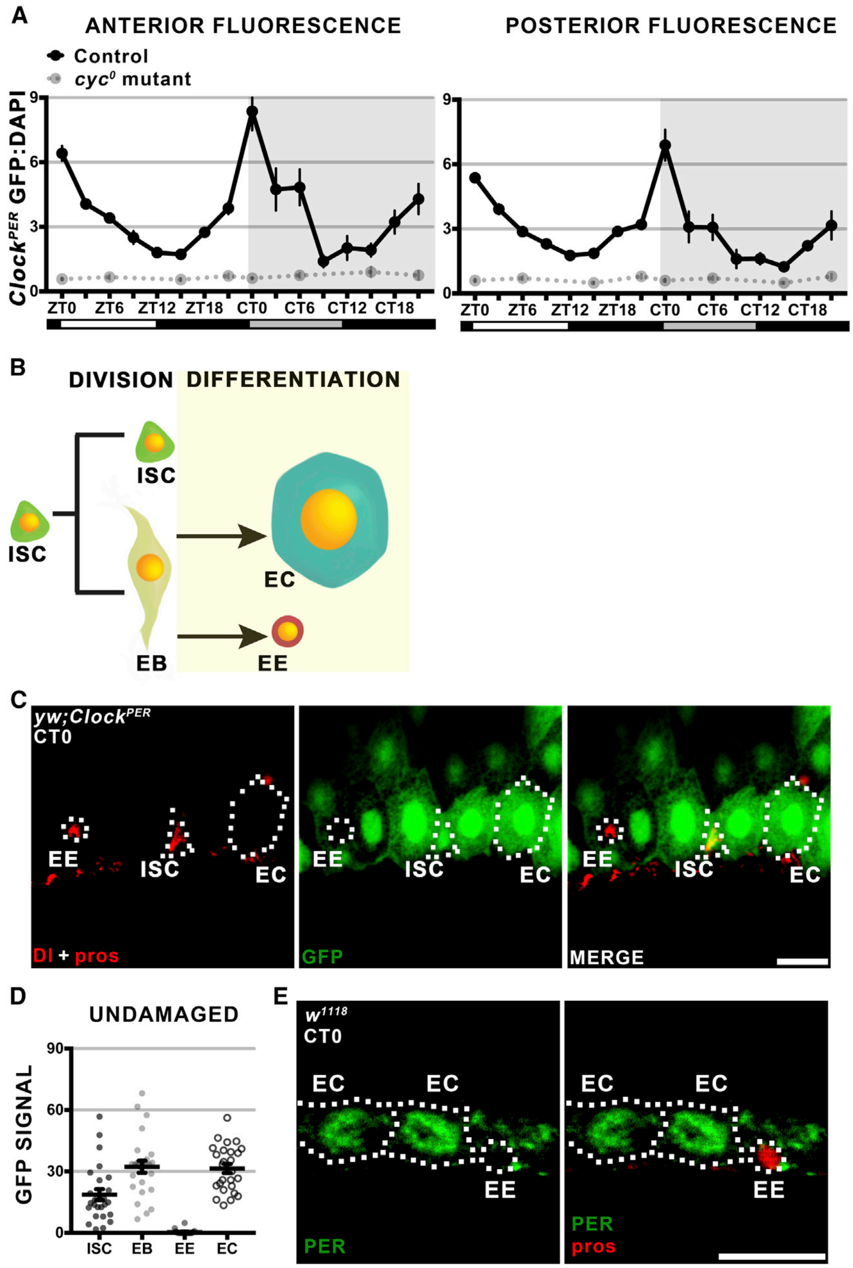


**Figure 2. The $Clock^{PER}$ Reporter Is Not Expressed in All Intestinal Epithelial Cells**

(A) Graphs of $Clock^{PER}$ GFP signal normalized to DAPI under LD photoperiod followed by 24 hr in DD for anterior (left) and posterior (right). Both regions display similar circadian rhythms and the $cyc^{0}$ mutant shows no circadian transactivation. Data presented as mean of n $\geq$ 10 intestines. Error bars show ±SEM. Control versus $cyc^{0}$: anterior (two-way ANOVA F = 5.842, p < 0.0001), posterior (two-way ANOVA F = 10.7, p < 0.0001).

(B) Schematic of the differentiation of Delta (Dl) positive ISCs, which self-renew and produce differentiated Dl-negative EBs, progenitor cells that differentiate into absorptive ECs or prospero (pros)-positive EE.

(C) Representative confocal z stack showing $Clock^{PER}$ GFP signal in the epithelium. Cells of interest are outlined: Dl+ marks ISCs, and pros+ marks EEs; ECs are the large polyploid cells. Scale bar represents 10 μm.

(D) Quantification of GFP intensity from confocal sections shows clock activity is absent in EEs, but present in the other three epithelial cell types (one-way ANOVA F = 42.49, p < 0.0001). Data show n > 25 cells from each cell type, error bars show ±SEM.

(E) Confocal section showing nuclear PER antibody staining in ECs but not EEs. Cells of interest are outlined: pros+ marks EEs, ECs are the large polyploid cells. Scale bar represents 10 μm.

See also Figure S3.

indicative of CLK/CYC transcriptional activity (Figure 2C). We quantified the fluorescence signal of single confocal sections through the middle of each cell, revealing that all ISCs, EBs, and ECs express the $Clock^{PER}$ reporter signal but EEs do not exhibit reporter activity (Figure 2D). The unexpected finding that EEs do not have clock activity was further tested using the $Clock^{TIM}$ reporter, this time inserted on a different chromosome to avoid possible EE-specific *PER* promoter silencing and/or chromatin silencing effects. EEs do not express GFP from the $Clock^{TIM}$ reporter either (Figures S3C–S3E). We further asked if EEs might have clock activity out of phase with the other cell types, by testing them at different time points than CT0, but found no signal at any time (Figure S3F). Finally, PER protein was tested by antibody staining, which we have previously shown to be nuclear in ISC and EC cells (Karpowicz et al., 2013). The majority of EEs examined have very low PER signal, especially in the nucleus, compared with EC cells where PER nuclear staining is strong (Figures 2E and S3G). Overall these data suggest that clock activity is present in undifferentiated stem cells and EBs, as well as differentiated ECs, but is turned off during EE differentiation.

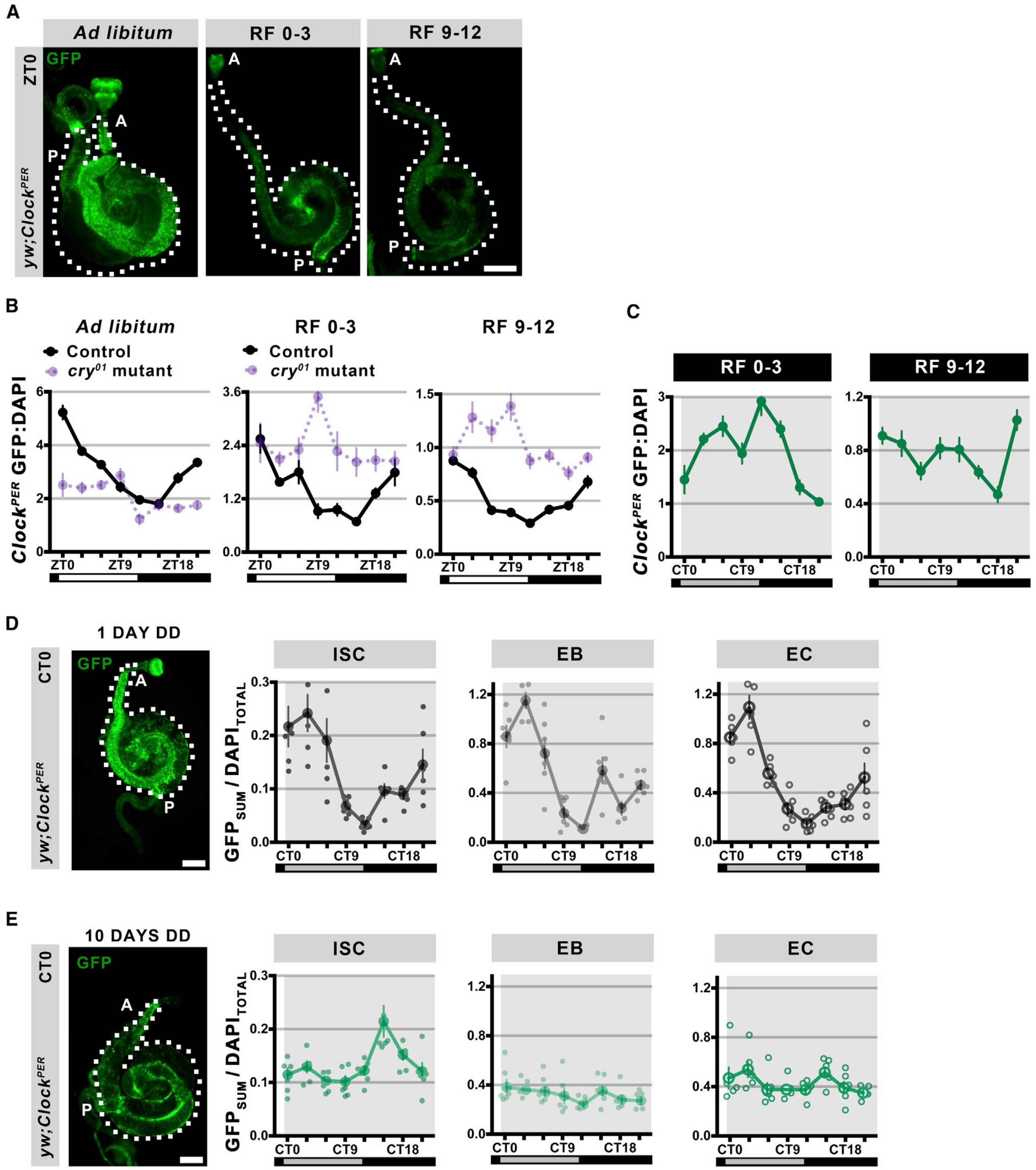


**Figure 3. Feeding Time Can Entrain the *Drosophila* Intestinal Clock**

(A) Representative images of *Clock*$^{PER}$ intestines from flies fed *ad libitum* or restricted to ZT0-3 or ZT9-12. GFP+ signal is lower under restricted feeding. A, the anterior region; P, posterior. Scale bar represents 250 μm.

(B) Graphs of *Clock*$^{PER}$ GFP:DAPI signal in the whole intestine under LD photoperiod under *ad libitum* or restricted feeding. Control data (left graph) are the same as used in Figure 1F. *Ad libitum*-fed *CRY* mutants (*cry*$^{01}$) show higher GFP levels during the daytime (ZT0-9) than night (ZT12-21), and are significantly different than controls (two-way ANOVA $F = 13.15$, $p < 0.0001$). Under restricted feeding, control

*(legend continued on next page)*

Clock function does not correlate with increased differentiation in this tissue, and *Drosophila* ISCs exhibit clock activity consistent with previous studies of skin and muscle stem cells in mammals (Janich et al., 2011; Solanas et al., 2017).

### Feeding Can Regulate the *Drosophila* Intestinal Clock

In mammals, signals from the brain synchronize circadian timing throughout the body (Reppert and Weaver, 2002), but restricting feeding time can synchronize peripheral tissues such as the liver directly (Damiola et al., 2000). Unlike mammals, *Drosophila* cells are themselves directly responsive to photoperiod (Hardin, 2011), yet recent studies have shown that feeding time can entrain the fat body (the insect equivalent of the liver) directly (Xu et al., 2011). To test whether the intestine is entrainable by feeding time, we used the $Clock^{PER}$ reporter in the $cry^{01}$ mutant background. In *Drosophila*, *CRY* transduces photoperiod entrainment of the clock by targeting *TIM* for degradation (Figure 1A) (Emery et al., 1998), and CRY+ cells are present in the *Drosophila* intestine (Agrawal et al., 2017). Hence we predicted that the $cry^{01}$ mutant would reveal circadian synchronizing cues that might be normally obscured by the photoperiod. Under LD *ad libitum* feeding conditions, control intestines show a rhythm that peaks at ZT0; *CRY* mutants do not show this rhythm but instead have slightly elevated CLK/CYC activity during the light phase (ZT0-9) (Figures 3A and 3B), perhaps reflecting the time when flies are most active and feeding. Restricting feeding to ZT0–3, the peak of maximal food consumption (Xu et al., 2008), or ZT9–12 in LD conditions, does not affect wild-type intestine rhythms. These maintain the same phase circadian rhythms under restricted feeding as *ad libitum* feeding, although with a lower amplitude, likely due to decreased overall food consumption (Figures 3A and 3B). This indicates that photoperiod is a dominant entraining factor in the *Drosophila* intestine, consistent with the dominant effect of photoperiod in regulating activity rhythms (Oishi et al., 2004). However, $cry^{01}$ mutant intestines exhibit peaks in CLK/CYC activity, with a peak at ZT9 if fed at ZT0–3, and a peak at ZT3–9 if fed at ZT9–12 (Figures 3A and 3B). Although feeding time did not produce a consistent CLK/CYC activity time (i.e., the reporter peaks 6 hr after ZT0–3 feeding but peaks 15 hr after ZT9–12 feeding), these results suggest that food intake can regulate CLK/CYC transactivation when photoperiod cues are not transduced through *CRY*.

We further tested the ability of restricted feeding to entrain intestinal clocks by maintaining wild-type $Clock^{PER}$ *Drosophila* for 5 days in the absence of photoperiod under a restricted feeding regimen. In peripheral tissues, it has been noted that circadian clock activity dampens in individuals maintained under free-running conditions for >2 days (Ivanchenko et al., 2001; Stanewsky et al., 1997), hence we predicted that two different restricted feedings would entrain two different $Clock^{PER}$ rhythms. When fed at CT0–3, reporter activity has a double-peak distribution with a maximum at CT12 (Figure 3D). Feeding at CT9–12 also produces a double-peak distribution but with a maximum at CT21 (Figure 3D). Thus, in both cases, feeding preceded a peak of CLK/CYC activity by 9 hr, suggesting that food intake can regulate circadian clock function in the wild-type intestine when photoperiod is absent. We next tested the importance of photoperiod on synchronizing intestinal cell clocks, by comparing *Drosophila* exposed to different duration of DD free-running conditions. One day following shift from LD to DD, intestinal cells maintain synchronous circadian rhythms, with ISCs, EBs, and ECs in phase (Figure 3D). However, at 10 days DD free running, intestinal cells lose their rhythmicity and ISCs, EBs, and ECs

intestines follow similar rhythms (albeit with different amplitude), with the same peaks and troughs irrespective of feeding regimen, suggesting that photoperiod is the key entrainment factor in the intestine. *CRY* mutants show distinct rhythms in these different regimens. $cry^{01}$ are significant in RF0-3 (one-way ANOVA F = 2.417, p = 0.0270) and RF9-12 (one-way ANOVA F = 6.083, p < 0.0001); control versus $cry^{01}$ is significant: RF0-3 (two-way ANOVA F = 5.092, p < 0.0001), RF9-12 (two-way ANOVA F = 9.63, p < 0.0001). Data presented as mean of n $\geq$ 10 intestines, error bars show ±SEM.

(C) Graphs of $Clock^{PER}$ GFP:DAPI signal in wild-type flies with 5 days of restricted feeding at CT0-3 versus CT9-12 (DD conditions). Alterations in clock reporter activity suggest the timing of feeding affects clock function in these otherwise free-running conditions. Data presented as mean of n $\geq$ 10 intestines, error bars show ±SEM. One-way ANOVA: control 0–3 (F = 12.48, p < 0.0001); control 9–12 (F = 2.161, p = 0.0467).

(D) Representative image of a $Clock^{PER}$ GFP+ *Drosophila* intestine at CT0, at 1 day DD. DAPI counterstains nuclei. A, the anterior region; P, posterior. Scale bar represents 250 μm. Graphs show quantification of cell-specific rhythms in the posterior (R5) region with EC, EB, and ISC signals analyzed separately. One day after LD photoperiod, all clock active intestinal cells exhibit synchronous circadian rhythms. $GFP_{SUM}$ is fluorescence from n = 5 cells of each cell type (ISC, EB, or EC) normalized to the DAPI coming from all cells quantified (n = 15 total). Data are the mean of n = 6 intestines, error bars show ±SEM. One-way ANOVA: ISC (F = 8.485, p < 0.0001), EB (F = 21.36, p < 0.0001), EC (F = 26.37, p < 0.0001).

(E) The same analysis carried out 10 days after DD. Following a long period of free-running conditions, intestinal cells are no longer synchronous and the average rhythm for each cell type is altered. One-way ANOVA: ISC (F = 6.283, p < 0.0001), EB (F = 1.332, p = 0.2608), EC (F = 2.083, p = 0.0679).

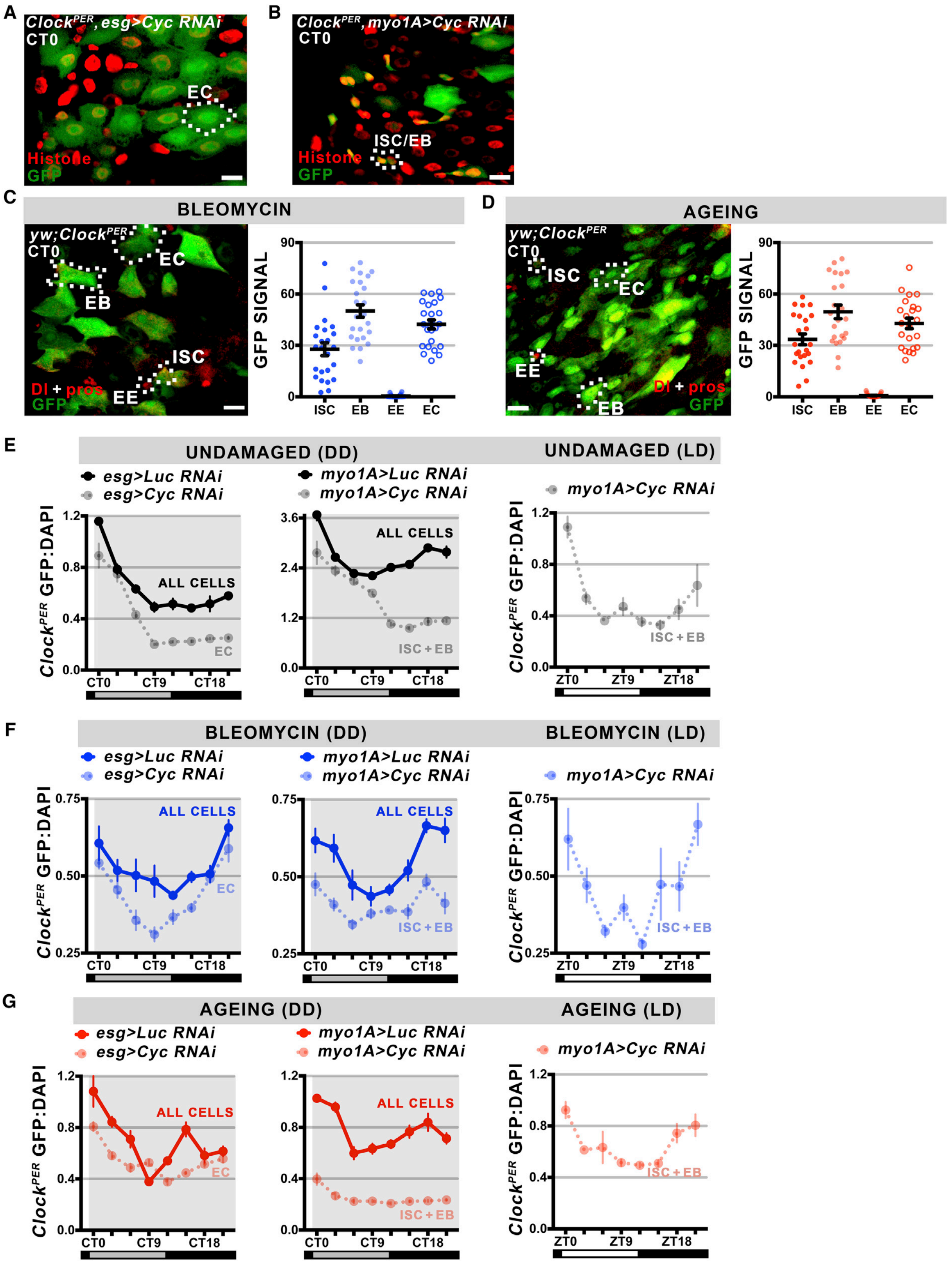


*(legend on next page)*

are either arrhythmic or out of phase (Figure 3E). These data suggest that intestinal cells drift out of synchrony if the individual loses long-term photoperiod cues, and behavior and physiological processes lose 24-hr rhythms. Taken together, our data show that photoperiod is a dominant synchronization cue for *Drosophila* intestinal clocks, but, in its absence, intestinal cell clocks can be regulated by timed food intake. This bears resemblance to the *Drosophila* fat body (Xu et al., 2011) and suggests that cell signaling processes may be able to modulate clock activity in the *Drosophila* intestine.

## Intestinal Stem Cell Clock Activity Is Non-autonomous

Previously, we reported that clock function is required in non-dividing differentiated ECs to generate circadian rhythms in stem cell proliferation (Karpowicz et al., 2013). This raised the question of whether a circadian communication system exists between the cells in the epithelium or whether cell-specific clocks function cell autonomously. To test this, we disrupted clock function in specific cell types: the *esg-Gal4* driver to disrupt *CYC* in undifferentiated precursors (ISCs + EBs) and the *Myo1A-Gal4* driver to disrupt *CYC* in differentiated ECs. Because clock activity in the intestinal epithelium is restricted to ISCs, EBs, and ECs (Figures 2C and 2D), these experiments test whether circadian rhythms are present in specific epithelial cell types when those surrounding them are absent. We entrained *Clock*$^{PER}$ flies carrying cell-specific *CYC* disruption constructs to LD, then released them into free-running DD conditions and analyzed tissue over 24 hr (Figures 4A and 4E). Loss of clock function in undifferentiated precursors (ISCs + EBs) did not disrupt circadian rhythms in differentiated ECs whose circadian timing persisted (Figure 4E), suggesting that EC clocks are not dependent on clocks in undifferentiated precursors. In contrast, loss of clock function in ECs alters the circadian rhythms of undifferentiated precursors (Figure 4E). This was apparent for two reasons. First, the number of precursors positive for clock activity after EC disruption was lower than the total number of precursors present in the intestine (Figure S3B), and which we previously found to be clock reporter active (Figure 2D). Second, the remaining clock-positive precursors continue to display a peak of clock activity at CT0, but CLK/CYC activity is prolonged (up to CT9) before returning to its baseline levels (Figure 4B; compare with Figure 3D). This suggests that PER/TIM repression is slightly delayed in the few remaining clock-live precursor cells when EC clocks are lost. We then tested whether restoring photoperiod could rescue clock function in ISC/EBs when the circadian clock was disrupted in ECs. Under LD photoperiod, *esg*>*CYC RNAi* ISC/EBs have normal clock function (Figure 4E), suggesting that indeed this is the case.

**Figure 4. Intestinal Stem Cell Clock Activity Is Regulated by Non-Cell-Autonomous Factors**

(A) Confocal z stack of *Clock*$^{PER}$ reporter in ISC/EB-specific *esg>CYC* knockdown. ECs, ISCs, and EBs are indicated, the smaller GFP+ cells are parts of differentiated ECs (that do not express *esg*) just outside the confocal stack.

(B) Confocal z stack of *Clock*$^{PER}$ reporter in EC-specific *myo1A>CYC* knockdown. ISC/EBs are indicated, the larger GFP+ cells with processes are most likely EBs differentiating into ECs but that do not yet express *myo1A*. Histone counterstains all nuclei present, scale bar represents 10 μm.

(C) Analysis of *Clock*$^{PER}$ GFP signal from confocal sections for bleomycin-treated flies. Scale bar represents 10 μm. ISCs, EBs, and ECs are GFP+ while EEs are GFP−. Data presented for >25 cells in each group, error bars show ±SEM (one-way ANOVA F = 56.34, $p < 0.0001$).

(D) Analysis of *Clock*$^{PER}$ GFP signal from confocal sections for aged flies. The distribution of cells during aging stress closely mirrors that observed during bleomycin-induced stress. Data presented for >25 cells in each group, error bars show ±SEM (one-way ANOVA F = 53.91, $p < 0.0001$).

(E) Left graph shows *Clock*$^{PER}$ GFP:DAPI signal for control (*Luc RNAi*) and *Cyc RNAi* knockdown in ISCs and EBs (*esg*) under DD conditions. ECs are not affected by the knockdown of *CYC* in the surrounding precursors (left) and express similar clock activity (see Figure 3D). *esg>Luc RNAi* versus *Cyc RNAi* (two-way ANOVA F = 2.701, p = 0.0115). Center graph shows *Clock*$^{PER}$ GFP:DAPI signal for control (*Luc RNAi*) and *Cyc RNAi* knockdown in ECs (*myo1A*) under DD conditions. When *Cyc* is knocked down in ECs (right), *Clock*$^{PER}$ GFP rhythms in control are higher and phase advanced compared with *Cyc RNAi* knockdown in ECs, and compared with their normal rhythms (see Figure 3D). *myo1A>Luc RNAi* versus *Cyc RNAi* (two-way ANOVA F = 20.15, $p < 0.0001$). Right graph shows *Clock*$^{PER}$ GFP:DAPI signal for myo1A > *Cyc RNAi* under normal 12:12 LD photoperiod. Under LD, ISC/EB rhythm (one-way ANOVA F = 9.577, $p < 0.0001$) is normal (compare with Figure 3D).

(F) Analysis of *Clock*$^{PER}$ GFP:DAPI signal for the same genotypes as above, in bleomycin-treated flies. Loss of clock activity in ISCs and EBs does not alter rhythms in ECs (left); however, loss of clock in ECs reduces reporter rhythm in ISCs and EBs compared with undamaged conditions (center). *CYC RNAi* undamaged versus *Cyc RNAi* bleomycin-treated (two-way ANOVA F = 18.05, $p < 0.0001$). Under LD conditions, ISC/EB rhythm is normal (one-way ANOVA F = 3.72, p = 0.0032).

(G) Analysis of *Clock*$^{PER}$ GFP:DAPI signal in aged flies. During aging, both controls (*Luc RNAi*) have persistent rhythms when clock activity is lost in ISC/EBs. Loss of clock activity in ECs causes the ISC/EB clock to have very low amplitude (one-way ANOVA F = 10.45, $p < 0.0001$), which is significantly altered compared with undamaged conditions: *CYC RNAi* undamaged versus *Cyc RNAi* aged (two-way ANOVA F = 16.56, $p < 0.0001$). Under LD conditions, the ISC/EB rhythm is again rescued (one-way ANOVA F = 6.447, $p < 0.0001$). Data presented as mean ($n \geq 8$ guts), error bars show ±SEM.

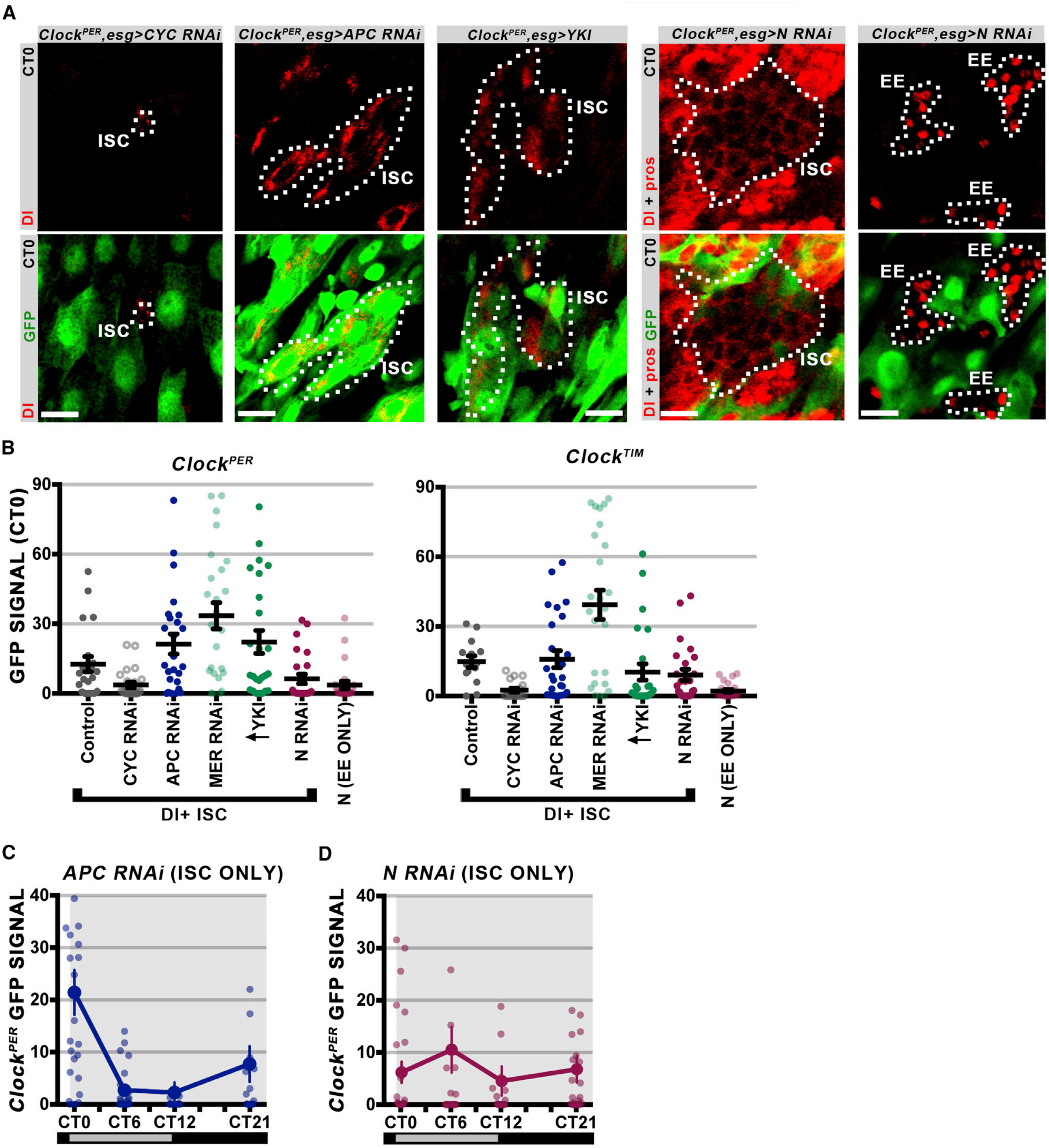


**Figure 5. Stem Cell Signaling Pathways Affect ISC Circadian Clock Activity**

(A) Representative confocal z stacks of *Clock*$^{PER}$ reporter for ISC/EB-specific pathway disruption at CT0. Dl+ and Pros+ are used to mark the ISC versus EE tumors, respectively. ISCs expressing *esg* in these tumors are outlined, showing the abnormal Dl+ cells caused by signaling pathway activation. CYC RNAi and N RNAi reduce GFP+, while APC RNAi and Yki overexpression increase it. Scale bar represents 10 μm.

(B) Analysis of *Clock*$^{PER}$ and *Clock*$^{TIM}$ GFP signals in single Dl+ cells from confocal sections at CT0. Activation of the Wnt or the Hippo pathway raises clock activity, and loss of the N pathway lowers clock activity. Data presented for $n \geq 15$ cells in each group, error bars show ±SEM. One-way ANOVA for *Clock*$^{PER}$ ($F = 9.624$, $p < 0.0001$); *Clock*$^{TIM}$ ($F = 12.99$, $p < 0.0001$).

*(legend continued on next page)*

ISCs are highly sensitive to signals present in their niche that modulate tissue regeneration (Biteau et al., 2011). We challenged the *Clock*$^{PER}$ intestine to chemical damage (bleomycin) that results in the upregulation of signaling pathways involved in the rapid division of stem cells (Amcheslavsky et al., 2009). We first tested whether clock activity was altered in the different intestinal cell types under these conditions or whether they maintained their normal clock function. At CT0, ISCs, EBs, and ECs continued to show CLK/CYC transcriptional activity, while EEs remain clock dead (Figure 4C). Similar to undamaged conditions, cell-specific knockdown of *CYC* in ECs produces a similar rhythm as in controls (Figure 4F), again suggesting that differentiated cells are resistant to the clock status of their undifferentiated neighbors. However, when CYC is disrupted in differentiated ECs, undifferentiated precursors have a phase-shifted and lower clock rhythm than the one present under undamaged conditions (Figure 4F; compare with 3D). However, photoperiod is able to rescue clock function in chemically stressed ISC/EBs (Figure 4F), suggesting direct light is the dominant factor in ISC/EB clock function.

Several recent studies have examined clock function during aging, where alterations in circadian clock target genes are thought to contribute to changes in gene expression and tissue physiology (Kuintzle et al., 2017; Solanas et al., 2017). We repeated our cell-specific knockdown of *CYC* in *Drosophila* aged >35 days, a time when stem cell dysfunction and stress signaling pathways become elevated (Biteau et al., 2008, 2011). The *Clock*$^{PER}$ reporter in aged cells showed very similar profiles to those observed under bleomycin-induced stress: ISCs, EBs, and ECs have CLK/CYC activity, whereas EEs are GFP negative (Figure 4D). Similar to chemical stress, the loss of CYC in ISCs/EBs had little effect on the circadian rhythms exhibited by ECs (Figure 4G); however, the loss of CYC in ECs caused these precursors to exhibit very low rhythmicity (Figure 4G), especially compared with young ISCs (Figure 3D) and undamaged conditions (Figure 4E). Similar to acute stress, photoperiod rescues clock function in ISC/EBs even when the surrounding ECs are clock negative (Figure 4G). These results again show that aged ISCs circadian clocks are responsive to clock activity in the surrounding differentiated ECs when photoperiod is absent. Taken together, these data support the existence of backup unidirectional circadian clock synchronization from differentiated ECs to undifferentiated precursors (ISCs and EBs) that is particularly sensitive to acute environmental or age-related stresses.

## Stem Cell Signaling Regulates Circadian Clock Activity

*Drosophila* stem cells are highly responsive to homeostatic cellular signaling pathways that mediate crosstalk between them and the surrounding tissue (Biteau et al., 2011). N signaling occurring between the ISCs and EBs regulate commitment to the EC lineage (Ohlstein and Spradling, 2007), whereas stress activates both the Hippo and Wnt signaling pathways in stem cells to initiate compensatory proliferation during regeneration (Cordero et al., 2012; Karpowicz et al., 2010; Shaw et al., 2010). To test whether these signaling pathways regulate stem cell circadian clock activity, we activated them using the *Gal4-UAS* system in a temperature-restricted fashion using the *Gal80*$^{TS}$ repressor. The *esg-Gal4* driver was used to drive expression in undifferentiated precursors (ISCs + EBs), and flies bearing constructs were raised in restrictive 18°C temperatures (to silence the *Gal4-UAS* system) then shifted to permissive 29°C to activate genes of interest in the adult intestine.

We first targeted the Wnt pathway repressor *APC*, whose loss leads to uncontrolled hyperplasia by promoting the division of stem cells (Lee et al., 2009). Loss of *APC* increased *Clock*$^{PER}$, and to a lesser extent *Clock*$^{TIM}$ reporter activity (Figures 5A and 5B). Similarly, both positive (*Yki* overexpression) and negative (*Mer RNAi*) components of the Hippo pathway increased *Clock*$^{PER}$, and to a lesser extent *Clock*$^{TIM}$, reporter activity (Figures 5A and 5B). This suggests that CLK/CYC activity is increased in ISCs when they are actively proliferating due to active Wnt or Hippo signaling. We further tested the circadian nature of these phenotypes by testing Dl+ ISCs with activated Wnt pathway over 24 hr; ISCs that lose APC function display normal circadian rhythm periodicity (Figure 5C; compare with Figure 3D).

Loss of the N pathway in stem cell precursors leads to the formation of undifferentiated tumors, consistent with previous reports (Ohlstein and Spradling, 2007). Two types of tumors were observed: those composed of EEs (N signaling is low) or stem cells (N signaling is absent) (Figure 5A). ISC tumors show a heterogeneous mixture of reporter GFP+ and GFP− cells, while EE tumors showed lower *Clock*$^{PER}$ and *Clock*$^{TIM}$ reporter expression than controls, at similar levels to cells expressing CYC RNAi (Figures 5A and 5B). This suggests that the low-GFP+ ISCs are those committing to the EE lineage observed in normal (Figure 2D), damaged (Figure 4C), and aging (Figure 4D) conditions, while the high-GFP+ ISCs are those committing to the EC lineage. Disruption of N signaling in ISCs/EBs using the *esg* driver

(C) Twenty-four-hour analysis of *Clock*$^{PER}$ from *esg>APC RNAi*. Elevated Wnt pathway does not alter the phase of normal circadian clock activity in ISCs (compare with Figure 3D). Data presented for n = 25 cells in each time point, error bars show ±SEM (one-way ANOVA F = 9.153, p < 0.0001).

(D) The same analysis on *esg>N RNAi*. In this case, clock activity is not only lowered but becomes arrhythmic. Data presented for n = 25 cells in each time point, error bars show ±SEM (one-way ANOVA F = 0.6754, p = 0.5692).

renders ISCs completely arrhythmic over 24 hr (Figure 5D; compare with Figure 3D). These data suggest that ISC circadian rhythms require some of the cellular signaling pathways that compose the stem cell niche (N signaling).

## DISCUSSION

### Stem Cell Circadian Rhythms Are Regulated by Intestinal Signaling

The circadian clock has emerged as a central regulator of physiological processes and the coordination of those processes throughout the body (Panda, 2016; Xu et al., 2011). To explore questions surrounding the cell-specific nature of circadian rhythms, we tested circadian clock function in the intestinal epithelium during different physiological states. Previous analyses of circadian transcription in the intestine, including those arising from our own studies, have reported gene expression from the average of a heterogeneous mixture of intestinal epithelial cell types (Hoogerwerf et al., 2007, 2008; Karpowicz et al., 2013; Sladek et al., 2007; Stokes et al., 2017). However, global analysis of circadian clock transcriptional function is limited as it reports the average of transcripts abundance throughout this tissue. Here, we show that clock activity occurs in only a subset of cells in the intestinal epithelium, which include ISCs, and that stem cell circadian clock activity can be modulated depending on their response to homeostatic signaling.

ISCs are surrounded by differentiated ECs, which compose the bulk of cells in the epithelium. When the circadian clock is specifically disrupted in ECs, the circadian core pacemaker in ISCs (driven by CLK/CYC transactivation) is reduced (Figures 4E–4G). Our results thus highlight that circadian clock communication exists between ECs and ISCs in the intestinal epithelium, with differentiated cells positively regulating the clocks of neighboring stem cells, particularly during conditions of stress. It is also likely that circadian signaling occurs between ISCs and EBs in this tissue, because disruption of the N pathway in ISC/EBs using the *esg* driver results in loss of rhythmicity in ISCs, which do not require N signaling. This ability to synchronize circadian timing between different intestinal cells bears some similarity to neuronal clocks of the brain, whereby clock-harboring cells communicate with one another to achieve a unified circadian output (Liu et al., 2007; Sabado et al., 2017). Although intestinal cells are clearly not so interdependent as neurons, a large repertoire of signaling processes are known to occur between intestinal cells (Biteau et al., 2011). It is important to note that over long-term, free-running conditions, these signaling pathways cannot maintain synchrony among intestinal cells (Figures 3D and 3E) and that photoperiod is a dominant synchronizer of circadian clocks (Figures 3B and 4E–4G). The physiological relevance of ECs to ISC circadian communication is not clear; they may play a minor role in *Drosophila* where cells are directly responsive to light and may simply fine-tune circadian timing between cells of this tissue. It will be important to extend these findings to systems where cells do not detect light (i.e., mammals) but rely exclusively on cellular communication to set clock function.

Stem cells have been noted to have heterogeneous clock activity (Janich et al., 2011), suggesting that circadian rhythm generation is dynamic in these undifferentiated cells. Indeed, our data shed light on how circadian communication is controlled by signaling: the Wnt and Hippo pathways, which are activated during regeneration, can boost CLK/CYC activity in ISCs (Figures 5A–5D). These and perhaps other pathways would link the cells of the intestine to achieve a unified circadian output during critical times of need. Indeed, we have reported that circadian rhythms in proliferation occur only during regeneration but not during normal tissue renewal in both *Drosophila* and mice (Karpowicz et al., 2013; Stokes et al., 2017). We thus propose that, during regeneration and stress, signaling pathways from the stem cell niche cause circadian rhythms to increase in amplitude in stem cells in order to synchronize the circadian output of these cells with the timing of organism physiology. Our data support that clock activity can be modulated dynamically within the same stem cell depending on environmental context. This suggests that, when tissues proliferate with 24-hr rhythms, this is due to both circadian rhythms occurring in the proliferating cells as well as those occurring in the surrounding cells that send signals to activate proliferation (Karpowicz et al., 2013).

### Enteroendocrine Cells Do Not Exhibit Circadian Clock Activity

EEs do not exhibit reporter expression in either the *PER* or *TIM* reporter constructs examined, and do not exhibit nuclear PER localization by antibody, leading us to conclude that EEs do not have circadian clock function (Figures 2C–2E and S3C–S3G). A recent study has revealed a population of EE precursor cells that respond directly to mechanical stimulation through the receptor, *Piezo* (He et al., 2018). The presence of GFP variability in ISCs and EBs may be due to GFP-low EE precursors that lose clock activity as they differentiate. This notion is consistent with our results indicating that in EE tumors arising from perturbation of the N pathway, EE precursor cells exhibit lower clock reporter expression. This may also explain why Dutta et al. (2015) previously found some clock genes to be expressed in EEs; these genes persist in the EE precursors before they fully differentiate. We thus propose that the

circadian clock persists in intestinal cells committed to an EC fate and is extinguished in cells committing to the EE lineage. As the circadian clock has been proposed to regulate both metabolic (Panda, 2016) and immune functions (Man et al., 2016), it is somewhat surprising that EEs, which in part regulate these processes, would be clock deficient. Although we found neither clock reporter nor protein expression present, it is possible that EEs express low levels of *PER* and *TIM*, unlike the other cells of the intestine, and require additional regulatory sequences in addition to the CYC/CLK binding sites found in the *PER* and *TIM* promoters used in our study. Future work will further determine how and why EEs do not exhibit clock activity in *Drosophila*, whether EEs can turn on circadian clock activity under certain conditions, and whether these findings extend to other animals.

### Feeding Can Entrain *Drosophila* Intestinal Clocks

Like the circadian clock of the *Drosophila* fat body and the mouse liver (Damiola et al., 2000; Xu et al., 2011), clocks in intestinal cells can be regulated by the timing of food uptake. Specifically, restricting the time of feeding can alter CLK/CYC activity in both *CRY* mutants that are insensitive to photoperiod and in wild-type intestines in the absence of photoperiod. Hence, food intake may serve as a secondary entrainment factor in a tissue subject to circadian behavioral rhythms, which include the time of feeding. Because *Drosophila* cell clocks can directly respond to light, and we find that light-driven entrainment appears to predominate, we propose that food entrainment is a second synchronizing cue regulated by signaling processes that occur in digestive tract tissues. Photoperiod remains the dominant entrainment cue, consistent with findings previously documented in *Drosophila* behavior and metabolism studies (Oishi et al., 2004; Xu et al., 2011).

### Do Circadian Rhythms Occur during Pathological Conditions?

These data also provide insight into how normal circadian activity can be disrupted in pathological contexts, such as the process of tumorigenesis where the role of the circadian clock has been controversial (Sancar et al., 2015). It is likely that in different cancers, which may involve the activation or inactivation of different cellular signaling processes in cancer stem cells, circadian clock function may be either present or absent.

It was recently shown that mouse ISCs do not exhibit robust circadian activity *in vitro* (Matsu-Ura et al., 2016), similar to embryonic stem cells (Yagita et al., 2010), which have been shown to develop circadian rhythmicity as they differentiate (Umemura et al., 2017), yet in hair follicle stem cells and muscle stem cells, circadian clock activity has been observed (Janich et al., 2011; Solanas et al., 2017). This raises the question of when clock activity, which is absent in a pluripotent state, emerges as cells undergo differentiation. Our results support that cellular differentiation increases clock activity in the case of ECs, whose precursors are clock active during differentiation. However, the population of ISCs that spawn these is also clock active, and differentiation can conversely lead to termination of clock activity in the case of EE precursors. Our results shed light on why the stem cell population is heterogeneous for clock activity and why the overall readouts of such a population exhibit lower circadian rhythms than differentiated cells (Matsu-Ura et al., 2016). Circadian clock activity is defined in specific cell lineages rather than cell differentiation status and can be modulated under certain conditions. The surrounding differentiated cells signal to ISCs, and the presence or absence of these signals regulates clock function in stem cells. Our *in vivo* data also raise an important caveat to *in vitro* studies of stem cell biology: cellular signals, often present *in vitro* at non-physiological levels, may artificially perturb stem cell clock function. Stem cell clock function examined *in vivo*, in particular experiments involving live imaging of clock activity, would resolve these issues. The reporters generated in this study may be suitable for this purpose.

## EXPERIMENTAL PROCEDURES

Flies were housed at 25°C under an LD cycle on standard media, unless otherwise noted. At each time point ~10 intestines from female flies <14 days were dissected in PBS (Fisher) and fixed in 4% paraformaldehyde (Electron Microscopy Sciences) in PBS and then counterstained with DAPI (Thermo Fisher Scientific, 1:5,000) in PBS-T (PBS + 0.2% Triton X-100, Fisher). Intestines were then blocked in 1% BSA (Bio Basic) + 0.2%Triton X-100 (Fisher) and incubated in the same at room temperature for 2 hr with primary antibodies: mouse anti-Delta (Developmental Studies Hybridoma Bank [DSHB], 1:50), mouse anti-prospero (DSHB, 1:50), mouse anti-histone (Millipore, 1:2000), or rabbit anti-PER (generously provided by Patrick Emery, 1:1,500), then incubated at room temperature for 1 hr in secondary goat anti-mouse/rabbit antibodies (Life Technologies, 1:2000), and counterstained with DAPI (Thermo Fisher Scientific, 1:5,000). Samples were imaged using a slide scanner (Zeiss Axio Scan.Z1) that assembled single images consisting of merged and tiled z stacks of the entire tissue sample in a single plane of focus, or by confocal microscopy (Olympus IX81 FV1000) with a 60× water-immersion lens. Images were analyzed using Zen Blue Edition software (Zeiss) and processed using Photoshop CS5 (Adobe).

### Generation of Clock Reporters

The enhancer of Per and Tim were synthesized as gBlocks (Integrated DNA Technologies) in 2× tandem multiplexes. Then two copies of the gBlocks were amplified by PCR and inserted into a reporter vector using In-Fusion HD Cloning (Clontech) to

generate 4× Per and 4× Tim enhancers (Figure S4). The reporter vector contains fly heat shock mini promoter and an nlsGFP fused with PEST domain at the C terminus (destabilized GFP) as previously used for the construction of Notch reporter(Hunter et al., 2016).

### RT-qPCR

Flies were synchronized to LD cycles as described above. At each time point, ~10 intestines were dissected in PBS (Fisher) and stored in RNAlater reagent (Qiagen). Intestines were homogenized in RLT Buffer (Qiagen) using a Bullet blender as directed by the manufacturers protocol (Next Advance). RNA was extracted using the RNeasy Mini Kit (Qiagen). cDNA was prepared using ISCRIPT RT Supermix (Bio-Rad). qPCR was carried out using iTaq Universal SYBR Green Supermix (Bio-Rad) on the ViiA7 PCR plate reader.

### Quantification and Statistical Analysis

Fluorescence intensities were obtained using Zen Blue Software (Zeiss) and examined for changes in the ratio of average GFP:DAPI signal over time, except for Figure 3D, in which the total GFP:DAPI for only n = 5 individual cells of each cell type in a single 2-μm section was used. Whole-gut measurements include regions R1 to R5, anterior measurements include region R2 only, and posterior include R5 only. For confocal images, GFP measurements from single optical sections were quantified using ImageJ. Cell types were identified by antibody staining and morphology. Results are reported as mean ± SEM, and statistical significance was established using one-way ANOVA unless otherwise stated. All results were analyzed using Prism (GraphPad); n values refer to number of intestines, except for confocal image analysis, where n = number of cells.

For details of fly stocks and primers see Supplemental Experimental Procedures.

## SUPPLEMENTAL INFORMATION

Supplemental Information includes Supplemental Experimental Procedures and three figures and can be found with this article online at https://doi.org/10.1016/j.stemcr.2018.10.010.

## AUTHOR CONTRIBUTIONS

K.P. and N.B. conducted experiments, analyzed data, and wrote the paper. M.H., H.C., and L.H. conducted experiments. L.H. and N.P. designed the clock reporter vectors and helped write the figures. P.K. designed the clock reporter promoters, designed the experiments, and wrote the paper.

## ACKNOWLEDGMENTS

Fly stocks were provided by the Bloomington *Drosophila* Stock Center. We thank Patrick Emery and Yong Zhang for reading and evaluating a draft of the manuscript. We also thank members of the Karpowicz and Perrimon labs for their help, and in particular Sharon Lavigne and Richard Binari for their technical assistance. This work was supported by Natural Sciences and Engineering Research Council (P.K.), and the Damon Runyan Cancer Research Foundation (L.H.). N.P. is an investigator of the Howard Hughes Medical Institute.

Received: June 6, 2018
Revised: October 9, 2018
Accepted: October 10, 2018
Published: November 13, 2018

## REFERENCES

Agrawal, P., Houl, J.H., Gunawardhana, K.L., Liu, T., Zhou, J., Zoran, M.J., and Hardin, P.E. (2017). *Drosophila* CRY entrains clocks in body tissues to light and maintains passive membrane properties in a non-clock body tissue independent of light. Curr. Biol. *27*, 2431–2441.

Amcheslavsky, A., Jiang, J., and Ip, Y.T. (2009). Tissue damage-induced intestinal stem cell division in *Drosophila*. Cell Stem Cell *4*, 49–61.

Balsalobre, A., Marcacci, L., and Schibler, U. (2000). Multiple signaling pathways elicit circadian gene expression in cultured Rat-1 fibroblasts. Curr. Biol. *10*, 1291–1294.

Beebe, K., Park, D., Taghert, P.H., and Micchelli, C.A. (2015). The *Drosophila* prosecretory transcription factor dimmed is dynamically regulated in adult enteroendocrine cells and protects against gram-negative infection. G3 (Bethesda) *5*, 1517–1524.

Biteau, B., Hochmuth, C.E., and Jasper, H. (2008). JNK activity in somatic stem cells causes loss of tissue homeostasis in the aging *Drosophila* gut. Cell Stem Cell *3*, 442–455.

Biteau, B., Hochmuth, C.E., and Jasper, H. (2011). Maintaining tissue homeostasis: dynamic control of somatic stem cell activity. Cell Stem Cell *9*, 402–411.

Brown, S.A. (2014). Circadian clock-mediated control of stem cell division and differentiation: beyond night and day. Development *141*, 3105–3111.

Buchon, N., Osman, D., David, F.P., Fang, H.Y., Boquete, J.P., Deplancke, B., and Lemaitre, B. (2013). Morphological and molecular characterization of adult midgut compartmentalization in *Drosophila*. Cell Rep. *3*, 1725–1738.

Cordero, J.B., Stefanatos, R.K., Scopelliti, A., Vidal, M., and Sansom, O.J. (2012). Inducible progenitor-derived wingless regulates adult midgut regeneration in *Drosophila*. EMBO J. *31*, 3901–3917.

Damiola, F., Le Minh, N., Preitner, N., Kornmann, B., Fleury-Olela, F., and Schibler, U. (2000). Restricted feeding uncouples circadian oscillators in peripheral tissues from the central pacemaker in the suprachiasmatic nucleus. Genes Dev. *14*, 2950–2961.

Dutta, D., Dobson, A.J., Houtz, P.L., Gläßer, C., Revah, J., Korzelius, J., Patel, P.H., Edgar, B.A., and Buchon, N. (2015). Regional cell-specific transcriptome mapping reveals regulatory complexity in the adult *drosophila* midgut. Cell Rep. *12*, 346–358.

Emery, P., So, W.V., Kaneko, M., Hall, J.C., and Rosbash, M. (1998). CRY, a *Drosophila* clock and light-regulated cryptochrome, is a major contributor to circadian rhythm resetting and photosensitivity. Cell *95*, 669–679.

Hao, H., Allen, D.L., and Hardin, P.E. (1997). A circadian enhancer mediates PER-dependent mRNA cycling in *Drosophila melanogaster*. Mol. Cell. Biol. *17*, 3687–3693.

Hardin, P.E. (2011). Molecular genetic analysis of circadian timekeeping in *Drosophila*. Adv. Genet. *74*, 141–173.

He, L., Si, G., Huang, J., Samuel, A.D.T., and Perrimon, N. (2018). Mechanical regulation of stem-cell differentiation by the stretch-activated Piezo channel. Nature *555*, 103–106.

Hoogerwerf, W.A., Hellmich, H.L., Cornélissen, G., Halberg, F., Shahinian, V.B., Bostwick, J., Savidge, T.C., and Cassone, V.M. (2007). Clock gene expression in the murine gastrointestinal tract: endogenous rhythmicity and effects of a feeding regimen. Gastroenterology *133*, 1250–1260.

Hoogerwerf, W.A., Sinha, M., Conesa, A., Luxon, B.A., Shahinian, V.B., Cornélissen, G., Halberg, F., Bostwick, J., Timm, J., and Cassone, V.M. (2008). Transcriptional profiling of mRNA expression in the mouse distal colon. Gastroenterology *135*, 2019–2029.

Hunter, G.L., Hadjivasiliou, Z., Bonin, H., He, L., Perrimon, N., Charras, G., and Baum, B. (2016). Coordinated control of Notch/Delta signalling and cell cycle progression drives lateral inhibition-mediated tissue patterning. Development *143*, 2305–2310.

Ivanchenko, M., Stanewsky, R., and Giebultowicz, J.M. (2001). Circadian photoreception in *Drosophila*: functions of cryptochrome in peripheral and central clocks. J. Biol. Rhythms *16*, 205–215.

Janich, P., Pascual, G., Merlos-Suárez, A., Batlle, E., Ripperger, J., Albrecht, U., Cheng, H.Y., Obrietan, K., Di Croce, L., and Benitah, S.A. (2011). The circadian molecular clock creates epidermal stem cell heterogeneity. Nature *480*, 209–214.

Karpowicz, P., Perez, J., and Perrimon, N. (2010). The Hippo tumor suppressor pathway regulates intestinal stem cell regeneration. Development *137*, 4135–4145.

Karpowicz, P., Zhang, Y., Hogenesch, J.B., Emery, P., and Perrimon, N. (2013). The circadian clock gates the intestinal stem cell regenerative state. Cell Rep. *3*, 996–1004.

Kuintzle, R.C., Chow, E.S., Westby, T.N., Gvakharia, B.O., Giebultowicz, J.M., and Hendrix, D.A. (2017). Circadian deep sequencing reveals stress-response genes that adopt robust rhythmic expression during aging. Nat. Commun. *8*, 14529.

Lee, W.C., Beebe, K., Sudmeier, L., and Micchelli, C.A. (2009). Adenomatous polyposis coli regulates *Drosophila* intestinal stem cell proliferation. Development *136*, 2255–2264.

Liu, A.C., Welsh, D.K., Ko, C.H., Tran, H.G., Zhang, E.E., Priest, A.A., Buhr, E.D., Singer, O., Meeker, K., Verma, I.M., et al. (2007). Intercellular coupling confers robustness against mutations in the SCN circadian clock network. Cell *129*, 605–616.

Man, K., Loudon, A., and Chawla, A. (2016). Immunity around the clock. Science *354*, 999–1003.

Matsu-Ura, T., Dovzhenok, A., Aihara, E., Rood, J., Le, H., Ren, Y., Rosselot, A.E., Zhang, T., Lee, C., Obrietan, K., et al. (2016). Intercellular coupling of the cell cycle and circadian clock in adult stem cell culture. Mol. Cell *64*, 900–912.

McDonald, M.J., Rosbash, M., and Emery, P. (2001). Wild-type circadian rhythmicity is dependent on closely spaced E boxes in the *Drosophila* timeless promoter. Mol. Cell. Biol. *21*, 1207–1217.

Nagoshi, E., Saini, C., Bauer, C., Laroche, T., Naef, F., and Schibler, U. (2004). Circadian gene expression in individual fibroblasts: cell-autonomous and self-sustained oscillators pass time to daughter cells. Cell *119*, 693–705.

Ohlstein, B., and Spradling, A. (2007). Multipotent *Drosophila* intestinal stem cells specify daughter cell fates by differential notch signaling. Science *315*, 988–992.

Oishi, K., Shiota, M., Sakamoto, K., Kasamatsu, M., and Ishida, N. (2004). Feeding is not a more potent Zeitgeber than the light-dark cycle in *Drosophila*. Neuroreport *15*, 739–743.

Panda, S. (2016). Circadian physiology of metabolism. Science *354*, 1008–1015.

Park, J.H., Chen, J., Jang, S., Ahn, T.J., Kang, K., Choi, M.S., and Kwon, J.Y. (2016). A subset of enteroendocrine cells is activated by amino acids in the *Drosophila* midgut. FEBS Lett. *590*, 493–500.

Reppert, S.M., and Weaver, D.R. (2002). Coordination of circadian timing in mammals. Nature *418*, 935–941.

Sabado, V., Vienne, L., and Nagoshi, E. (2017). Evaluating the autonomy of the *Drosophila* circadian clock in dissociated neuronal culture. Front. Cell. Neurosci. *11*, 317.

Sancar, A., Lindsey-Boltz, L.A., Gaddameedhi, S., Selby, C.P., Ye, R., Chiou, Y.Y., Kemp, M.G., Hu, J., Lee, J.H., and Ozturk, N. (2015). Circadian clock, cancer, and chemotherapy. Biochemistry *54*, 110–123.

Shaw, R.L., Kohlmaier, A., Polesello, C., Veelken, C., Edgar, B.A., and Tapon, N. (2010). The Hippo pathway regulates intestinal stem cell proliferation during *Drosophila* adult midgut regeneration. Development *137*, 4147–4158.

Sladek, M., Rybová, M., Jindráková, Z., Zemanová, Z., Polidarová, L., Mrnka, L., O'Neill, J., Pácha, J., and Sumová, A. (2007). Insight into the circadian clock within rat colonic epithelial cells. Gastroenterology *133*, 1240–1249.

Solanas, G., Peixoto, F.O., Perdiguero, E., Jardí, M., Ruiz-Bonilla, V., Datta, D., Symeonidi, A., Castellanos, A., Welz, P.S., Caballero, J.M., et al. (2017). Aged stem cells reprogram their daily rhythmic functions to adapt to stress. Cell *170*, 678–692.e20.

Song, W., Veenstra, J.A., and Perrimon, N. (2014). Control of lipid metabolism by tachykinin in *Drosophila*. Cell Rep. *9*, 40–47.

Stanewsky, R., Jamison, C.F., Plautz, J.D., Kay, S.A., and Hall, J.C. (1997). Multiple circadian-regulated elements contribute to cycling period gene expression in *Drosophila*. EMBO J. *16*, 5006–5018.

Stokes, K., Cooke, A., Chang, H., Weaver, D.R., Breault, D.T., and Karpowicz, P. (2017). The circadian clock gene BMAL1 coordinates intestinal regeneration. Cell. Mol. Gastroenterol. Hepatol. *4*, 95–114.

Tognini, P., Murakami, M., Liu, Y., Eckel-Mahan, K.L., Newman, J.C., Verdin, E., Baldi, P., and Sassone-Corsi, P. (2017). Distinct circadian signatures in liver and gut clocks revealed by ketogenic diet. Cell Metab. *26*, 523–538.e5.

Umemura, Y., Koike, N., Ohashi, M., Tsuchiya, Y., Meng, Q.J., Minami, Y., Hara, M., Hisatomi, M., and Yagita, K. (2017). Involvement of posttranscriptional regulation of Clock in the emergence of circadian clock oscillation during mouse development. Proc. Natl. Acad. Sci. U S A *114*, E7479–E7488.

Vatine, G., Vallone, D., Gothilf, Y., and Foulkes, N.S. (2011). It's time to swim! Zebrafish and the circadian clock. FEBS Lett. *585*, 1485–1494.

Xu, K., Zheng, X., and Sehgal, A. (2008). Regulation of feeding and metabolism by neuronal and peripheral clocks in *Drosophila*. Cell Metab. *8*, 289–300.

Xu, K., DiAngelo, J.R., Hughes, M.E., Hogenesch, J.B., and Sehgal, A. (2011). The circadian clock interacts with metabolic physiology to influence reproductive fitness. Cell Metab. *13*, 639–654.

Yagita, K., Horie, K., Koinuma, S., Nakamura, W., Yamanaka, I., Urasaki, A., Shigeyoshi, Y., Kawakami, K., Shimada, S., Takeda, J., and Uchiyama, Y. (2010). Development of the circadian oscillator during differentiation of mouse embryonic stem cells in vitro. Proc. Natl. Acad. Sci. U S A *107*, 3846–3851.

Yeom, M., Pendergast, J.S., Ohmiya, Y., and Yamazaki, S. (2010). Circadian-independent cell mitosis in immortalized fibroblasts. Proc. Natl. Acad. Sci. U S A *107*, 9665–9670.

Zhang, R., Lahens, N.F., Ballance, H.I., Hughes, M.E., and Hogenesch, J.B. (2014). A circadian gene expression atlas in mammals: implications for biology and medicine. Proc. Natl. Acad. Sci. U S A *111*, 16219–16224.

Stem Cell Reports, Volume *11*

## Supplemental Information

# Intestinal Stem Cells Exhibit Conditional Circadian Clock Function

**Kathyani Parasram, Nathaniel Bernardon, Maha Hammoud, Hanna Chang, Li He, Norbert Perrimon, and Phillip Karpowicz**

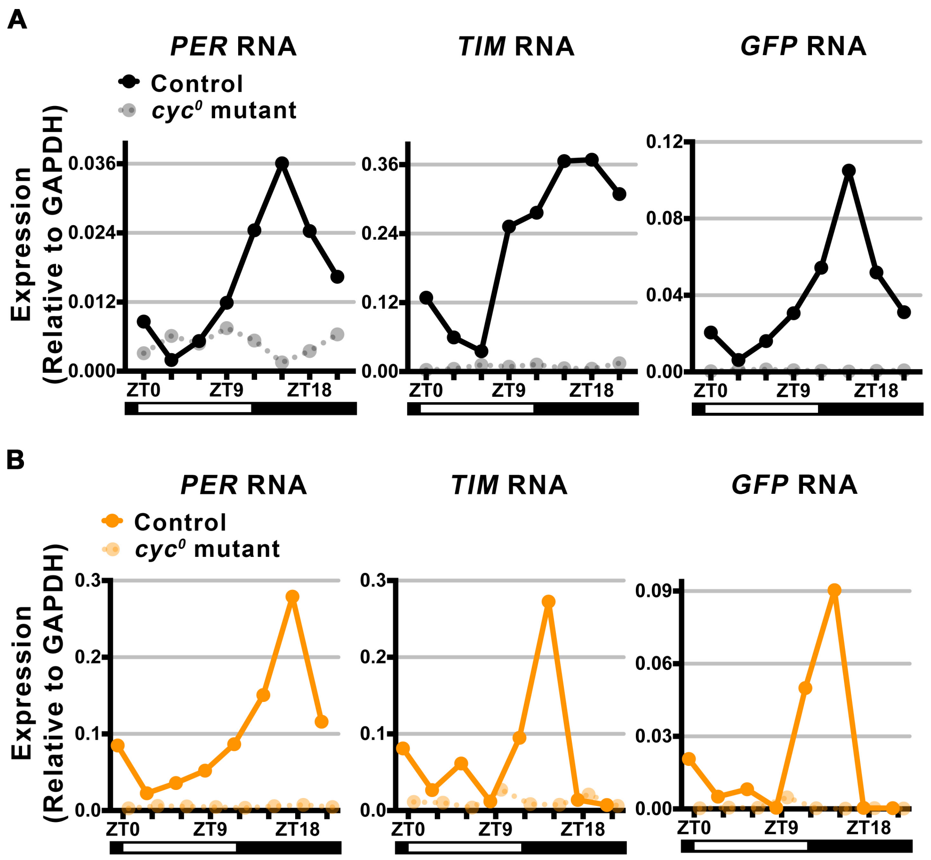


**Figure S1: Analysis of *Clock*$^{PER}$ and *Clock*$^{TIM}$ reporter transcription. Related to Figure 1.** **(A)** A second representative experiment showing RT-qPCR expression for *Clock*$^{PER}$ (*GFP* RNA), *PER* and *TIM* RNA. Each data point represents a signal obtained from n=10 intestines. Data confirms the expression rhythms shown in Fig 1E. **(B)** RT-qPCR expression for *Clock*$^{TIM}$ (*GFP* RNA), *PER* and *TIM* RNA. Each data point represents a signal obtained from n=10 intestines. As in Fig 1E and above, all three genes in show similar expression phases indicating similar timing of CLK/CYC activity on the *TIM* promoter as on the *PER* promoter.

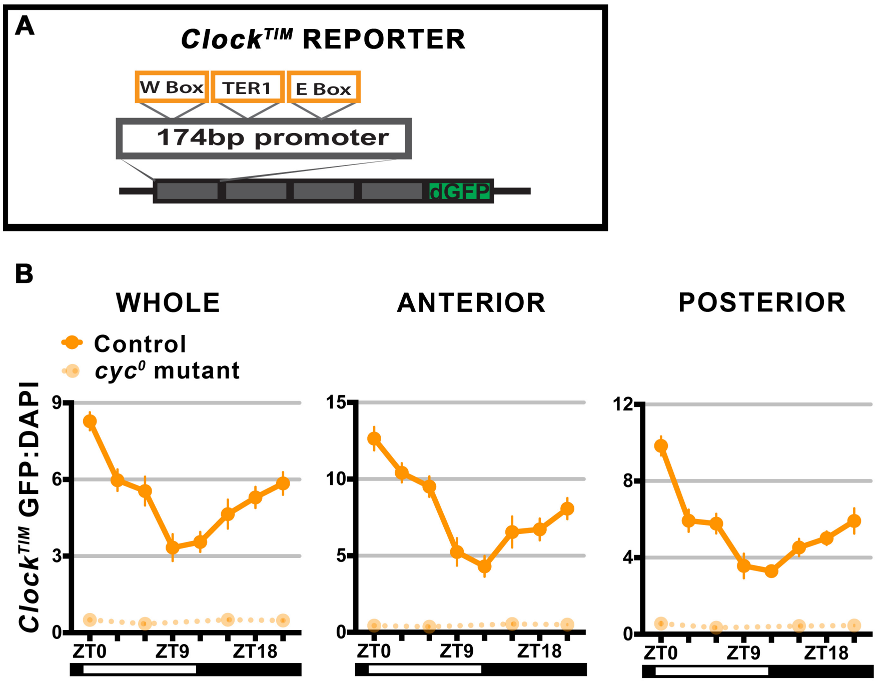


**Figure S2: The *Clock*$^{TIM}$ reporter reports circadian clock activity. Related to Figure 1 and Figure 2A. (A)** Schematic of the *Clock*$^{TIM}$ reporter shows four copies of the 174bp minimal promoter upstream of destabilized GFP (dGFP) to report temporal changes in circadian clock activity through *TIM* expression. **(B)** Analysis of *Clock*$^{TIM}$ GFP:DAPI signal under LD photoperiod for the whole intestine, anterior (R2), and posterior (R5) regions. All regions show similar phase of clock reporter activity, and the *cyc*$^0$ mutant has no circadian transactivation and is GFP low. Data presented as mean of n≥10 intestines, error bars show ± SEM. Control *vs*. *cyc*$^0$: whole gut (2-way ANOVA F=6.52, p=0.0006), anterior (2-way ANOVA F=7.172, p=0.0003), posterior (2-way ANOVA F=11.2, p<0.0001).

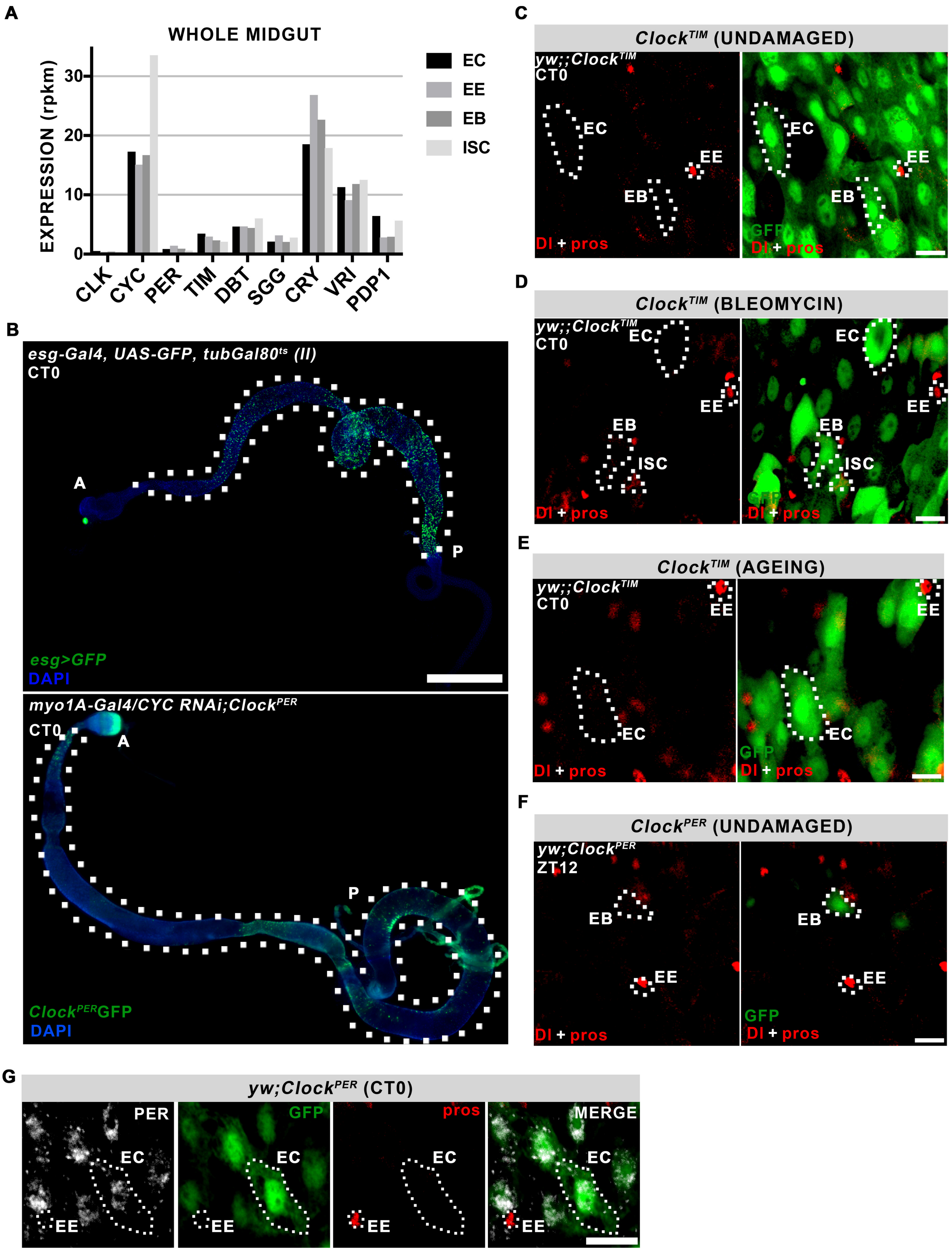


**Figure S3: Single cell analysis of EE and ISC/EB cells in intestine. Related to Figure 2.** (see next page)

**Figure S3: Single cell analysis of EE and ISC/EB cells in intestine. Related to Figure 2. (A)** Graph depicting the RNA-sequencing expression of clock genes modified from *flygutseq.buchonlab.com*[26]. Data shows similar gene expression levels for all clock genes in all four intestinal epithelial cell types, although we note that the expression of CLK is particularly low in this dataset. The phase of circadian clock activity and the dissection time is not available from this dataset which could greatly affect clock gene levels. **(B)** Representative images of *Drosophila* intestines at CT0 comparing the number of undifferentiated precursors marked by GFP in the *esg-Gal4, UAS-GFP, tubGal80$^{TS}$* genotype which marks all ISC/EBs when flies are shifted to 29°C temperature, *vs*. the number of undifferentiated precursors marked by *Clock$^{PER}$* GFP+ when *CYC* is knocked down in ECs (*Myo1A>CYC*) at its highest time of expression. Note that many small GFP+ cells that are present in the former, are absent in the latter. DAPI counterstains nuclei, A: indicates the anterior region, P: posterior. Scale bar is 500μm. **(C)** Representative confocal Z-stack of *Clock$^{TIM}$* at CT0 shows that EEs do not express GFP+ reporter signal from the *TIM* reporter either. **(D and E)** Confocal section of *Clock$^{TIM}$* at CT0 shows that EE cells do not express GFP from the *TIM* reporter under bleomycin-treated or ageing conditions. Dl+ and pros+ marking the ISCs and EEs, respectively, are outlined, as is a large polyploid EC and differentiating EB, as indicated, scale bar is 10μm. **(F)** Confocal section of *Clock$^{PER}$* at ZT12, the trough of GFP+ expression, shows that EE cells do not express *PER* reporter activity in an anti-phasic fashion either. An EE is outlined, as is an EB showing low levels of GFP+ signal at this time, scale bar is 10μm. **(G)** Confocal section showing nuclear PER antibody staining in ECs, but not EEs in the *Clock$^{PER}$* reporter that co-expresses PER and GFP simultaneously, indicating no circadian clock function. EEs do not show either expression of PER or GFP. Cells of interest are outlined: pros+ marks EEs, ECs are the large polyploid cells. Scale bar is 10μm.

## Supplemental Experimental Procedures

### Fly Strains

Flies were housed at 25°C under a 12-h light:12-h dark cycle (LD) on media containing 1.2% w/v dry yeast, 0.7% w/v soyflour, 5% w/v cornmeal, 0.4% w/v malt, 0.4% v/v agar, 5.3% v/v with propionic acid, and tegosept (Diamed). Experiments with temperature-induced expression (Fig 5) were carried out in the exact same conditions except at 29°C for 7 days. In all experiments, flies were tested following >5 days synchronization to LD schedules. In the free-running experiments, flies were moved to 24h dark conditions (DD) and tested the following day after moving, or 5 or 10 days after free-running DD conditions (as indicated in the Figures). Female flies <14 days old (at time of dissection) were used for experiments, except for ageing experiments which used female flies aged 35-40 days. For bleomycin-treatment, 2 days prior to the experiment flies were put on food as above but containing 25µg/mL bleomycin (Millipore). Restricted feeding was performed by moving flies from hydration-supplemented vials that contained 2.4g of agar in 500mL water to vials containing food (at the times indicated in the text).

*CYC RNAi* constructs, which have been previously validated for efficient cell-specific circadian disruption[S1], were used with the *esg-Gal4* driver to disrupt *CYC* in undifferentiated precursors (ISCs + EBs) or with the *Myo1A-Gal4* driver to disrupt *CYC* in differentiated ECs. Our previous work has also shown these *Gal4* drivers are not expressed in clock neurons, and hence do not affect circadian behavior [S1].

The following strains were used:
*ClockPER on II (attP40)*
*ClockPER on III (attP2)*
*ClockTIM on II (attP40)*
*ClockTIM on III (attP2)*
$cyc^{0}, ry^{506}$
$w^{1118};;cry^{01}$
*esg-Gal4 (II)*
*myo1A-Gal4 (II)*
*yw; PER-HGM*
*w;TIM-HGM*
*w;;TIM-HGM*
*Luc RNAi* (Bloomington stock center #31603)
*cyc RNAi* (Bloomington stock center #42563)
*Mer RNAi* (Bloomington stock center #28007)
*APC RNAi* (Bloomington stock center #34869)
*N RNAi* (Bloomington stock center #33611)
*UAS-Yki*

Experimental fly strains, organized by figure:

| Figure | | Fly Strain |
|---|---|---|
| 1 | C | $yw;Clock^{PER};$ |
| | D | $yw;Clock^{PER};$ |
| | E | $yw;Clock^{PER};$<br>$yw;Clock^{PER}/CyO;cyc^{0},ry^{506}/TM6B$ |
| | F | $yw;Clock^{PER};$<br>$yw;Clock^{PER}/CyO;cyc^{0},ry^{506}/TM6B$ |
| 2 | A | $yw;Clock^{PER};$<br>$yw;Clock^{PER}/CyO;cyc^{0},ry^{506}/TM6B$ |
| | C | $yw;Clock^{PER};$ |
| | D | $yw;Clock^{PER};$ |
| | E | $w^{1118}$ |
| | A | $yw;Clock^{PER};$ |

| | |
|---|---|
| B | *yw;Clock^PER;*<br>*;Clock^PER;cry^01* |
| C | *yw;Clock^PER;* |
| D | *yw;Clock^PER;* |
| E | *yw;Clock^PER;* |
| A | *esg-Gal4,UAS-CD8RFP/Valium20-Cyc;Clock^PER/MKRS* |
| B | *Myo1A-Gal4/Valium20-Cyc;Clock^PER/MKRS* |
| C | *yw;Clock^PER;* |
| D | *yw;Clock^PER;* |
| E | *Myo1A-Gal4/IF;Clock^PER/Valium1-Luc*<br>*Myo1A-Gal4/Valium20-Cyc;Clock^PER/MKRS*<br>*esg-Gal4,UAS-CD8RFP/IF;Clock^PER/Valium1-Luc*<br>*esg-Gal4,UAS-CD8RFP/Valium20-Cyc;Clock^PER/MKRS* |
| F | same as 4E |
| G | same as 4E |
| A | *esg-Gal4/Clock^PER;tubGal80^TS/Valium20-Cyc*<br>*esg-Gal4/Clock^PER;tubGal80^TS/Valium20-APC*<br>*esg-Gal4/Clock^PER;tubGal80^TS/UAS-Yki*<br>*esg-Gal4/Clock^PER;tubGal80^TS/Valium20-N* |
| B | *esg-Gal4/Clock^PER;tubGal80^TS/+*<br>*esg-Gal4/Clock^PER;tubGal80^TS/Valium20-Cyc*<br>*esg-Gal4/Clock^PER;tubGal80^TS/Valium20-APC*<br>*esg-Gal4/Clock^PER;tubGal80^TS/Valium10-Mer*<br>*esg-Gal4/Clock^PER;tubGal80^TS/UAS-Yki*<br>*esg-Gal4/Clock^PER;tubGal80^TS/Valium20-N*<br>*esg-Gal4/Clock^TIM;tubGal80^TS/+*<br>*esg-Gal4/Clock^TIM;tubGal80^TS/Valium20-Cyc*<br>*esg-Gal4/Clock^TIM;tubGal80^TS/Valium20-APC*<br>*esg-Gal4/Clock^TIM;tubGal80^TS/Valium10-Mer*<br>*esg-Gal4/Clock^TIM;tubGal80^TS/UAS-Yki*<br>*esg-Gal4/Clock^TIM;tubGal80^TS/Valium20-N* |
| C | *esg-Gal4/Clock^PER;tubGal80^TS/Valium20-APC* |
| D | *esg-Gal4/Clock^PER;tubGal80^TS/Valium20-N* |
| A | *yw;Clock^PER;*<br>*yw;Clock^PER/CyO;cyc^0,ry^506/TM6B* |
| B | *w;Clock^TIM*<br>*yw;Clock^TIM/CyO;cyc^0,ry^506/TM6B* |
| B | *w;Clock^TIM*<br>*yw;Clock^TIM/CyO;cyc^0,ry^506/TM6B* |
| B | *esg-Gal4,UAS-GFP,tubGal80^TS;*<br>*Myo1A-Gal4/Valium20-Cyc;Clock^PER/MKRS* |
| C | *w;;Clock^TIM* |
| D | *w;;Clock^TIM* |
| E | *w;;Clock^TIM* |
| F | *yw;Clock^PER* |
| G | *yw;Clock^PER* |

**Generation of Clock reporters**

The following primers were used:
gBlock1 forward 5' AACCAACAACTCTAGACTACTGC 3'
gBlock1 reverse 5' ACTGCTGAGTCAGACTATCTGGATCCTCTAGACGTGC 3'
gBlock1 forward 5' AGTCTGACTCAGCAGTAACCAACAACTCTAGACTACTGC 3'
gBlock1 reverse 5'ATCTGGATCCTCTAGACGTGC 3'

Sequence of Per enhancer:
CGAGTTCGCACAACTCATGGTGGGGCAGGGACGGGTCCGAGTGCAAAAAGCGTCCGAGAAACCGTAG
GCAGTGAAAAGCCGCCGCTCACGTGGCGAACTGCGTGACTTGGCCAGCAAATCCGC

Sequence of Tim enhancer:
TTCCGGCGCTGGTTATTCATGTTATCGAACATCGTAGTGGCGGTTGGCAAATAAACGTGCGGCACGTT
GTGATTACACGTGAGCCGATTTCCCCGGCCGTCCGGCATTGAGTGCGAGCGGGACGGAGCAGCGCGGC
GAGAGCTTTCGGCTAGCGTCTTTCGTTCGCACAAACGC

The full-length sequences for the plasmids can be found in the supplementary data file.

**RT-qPCR**

The following primers were used:
*GFP* Forward: TTTCATCACCCTCCTCCAAG
*GFP* Reverse: TTATGCCCGAGTTCTTTTCG
*PER* Forward: TCATCCAGAACGGTTGCTACG
*PER* Reverse: CCTGAAAGACGCGATGGTGT
*TIM* Forward: CCAGCATTCATTCCAAGCAG
*TIM* Reverse: GCGTGGCAAACTGTGTTATG
*GAPDH* Forward: CCAATGTCTCCGTTGTGGA
*GAPDH* Reverse: TCGGTGTAGCCCAGGATT

**Tissue Fluorescence Imaging**

Flies were synchronized to LD cycles as described above. At each time point ~10 intestines were dissected in PBS (Fisher), and fixed in 4% PFA (Electron microscopy sciences) in PBS for 40 minutes at room temperature. Intestines were rinsed 3x using PBS, and then counterstained with DAPI (ThermoFisher Scientific, 1:5000) in PBS-T (PBS + 0.2% Triton X-100, Fisher) for 5 minutes. Intestines were then washed with PBS-T and mounted on slides with ProLong Gold antifade reagent (ThermoFisher Scentific). Samples were imaged using a slide scanner (Zeiss Axio Scan.Z1) that assembled single images consisting of merged and tiled Z-stacks of the entire tissue sample in a single plane of focus. Images were analyzed using Zen Blue Edition software (Zeiss). Images were processed using Photoshop CS5 (Adobe).

**Supplemental Reference**

S1. Karpowicz, P., Zhang, Y., Hogenesch, J.B., Emery, P., and Perrimon, N. (2013). The circadian clock gates the intestinal stem cell regenerative state. Cell reports *3*, 996-1004.

**Additional Items**

**A) 4xPER Sequence**

```
LOCUS       Exported               11609 bp ds-DNA     circular SYN 17-SEP-2018
DEFINITION  synthetic circular DNA
ACCESSION   .
VERSION     .
KEYWORDS    4XPer nls-sfGFP-MODC
SOURCE      synthetic DNA construct
  ORGANISM  synthetic DNA construct
REFERENCE   1  (bases 1 to 11609)
  AUTHORS   Li
  TITLE     Direct Submission
  JOURNAL   Exported Sep 17, 2018 from SnapGene Viewer 4.1.5
            http://www.snapgene.com
FEATURES             Location/Qualifiers
     source          1..11609
                     /organism="synthetic DNA construct"
                     /mol_type="other DNA"
     protein_bind    77..507
                     /label=gypsy insulator
                     /bound_moiety="Su(Hw), CP190, and Mod(mdg4)67.2"
                     /note="chromatin insulator from Drosophila"
     misc_feature    522..643
                     /label=Per
     misc_feature    659..780
                     /label=Per
     misc_feature    807..928
                     /label=Per
     misc_feature    944..1065
                     /label=Per
     promoter        1122..1360
                     /label=hsp70 promoter
                     /note="Drosophila melanogaster hsp70Bb promoter"
     intron          1375..1441
                     /label=Mhc intron
                     /note="modified Drosophila myosin heavy chain intron 16
                     (Pfeiffer et al., 2010)"
     CDS             1467..2345
                     /codon_start=1
                     /label=nls-sfGFP-MODC
                     /translation="MPKKKRKVVSKGEELFTGVVPILVELDGDVNGHKFSVRGEGEGDA
                     TNGKLTLKFICTTGKLPVPWPTLVTTLTYGVQCFSRYPDHMKQHDFFKSAMPEGYVQER
                     TISFKDDGTYKTRAEVKFEGDTLVNRIELKGIDFKEDGNILGHKLEYNFNSHNVYITAD
                     KQKNGIKANFKIRHNVEDGSVQLADHYQQNTPIGDGPVLLPDNHYLSTQSVLSKDPNEK
                     RDHMVLLEFVTAAGITLGMDELYKTSYKKLSHGFPPEVEEQDDGTLPMSCAQESGMDRH
                     PAACASARINV"
     CDS             1470..1490
                     /codon_start=1
                     /product="nuclear localization signal of SV40 (simian virus
                     40) large T antigen"
                     /label=SV40 NLS
                     /translation="PKKKRKV"
```

```
     misc_feature    2363..3038
                     /label=p10
     protein_bind    3044..3474
                     /label=gypsy insulator
                     /bound_moiety="Su(Hw), CP190, and Mod(mdg4)67.2"
                     /note="chromatin insulator from Drosophila"
     misc_feature    8031..8616
                     /label=P element 5' end
     rep_origin      complement(8851..9439)
                     /direction=LEFT
                     /label=ori
                     /note="high-copy-number ColE1/pMB1/pBR322/pUC origin of
                     replication"
     CDS             complement(9610..10470)
                     /codon_start=1
                     /gene="bla"
                     /product="beta-lactamase"
                     /label=AmpR
                     /note="confers resistance to ampicillin, carbenicillin, and
                     related antibiotics"
                     /translation="MSIQHFRVALIPFFAAFCLPVFAHPETLVKVKDAEDQLGARVGYI
                     ELDLNSGKILESFRPEERFPMMSTFKVLLCGAVLSRIDAGQEQLGRRIHYSQNDLVEYS
                     PVTEKHLTDGMTVRELCSAAITMSDNTAANLLLTTIGGPKELTAFLHNMGDHVTRLDRW
                     EPELNEAIPNDERDTTMPVAMATTLRKLLTGELLTLASRQQLIDWMEADKVAGPLLRSA
                     LPAGWFIADKSGAGERGSRGIIAALGPDGKPSRIVVIYTTGSQATMDERNRQIAEIGAS
                     LIKHW"
     promoter        complement(10471..10575)
                     /gene="bla"
                     /label=AmpR promoter
     misc_feature    11377..11609
                     /label=P element 3' end
                     /note="P element 3' end"
ORIGIN
        1 aagcttgggc tgcaggtcga cctcgaggcc tcgagttaac gttacgttaa cgttaacgtt
       61 cgaggtcgac tctagttggc cacgtaataa gtgtgcgttg aatttattcg caaaaacatt
      121 gcatattttc ggcaaagtaa aattttgttg catacettat caaaaaataa gtgctgcata
      181 ctttttagag aaaccaaata attttttatt gcatacccgt tttaataaa atacattgca
      241 taccctcttt taataaaaaa tattgcatac tttgacgaaa caaattttcg ttgcataccc
      301 aataaaagat tattatattg catacccgtt tttaataaaa tacattgcat accctctttt
      361 aataaaraat attgcatacg ttgacgaaac aaattttcgt tgcataccca ataaaagatt
      421 attatattgc atacctttc ttgccatacc atttagccga tcaattgtgc tcggcaacag
      481 tatatttgtg gtgtgccaac caacaactct agactactgc cgagttcgca caactcatgg
      541 tggggcaggg acgggtccga gtgcaaaaag cgtccgagaa accgtaggca gtgaaaagcc
      601 gccgctcacg tggcgaactg cgtgacttgg ccagcaaatc cgcacatggt acctcgacga
      661 gttcgcacaa ctcatggtgg ggcagggacg ggtccgagtg caaaaagcgt ccgagaaacc
      721 gtaggcagtg aaaagccgcc gctcacgtgg cgaactgcgt gacttggcca gcaaatccgc
      781 gcacgaagga tctctagact actgccgagt tcgcacaact catggtgggg cagggacggg
      841 tccgagtgca aaaagcgtcc gagaaaccgt aggcagtgaa aagccgccgc tcacgtggcg
      901 aactgcgtga cttggccagc aaatccgcac atggtacctc gacgagttcg cacaactcat
      961 ggtggggcag ggacgggtcc gagtgcaaaa agcgtccgag aaaccgtagg cagtgaaaag
     1021 ccgccgctca cgtggcgaac tgcgtgactt ggccagcaaa tccgcgcacg agatccagat
     1081 ccactagcga gcggagactc tagcgctagc gacgtcgagc gccggagtat aaatagaggc
     1141 gcttcgtcta cggagcgaca attcaattca aacaagcaaa gtgaacacgt cgctaagcga
     1201 aagctaagca aataaacaag cgcagctgaa caagctaaac aatctgcagt aaagtgcaag
```

1261 ttaaagtgaa tcaattaaaa gtaaccagca accaagtaaa tcaactgcaa ctactgaaat
1321 ctgccaagaa gtaattattg aatacaagaa gagaactctg aatagatcta aaaggtaggt
1381 tcaaccactg atgcctaggc acaccgaaac gactaaccct aattcttatc ctttacttca
1441 ggcggaactt aaaaaaaaaa atcaaaatgc ccaagaagaa gcgcaaggtg gtgtccaagg
1501 gcgaggagct gttcaccggc gtggtgccca tcctggtgga gctggatggc gacgtgaacg
1561 gccacaagtt cagcgtgcgc ggcgagggcg agggcgacgc caccaacggc aagctgaccc
1621 tgaagttcat ctgcaccacc ggcaagctgc ccgtgccctg gcccaccctg gtgaccaccc
1681 tgacctacgg cgtgcagtgc ttcagccgct accccgatca catgaagcag cacgatttct
1741 tcaagagcgc catgcccgag ggctacgtgc aggagcgcac catcagcttc aaggatgacg
1801 gcacctacaa gacccgcgcc gaggtgaagt tcgagggcga taccctggtg aaccgcatcg
1861 agctgaaggg catcgatttc aaggaggatg gcaacatcct gggccacaag ctggagtaca
1921 acttcaacag ccacaacgtg tacatcaccg ccgataagca gaagaacggc atcaaggcca
1981 acttcaagat ccgccacaat gtggaggatg gctccgtgca gctggccgat cactaccagc
2041 agaacacccc catcggcgac ggcccagtgc tgctgcccga taaccactac ctgagcaccc
2101 agagcgtgct gtccaaggac cccaacgaga agcgcgatca catggtgctg ctggagttcg
2161 tgaccgccgc cggcatcacc ctgggcatgg atgagctgta caagactagt tacaagaagc
2221 ttagccatgg cttcccgccg gaggtggagg agcaggatga tggcacgctg cccatgtctt
2281 gtgcccagga gagcgggatg gaccgtcacc ctgcagcctg tgcttctgct aggatcaatg
2341 tgtagatgcg cctagttaat agaatgaatc gtttttaaaa taacaaatca attgttttat
2401 aatattcgta cgattctttg attatgtaat aaaatgtgat cattaggaag attacgaaaa
2461 atataaaaaa tatgagttct gtgtgtataa caaatgctgt aaacgccaca attgtgtttg
2521 ttgcaaataa acccatgatt atttgattaa aattgttgtt ttctttgttc atagacaata
2581 gtgtgttttg cctaaacgtg tactgcataa actccatgcg agtgtatagc gagctagtgg
2641 ctaacgcttg ccccaccaaa gtagattcgt caaaatcctc aatttcatca ccctcctcca
2701 agtttaacat ttggccgtcg gaattaactt ctaaagatgc cacataatct aataaatgaa
2761 atagagattc aaacgtggcg tcatcgtccg tttcgaccat ttccgaaaag aactcgggca
2821 taaactctat gatttctctg gacgtggtgt tgtcgaaact ctcaaagtac gcagtcagga
2881 acgtgcgcga catgtcgtcg ggaaactcgc gcggaaacat gttgttgtaa ccgaacgggt
2941 cccatagcgc caaaaccaaa tctgccagcg tcaatagaat gagcacgatg ccgacaatgg
3001 agctggcttg gatagcgatt cgagttaacg gccggccgct agttggccac gtaataagtg
3061 tgcgttgaat ttattcgcaa aaacattgca tattttcggc aaagtaaaat tttgttgcat
3121 accttatcaa aaaataagtg ctgcatactt tttagagaaa ccaaataatt ttttattgca
3181 tacccgtttt taataaaata cattgcatac cctcttttaa taaaaaatat tgcatacttt
3241 gacgaaacaa attttcgttg catacccaat aaaagattat tatattgcat acccgttttt
3301 aataaaatac attgcatacc ctcttttaat aaaraatatt gcatacgttg acgaaacaaa
3361 ttttcgttgc atacccaata aaagattatt atattgcata cctttttcttg ccataccatt
3421 tagccgatca attgtgctcg gcaacagtat atttgtggtg tgccaaccaa caactctagt
3481 gctgcatcca acgcgttggg agctctccgg atcaattcgg cttcaggtac cgtcgacgat
3541 gtaggtcacg gtctcgaagc cgcggtgcgg gtgccagggc gtgcccttgg gctccccggg
3601 cgcgtactcc acctcaccca tctggtccat catgatgaac gggtcgaggt ggcggtagtt
3661 gatcccggcg aacgcgcggc gcaccgggaa gccctcgccc tcgaaaccgc tgggcgcggt
3721 ggtcacggtg agcacgggac gtgcgacggc gtcggcgggt gcggatacgc ggggcagcgt
3781 cagcgggttc tcgacggtca cggcgggcat gtcgacaagc cgaattgatc cactagaagg
3841 cctaattcgc ggccgcggat ctgatcccgg gcgggtaccg aattctagta tgtatgtaag
3901 ttaataaaac cctttttttgg agaatgtaga tttaaaaaaa catatttttt ttttattttt
3961 tactgcactg gatatcattg aacttatctg atcagtttta aatttacttc gatccaaggg
4021 tatttgaagt accaggttct ttcgattacc tctcactcaa aatgacattc cactcaaagt
4081 cagcgctgtt tgcctccttc tctgtccaca gaaatatcgc cgtctctttc gccgctgcgt
4141 ccgctatctc tttcgccacc gtttgtagcg ttacctagcg tcaatgtccg ccttcagttg
4201 cactttgtca gcggtttcgt gacgaagctc caagcggttt acgccatcaa ttaaacacaa
4261 agtgctgtgc caaaactcct ctcgcttctt atttttgttt gtttttttgag tgattggggt
4321 ggtgattggt tttgggtggg taagcagggg aaagtgtgaa aaatcccggc aatgggccaa
4381 gaggatcagg agctattaat tcgcggaggc agcaaacacc catctgccga gcatctgaac
4441 aatgtgagta gtacatgtgc atacatctta agttcacttg atctatagga actgcgattg

4501 caacatcaaa ttgtctgcgg cgtgagaact gcgacccaca aaaatcccaa accgcaatcg
4561 cacaaacaaa tagtgacacg aaacagatta ttctggtagc tgtgctcgct atataagaca
4621 atttttaaga tcatatcatg atcaagacat ctaaaggcat tcattttcga ctacattctt
4681 ttttacaaaa aatataacaa ccagatattt taagctgatc ctagatgcac aaaaaataaa
4741 taaaagtata aacctacttc gtaggatact tcgttttgtt cggggttaga tgagcataac
4801 gcttgtagtt gatatttgag atcccctatc attgcagggt gacagcggac gcttcgcaga
4861 gctgcattaa ccagggcttc gggcaggcca aaaactacgg cacgctcctg ccacccagtc
4921 cgccggagga ctccggttca gggagcggcc aactagccga gaacctcacc tatgcctggc
4981 acaatatgga catctttggg gcggtcaatc agccgggctc cggatggcgg cagctggtca
5041 accggacacg cggactattc tgcaacgagc gacacatacc ggcgcccagg aaacatttgc
5101 tcaagaacgg tgagtttcta ttcgcagtcg gctgatctgt gtgaaatctt aataaagggt
5161 ccaattacca atttgaaact cagtttgcgg cgtggcctat ccgggcgaac ttttggccgt
5221 gatgggcagt tccggtgccg gaaagacgac cctgctgaat gcccttgcct ttcgatcgcc
5281 gcagggcatc caagtatcgc catccgggat gcgactgctc aatggccaac ctgtggacgc
5341 caaggagatg caggccaggt gcgcctatgt ccagcaggat gacctcttta tcggctccct
5401 aacggccagg gaacacctga ttttccaggc catggtgcgg atgccacgac atctgaccta
5461 tcggcagcga gtggcccgcg tggatcaggt gatccaggag ctttcgctca gcaaatgtca
5521 gcacacgatc atcggtgtgc ccggcagggt gaaaggtctg tccggcggag aaaggaagcg
5581 tctggcattc gcctccgagg cactaaccga tccgccgctt ctgatctgcg atgagcccac
5641 ctccggactg gactcattta ccgcccacag cgtcgtccag gtgctgaaga agctgtcgca
5701 gaagggcaag accgtcatcc tgaccattca tcagccgtct tccgagctgt ttgagctctt
5761 tgacaagatc cttctgatgg ccgagggcag ggtagctttc ttgggcactc ccagcgaagc
5821 cgtcgacttc ttttcctagt gagttcgatg tgtttattaa gggtatctag cattacatta
5881 catctcaact cctatccagc gtgggtgccc agtgtcctac caactacaat ccggcggact
5941 tttacgtaca ggtgttggcc gttgtgcccg gacgggagat cgagtcccgt gatcggatcg
6001 ccaagatatg gcgacaattt tgctattagc aaagtagccc gggatatgga gcagttgttg
6061 gccaccaaaa atttggagaa gccactggag cagccggaga atgggtacac ctacaaggcc
6121 acctggttca tgcagttccg ggcggtcctg tggcgatcct ggctgtcggt gctcaaggaa
6181 ccactcctcg taaaagtgcg acttattcag acaacggtga gtggttccag tggaaacaaa
6241 tgatataacg cttacaattc ttggaaacaa attcgctaga ttttagttag aattgcctga
6301 ttccacaccc ttcttagttt ttttcaatga gatgtatagt ttatagtttt gcagaaaata
6361 aataaatttc atttaactcg cgaacatgtt gaagatatga atattaatga gatgcgagta
6421 acattttaat ttgcagatgg ttgccatctt gattggcctc atctttttgg gccaacaact
6481 cacgcaagtg ggcgtgatga atatcaacgg agccatcttc ctcttcctga ccaacatgac
6541 ctttcaaaac gtctttgcca cgataaatgt aagtcttgtt tagaatacat ttgcatatta
6601 ataatttact aactttctaa tgaatcgatt cgatttaggt gttcacctca gagctgccag
6661 tttttatgag ggaggcccga agtcgacttt atcgctgtga cacatacttt ctgggcaaaa
6721 cgattgccga attaccgctt tttctcacag tgccactggt cttcacggcg attgcctatc
6781 cgatgatcgg actgcgggcc ggagtgctgc acttcttcaa ctgcctggcg ctggtcactc
6841 tggtggccaa tgtgtcaacg tccttcggat atctaatatc ctgcgccagc tcctcgacct
6901 cgatggcgct gtctgtgggt ccgccggtta tcataccatt cctgctcttt ggcggcttct
6961 tcttgaactc gggctcggtg ccagtatacc tcaaatggtt gtcgtacctc tcatggttcc
7021 gttacgccaa cgagggtctg ctgattaacc aatgggcgga cgtggagccg ggcgaaatta
7081 gctgcacatc gtcgaacacc acgtgcccca gttcgggcaa ggtcatcctg gagacgctta
7141 acttctccgc cgccgatctg ccgctggact acgtgggtct ggccattctc atcgtgagct
7201 tccgggtgct cgcatatctg gctctaagac ttcgggcccg acgcaaggag tagccgacat
7261 atatccgaaa taactgcttg tttttttttt taccattatt accatcgtgt ttactgttta
7321 ttgccccctc aaaaagctaa tgtaattata tttgtgccaa taaaaacaag atatgaccta
7381 tagaatacaa gtatttcccc ttcgaacatc cccacaagta gactttggat ttgtcttcta
7441 accaaaagac ttacacacct gcataccttа catcaaaaac tcgtttatcg ctacataaaa
7501 caccgggata tattttttat atacatactt ttcaaatcgc gcgccctctt cataattcac
7561 ctccaccaca ccacgtttcg tagttgctct ttcgctgtct cccacccgct ctccgcaaca
7621 cattcacctt ttgttcgacg accttggagc gactgtcgtt agttccgcgc gattcggttc
7681 gctcaaatgg ttccgagtgg ttcatttcgt ctcaatagaa attagtaata aatatttgta

7741 tgtacaattt atttgctcca atatatttgt atatatttcc ctcacagcta tatttattct
7801 aatttaatat tatgactttt taaggtaatt ttttgtgacc tgttcggagt gattagcgtt
7861 acaatttgaa ctgaaagtga catccagtgt ttgttccttg tgtagatgca tctcaaaaaa
7921 atggtgggca taatagtgtt gtttatatat atcaaaaata acaactataa taataagaat
7981 acatttaatt tagaaaatgc ttggatttca ctggaactag aattaattcg gctgctgctc
8041 taaacgacgc atttcgtact ccaaagtacg aatttttttcc ctcaagctct tattttcatt
8101 aaacaatgaa caggacctaa cgcacagtca cgttattgtt tacataaatg atttttttta
8161 ctattcaaac ttactctgtt tgtgtactcc cactggtata gccttctttt atcttttctg
8221 gttcaggctc tatcacttta ctaggtacgg catctgcgtt gagtcgcctc cttttaaatg
8281 tctgaccttt tgcaggtgca gccttccact gcgaatcttt aaagtgggta tcacaaattt
8341 gggagttttc accaaggctg cacccaaggc tctgctccca caattttctc ttaatagcac
8401 acttcggcac gtgaattaat tttactccag tcacagcttt gcagcaaaat ttgcaatatt
8461 tcattttttt ttattccacg taagggttaa tgttttcaaa aaaaaattcg tccgcacaca
8521 acctttcctc tcaacaagca aacgtgcact gaatttaagt gtatacttcg gtaagcttcg
8581 gctatcgacg ggaccacctt atgttatttc atcatgggcc agacccacgt agtccagcgg
8641 cagatcggcg gcggagaagt taagcgtctc caggatgacc ttgcccgaac tggggcacgt
8701 ggtgttcgac gatgtgcagc taatttcgcc cggctccacg tccgcccatt ggttaatcag
8761 cagaccctcg ttggcgtaac ggaaccatga gaggtacgac aaccatttga ggtatactgg
8821 caccgagccc gagttcaaga agaaggcgtt tttccatagg ctccgccccc ctgacgagca
8881 tcacaaaaat cgacgctcaa gtcagaggtg gcgaaacccg acaggactat aaagatacca
8941 ggcgtttccc cctggaagct ccctcgtgcg ctctcctgtt ccgaccctgc cgcttaccgg
9001 atacctgtcc gcctttctcc cttcgggaag cgtggcgctt tctcaatgct cacgctgtag
9061 gtatctcagt tcggtgtagg tcgttcgctc caagctgggc tgtgtgcacg aaccccccgt
9121 tcagcccgac cgctgcgcct tatccggtaa ctatcgtctt gagtccaacc cggtaagaca
9181 cgacttatcg ccactggcag cagccactgg taacaggatt agcagagcga ggtatgtagg
9241 cggtgctaca gagttcttga agtggtggcc taactacggc tacactagaa ggacagtatt
9301 tggtatctgc gctctgctga agccagttac cttcggaaaa agagttggta gctcttgatc
9361 cggcaaacaa accaccgctg gtagcggtgg tttttttgtt tgcaagcagc agattacgcg
9421 cagaaaaaaa ggatctcaag aagatccttt gatcttttct acggggtctg acgctcagtg
9481 gaacgaaaac tcacgttaag ggattttggt catgagatta tcaaaaagga tcttcaccta
9541 gatcctttta aattaaaaat gaagttttaa atcaatctaa agtatatatg agtaaacttg
9601 gtctgacagt taccaatgct taatcagtga ggcacctatc tcagcgatct gtctatttcg
9661 ttcatccata gttgcctgac tccccgtcgt gtagataact acgatacggg agggcttacc
9721 atctggcccc agtgctgcaa tgataccgcg agacccacgc tcaccggctc cagatttatc
9781 agcaataaac cagccagccg gaagggccga gcgcagaagt ggtcctgcaa ctttatccgc
9841 ctccatccag tctattaatt gttgccggga agctagagta agtagttcgc cagttaatag
9901 tttgcgcaac gttgttgcca ttgctacagg catcgtggtg tcacgctcgt cgtttggtat
9961 ggcttcattc agctccggtt cccaacgatc aaggcgagtt acatgatccc ccatgttgtg
10021 caaaaaagcg gttagctcct tcggtcctcc gatcgttgtc agaagtaagt tggccgcagt
10081 gttatcactc atggttatgg cagcactgca taattctctt actgtcatgc catccgtaag
10141 atgcttttct gtgactggtg agtactcaac caagtcattc tgagaatagt gtatgcggcg
10201 accgagttgc tcttgcccgg cgtcaatacg ggataatacc gcgccacata gcagaacttt
10261 aaaagtgctc atcattggaa aacgttcttc ggggcgaaaa ctctcaagga tcttaccgct
10321 gttgagatcc agttcgatgt aacccactcg tgcacccaac tgatcttcag catcttttac
10381 tttcaccagc gtttctgggt gagcaaaaac aggaaggcaa aatgccgcaa aaaagggaat
10441 aagggcgaca cggaaatgtt gaatactcat actcttcctt tttcaatatt attgaagcat
10501 ttatcagggt tattgtctca tgagcggata catatttgaa tgtatttaga aaaataaaca
10561 aataggggtt ccgcgcacat ttccccgaaa agtgccacct gacgtctaag aaaccattat
10621 tatcatgaca ttaacctata aaaataggcg tatcacgagg ccctttcgtc tcgcgcgttt
10681 cggtgatgac ggtgaaaacc tctgacacat gcagctcccg gagacggtca cagcttgtct
10741 gtaagcggat gccgggagca gacaagcccg tcagggcgcg tcagcgggtg ttggcgggtg
10801 tcggggctgg cttaactatg cggcatcaga gcagattgta ctgagagtgc accatatgcg
10861 gtgtgaaata ccgcaccgaa tcgcgcggaa ctaacgacag tcgctccaag gtcgtcgaac
10921 aaaaggtgaa tgtgttgcgg agagcgggtg ggagacagcg aaagagcaac tacgaaacgt

```
10981 ggtgtggtgg aggtgaatta tgaagagggc gcgcgatttg aaaagtatgt atataaaaaa
11041 tatatcccgg tgttttatgt agcgataaac gagtttttga tgtaaggtat gcaggtgtgt
11101 aagtcttttg gttagaagac aaatccaaag tctacttgtg gggatgttcg aaggggaaat
11161 acttgtattc tataggtcat atcttgtttt tattggcaca aatataatta cattagcttt
11221 ttgagggggc aataaacagt aaacacgatg gtaataatgg taaaaaaaaa aacaagcagt
11281 tatttcggat atatgtcggc tactccttgc gtcgggcccg aagtcttaga gccagatatg
11341 cgagcacccg gaagctcacg atgagaatgg ccagaccatg atgaaataac ataaggtggt
11401 cccgtcggca agagacatcc acttaacgta tgcttgcaat aagtgcgagt gaaaggaata
11461 gtattctgag tgtcgtattg agtctgagtg agacagcgat atgattgttg attaaccctt
11521 agcatgtccg tggggtttga attaactcat aatattaatt agacgaaatt atttttaaag
11581 ttttattttt aataatttgc gagtacgca
//
```

**B) 4xTIM Sequence**

```
LOCUS       Exported               11816 bp ds-DNA     circular SYN 17-SEP-2018
DEFINITION  synthetic circular DNA
ACCESSION   .
VERSION     .
KEYWORDS    4XTim nls-sfGFP-MODC
SOURCE      synthetic DNA construct
  ORGANISM  synthetic DNA construct
REFERENCE   1  (bases 1 to 11816)
  AUTHORS   Li
  TITLE     Direct Submission
  JOURNAL   Exported Sep 17, 2018 from SnapGene Viewer 4.1.5
            http://www.snapgene.com
FEATURES             Location/Qualifiers
     source          1..11816
                     /organism="synthetic DNA construct"
                     /mol_type="other DNA"
     protein_bind    77..507
                     /label=gypsy insulator
                     /bound_moiety="Su(Hw), CP190, and Mod(mdg4)67.2"
                     /note="chromatin insulator from Drosophila"
     misc_feature    521..694
                     /label=Tim
     misc_feature    709..882
                     /label=Tim
     misc_feature    904..1077
                     /label=Tim
     misc_feature    1092..1265
                     /label=Tim
     promoter        1329..1567
                     /label=hsp70 promoter
                     /note="Drosophila melanogaster hsp70Bb promoter"
     intron          1582..1648
                     /label=Mhc intron
                     /note="modified Drosophila myosin heavy chain intron 16
                     (Pfeiffer et al., 2010)"
     CDS             1674..2552
                     /codon_start=1
                     /label=nls-GFP-MODC
                     /translation="MPKKKRKVVSKGEELFTGVVPILVELDGDVNGHKFSVRGEGEGDA
                     TNGKLTLKFICTTGKLPVPWPTLVTTLTYGVQCFSRYPDHMKQHDFFKSAMPEGYVQER
                     TISFKDDGTYKTRAEVKFEGDTLVNRIELKGIDFKEDGNILGHKLEYNFNSHNVYITAD
                     KQKNGIKANFKIRHNVEDGSVQLADHYQQNTPIGDGPVLLPDNHYLSTQSVLSKDPNEK
                     RDHMVLLEFVTAAGITLGMDELYKTSYKKLSHGFPPEVEEQDDGTLPMSCAQESGMDRH
                     PAACASARINV"
     CDS             1677..1697
                     /codon_start=1
                     /product="nuclear localization signal of SV40 (simian virus
                     40) large T antigen"
                     /label=SV40 NLS
                     /translation="PKKKRKV"
     misc_feature    2570..3245
                     /label=p10
```

```
     protein_bind    3251..3681
                     /label=gypsy insulator
                     /bound_moiety="Su(Hw), CP190, and Mod(mdg4)67.2"
                     /note="chromatin insulator from Drosophila"
     misc_feature    8238..8823
                     /label=P element 5' end
     rep_origin      complement(9058..9646)
                     /direction=LEFT
                     /label=ori
                     /note="high-copy-number ColE1/pMB1/pBR322/pUC origin of
                     replication"
     CDS             complement(9817..10677)
                     /codon_start=1
                     /gene="bla"
                     /product="beta-lactamase"
                     /label=AmpR
                     /note="confers resistance to ampicillin, carbenicillin, and
                     related antibiotics"
                     /translation="MSIQHFRVALIPFFAAFCLPVFAHPETLVKVKDAEDQLGARVGYI
                     ELDLNSGKILESFRPEERFPMMSTFKVLLCGAVLSRIDAGQEQLGRRIHYSQNDLVEYS
                     PVTEKHLTDGMTVRELCSAAITMSDNTAANLLLTTIGGPKELTAFLHNMGDHVTRLDRW
                     EPELNEAIPNDERDTTMPVAMATTLRKLLTGELLTLASRQQLIDWMEADKVAGPLLRSA
                     LPAGWFIADKSGAGERGSRGIIAALGPDGKPSRIVVIYTTGSQATMDERNRQIAEIGAS
                     LIKHW"
     promoter        complement(10678..10782)
                     /gene="bla"
                     /label=AmpR promoter
     misc_feature    11584..11816
                     /label=P element 3' end
                     /note="P element 3' end"
ORIGIN
        1 aagcttgggc tgcaggtcga cctcgaggcc tcgagttaac gttacgttaa cgttaacgtt
       61 cgaggtcgac tctagttggc cacgtaataa gtgtgcgttg aatttattcg caaaaacatt
      121 gcatattttc ggcaaagtaa aattttgttg cataccttat caaaaaataa gtgctgcata
      181 ctttttagag aaaccaaata attttttatt gcatacccgt ttttaataaa atacattgca
      241 taccctcttt taataaaaaa tattgcatac tttgacgaaa caaattttcg ttgcataccc
      301 aataaaagat tattatattg catacccgtt tttaataaaa tacattgcat accctctttt
      361 aataaaraat attgcatacg ttgacgaaac aaattttcgt tgcataccca ataaaagatt
      421 attatattgc ataccttttc ttgccatacc atttagccga tcaattgtgc tcggcaacag
      481 tatatttgtg gtgtgccaac caacaactct agactactgc ttccggcgct ggttattcat
      541 gttatcgaac atcgtagtgg cggttggcaa ataaacgtgc ggcacgttgt gattacacgt
      601 gagccgattt ccccggccgt ccggcattga gtgcgagcgg gacggagcag cgcggcgaga
      661 gctttcggct agcgtctttc gttcgcacaa acgcacatgg tacctcgatt ccggcgctgg
      721 ttattcatgt tatcgaacat cgtagtggcg gttggcaaat aaacgtgcgg cacgttgtga
      781 ttacacgtga gccgatttcc ccggccgtcc ggcattgagt gcgagcggga cggagcagcg
      841 cggcgagagc tttcggctag cgtctttcgt tcgcacaaac gcgcacgatc tagagactac
      901 tgcttccggc gctggttatt catgttatcg aacatcgtag tggcggttgg caaataaacg
      961 tgcggcacgt tgtgattaca cgtgagccga tttccccggc cgtccggcat tgagtgcgag
     1021 cgggacggag cagcgcggcg agagctttcg gctagcgtct ttcgttcgca caaacgcaca
     1081 tggtacctcg attccggcgc tggttattca tgttatcgaa catcgtagtg gcggttggca
     1141 aataaacgtg cggcacgttg tgattacacg tgagccgatt tccccggccg tccggcattg
     1201 agtgcgagcg ggacggagca gcgcggcgag agctttcggc tagcgtcttt cgttcgcaca
     1261 aacgcgcacg atctagagga tccagatcca ctagcgagcg gagactctag cgctagcgac
     1321 gtcgagcgcc ggagtataaa tagaggcgct tcgtctacgg agcgacaatt caattcaaac
```

1381 aagcaaagtg aacacgtcgc taagcgaaag ctaagcaaat aaacaagcgc agctgaacaa
1441 gctaaacaat ctgcagtaaa gtgcaagtta aagtgaatca attaaaagta accagcaacc
1501 aagtaaatca actgcaacta ctgaaatctg ccaagaagta attattgaat acaagaagag
1561 aactctgaat agatctaaaa ggtaggttca accactgatg cctaggcaca ccgaaacgac
1621 taaccctaat tcttatcctt tacttcaggc ggaacttaaa aaaaaaaatc aaaatgccca
1681 agaagaagcg caaggtggtg tccaagggcg aggagctgtt caccggcgtg gtgcccatcc
1741 tggtggagct ggatggcgac gtgaacggcc acaagttcag cgtgcgcggc gagggcgagg
1801 gcgacgccac caacggcaag ctgaccctga agttcatctg caccaccggc aagctgcccg
1861 tgccctggcc caccctggtg accaccctga cctacggcgt gcagtgcttc agccgctacc
1921 ccgatcacat gaagcagcac gatttcttca agagcgccat gcccgagggc tacgtgcagg
1981 agcgcaccat cagcttcaag gatgacggca cctacaagac ccgcgccgag gtgaagttcg
2041 agggcgatac cctggtgaac cgcatcgagc tgaagggcat cgatttcaag gaggatggca
2101 acatcctggg ccacaagctg gagtacaact tcaacagcca caacgtgtac atcaccgccg
2161 ataagcagaa gaacggcatc aaggccaact tcaagatccg ccacaatgtg gaggatggct
2221 ccgtgcagct ggccgatcac taccagcaga acacccccat cggcgacggc ccagtgctgc
2281 tgcccgataa ccactacctg agcacccaga gcgtgctgtc caaggacccc aacgagaagc
2341 gcgatcacat ggtgctgctg gagttcgtga ccgccgccgg catcaccctg ggcatggatg
2401 agctgtacaa gactagttac aagaagctta gccatggctt cccgccggag gtggaggagc
2461 aggatgatgg cacgctgccc atgtcttgtg cccaggagag cgggatggac cgtcaccctg
2521 cagcctgtgc ttctgctagg atcaatgtgt agatgcgcct agttaataga atgaatcgtt
2581 tttaaaataa caaatcaatt gttttataat attcgtacga ttctttgatt atgtaataaa
2641 atgtgatcat taggaagatt acgaaaaata taaaaaatat gagttctgtg tgtataacaa
2701 atgctgtaaa cgccacaatt gtgtttgttg caaataaacc catgattatt tgattaaaat
2761 tgttgttttc tttgttcata gacaatagtg tgttttgcct aaacgtgtac tgcataaact
2821 ccatgcgagt gtatagcgag ctagtggcta acgcttgccc caccaaagta gattcgtcaa
2881 aatcctcaat ttcatcaccc tcctccaagt ttaacatttg gccgtcggaa ttaacttcta
2941 aagatgccac ataatctaat aaatgaaata gagattcaaa cgtggcgtca tcgtccgttt
3001 cgaccatttc cgaaaagaac tcgggcataa actctatgat ttctctggac gtggtgttgt
3061 cgaaactctc aaagtacgca gtcaggaacg tgcgcgacat gtcgtcggga aactcgcgcg
3121 gaaacatgtt gttgtaaccg aacgggtccc atagcgccaa aaccaaatct gccagcgtca
3181 atagaatgag cacgatgccg acaatggagc tggcttggat agcgattcga gttaacggcc
3241 ggccgctagt tggccacgta ataagtgtgc gttgaattta ttcgcaaaaa cattgcatat
3301 tttcggcaaa gtaaaatttt gttgcatacc ttatcaaaaa ataagtgctg catacttttt
3361 agagaaacca aataattttt tattgcatac ccgtttttaa taaaatacat tgcataccct
3421 cttttaataa aaaatattgc atactttgac gaaacaaatt ttcgttgcat acccaataaa
3481 agattattat attgcatacc cgtttttaat aaaatacatt gcataccctc ttttaataaa
3541 raatattgca tacgttgacg aaacaaattt tcgttgcata cccaataaaa gattattata
3601 ttgcatacct tttcttgcca taccatttag ccgatcaatt gtgctcggca acagtatatt
3661 tgtggtgtgc caaccaacaa ctctagtgct gcatccaacg cgttgggagc tctccggatc
3721 aattcggctt caggtaccgt cgacgatgta ggtcacggtc tcgaagccgc ggtgcgggtg
3781 ccagggcgtg cccttgggct ccccgggcgc gtactccacc tcacccatct ggtccatcat
3841 gatgaacggg tcgaggtggc ggtagttgat cccggcgaac gcgcggcgca ccgggaagcc
3901 ctcgccctcg aaaccgctgg gcgcggtggt cacggtgagc acgggacgtg cgacggcgtc
3961 ggcgggtgcg gatacgcggg gcagcgtcag cgggttctcg acggtcacgg cgggcatgtc
4021 gacaagccga attgatccac tagaaggcct aattcgcggc cgcggatctg atcccgggcg
4081 ggtaccgaat tctagtatgt atgtaagtta ataaaaccct tttttggaga atgtagattt
4141 aaaaaaacat attttttttt tattttttac tgcactggat atcattgaac ttatctgatc
4201 agttttaaat ttacttcgat ccaagggtat ttgaagtacc aggttctttc gattacctct
4261 cactcaaaat gacattccac tcaaagtcag cgctgtttgc ctccttctct gtccacagaa
4321 atatcgccgt ctctttcgcc gctgcgtccg ctatctcttt cgccaccgtt tgtagcgtta
4381 cctagcgtca atgtccgcct tcagttgcac tttgtcagcg gtttcgtgac gaagctccaa
4441 gcggtttacg ccatcaatta aacacaaagt gctgtgccaa aactcctctc gcttcttatt
4501 tttgtttgtt ttttgagtga ttggggtggt gattggtttt gggtgggtaa gcaggggaaa
4561 gtgtgaaaaa tcccggcaat gggccaagag gatcaggagc tattaattcg cggaggcagc

4621 aaacacccat ctgccgagca tctgaacaat gtgagtagta catgtgcata catcttaagt
4681 tcacttgatc tataggaact gcgattgcaa catcaaattg tctgcggcgt gagaactgcg
4741 acccacaaaa atcccaaacc gcaatcgcac aaacaaatag tgacacgaaa cagattattc
4801 tggtagctgt gctcgctata taagacaatt tttaagatca tatcatgatc aagacatcta
4861 aaggcattca ttttcgacta cattcttttt tacaaaaaat ataacaacca gatattttaa
4921 gctgatccta gatgcacaaa aaataaataa aagtataaac ctacttcgta ggatacttcg
4981 ttttgttcgg ggttagatga gcataacgct tgtagttgat atttgagatc ccctatcatt
5041 gcagggtgac agcggacgct tcgcagagct gcattaacca gggcttcggg caggccaaaa
5101 actacggcac gctcctgcca cccagtccgc cggaggactc cggttcaggg agcggccaac
5161 tagccgagaa cctcacctat gcctggcaca atatggacat ctttggggcg gtcaatcagc
5221 cgggctccgg atggcggcag ctggtcaacc ggacacgcgg actattctgc aacgagcgac
5281 acataccggc gcccaggaaa catttgctca agaacggtga gtttctattc gcagtcggct
5341 gatctgtgtg aaatcttaat aaagggtcca attaccaatt tgaaactcag tttgcggcgt
5401 ggcctatccg ggcgaacttt tggccgtgat gggcagttcc ggtgccggaa agacgaccct
5461 gctgaatgcc cttgcctttc gatcgccgca gggcatccaa gtatcgccat ccgggatgcg
5521 actgctcaat ggccaacctg tggacgccaa ggagatgcag gccaggtgcg cctatgtcca
5581 gcaggatgac ctctttatcg gctccctaac ggccagggaa cacctgattt tccaggccat
5641 ggtgcggatg ccacgacatc tgacctatcg gcagcgagtg gcccgcgtgg atcaggtgat
5701 ccaggagctt tcgctcagca aatgtcagca cacgatcatc ggtgtgcccg gcagggtgaa
5761 aggtctgtcc ggcggagaaa ggaagcgtct ggcattcgcc tccgaggcac taaccgatcc
5821 gccgcttctg atctgcgatg agcccacctc cggactggac tcatttaccg cccacagcgt
5881 cgtccaggtg ctgaagaagc tgtcgcagaa gggcaagacc gtcatcctga ccattcatca
5941 gccgtcttcc gagctgtttg agctctttga caagatcctt ctgatggccg agggcagggt
6001 agctttcttg ggcactccca gcgaagccgt cgacttcttt tcctagtgag ttcgatgtgt
6061 ttattaaggg tatctagcat tacattacat ctcaactcct atccagcgtg ggtgcccagt
6121 gtcctaccaa ctacaatccg gcggactttt acgtacaggt gttggccgtt gtgcccggac
6181 gggagatcga gtcccgtgat cggatcgcca agatatggcg acaattttgc tattagcaaa
6241 gtagcccggg atatggagca gttgttggcc accaaaaatt tggagaagcc actggagcag
6301 ccggagaatg ggtacaccta caaggccacc tggttcatgc agttccgggc ggtcctgtgg
6361 cgatcctggc tgtcggtgct caaggaacca ctcctcgtaa aagtgcgact tattcagaca
6421 acggtgagtg gttccagtgg aaacaaatga tataacgctt acaattcttg gaaacaaatt
6481 cgctagattt tagttagaat tgcctgattc cacacccttc ttagtttttt tcaatgagat
6541 gtatagttta tagttttgca gaaaataaat aaatttcatt taactcgcga acatgttgaa
6601 gatatgaata ttaatgagat gcgagtaaca ttttaatttg cagatggttg ccatcttgat
6661 tggcctcatc tttttgggcc aacaactcac gcaagtgggc gtgatgaata tcaacggagc
6721 catcttcctc ttcctgacca acatgacctt tcaaaacgtc tttgccacga taaatgtaag
6781 tcttgtttag aatacatttg catattaata atttactaac tttctaatga atcgattcga
6841 tttaggtgtt cacctcagag ctgccagttt ttatgaggga ggcccgaagt cgactttatc
6901 gctgtgacac atactttctg ggcaaaacga ttgccgaatt accgcttttt ctcacagtgc
6961 cactggtctt cacggcgatt gcctatccga tgatcggact gcgggccgga gtgctgcact
7021 tcttcaactg cctggcgctg gtcactctgg tggccaatgt gtcaacgtcc ttcggatatc
7081 taatatcctg cgccagctcc tcgacctcga tggcgctgtc tgtgggtccg ccggttatca
7141 taccattcct gctctttggc ggcttcttct tgaactcggg ctcggtgcca gtatacctca
7201 aatggttgtc gtacctctca tggttccgtt acgccaacga gggtctgctg attaaccaat
7261 gggcggacgt ggagccgggc gaaattagct gcacatcgtc gaacaccacg tgccccagtt
7321 cgggcaaggt catcctggag acgcttaact tctccgccgc cgatctgccg ctggactacg
7381 tgggtctggc cattctcatc gtgagcttcc gggtgctcgc atatctggct ctaagacttc
7441 gggcccgacg caaggagtag ccgacatata tccgaaataa ctgcttgttt ttttttttac
7501 cattattacc atcgtgttta ctgtttattg ccccctcaaa aagctaatgt aattatattt
7561 gtgccaataa aaacaagata tgacctatag aatacaagta tttccccttc gaacatcccc
7621 acaagtagac tttggatttg tcttctaacc aaaagactta cacacctgca taccttacat
7681 caaaaactcg tttatcgcta cataaaacac cgggatatat tttttatata catacttttc
7741 aaatcgcgcg ccctcttcat aattcacctc caccacacca cgtttcgtag ttgctctttc
7801 gctgtctccc acccgctctc cgcaacacat tcacctttttg ttcgacgacc ttggagcgac

7861 tgtcgttagt tccgcgcgat tcggttcgct caaatggttc cgagtggttc atttcgtctc
7921 aatagaaatt agtaataaat atttgtatgt acaatttatt tgctccaata tatttgtata
7981 tatttccctc acagctatat ttattctaat ttaatattat gactttttaa ggtaattttt
8041 tgtgacctgt tcggagtgat tagcgttaca atttgaactg aaagtgacat ccagtgtttg
8101 ttccttgtgt agatgcatct caaaaaaatg gtgggcataa tagtgttgtt tatatatatc
8161 aaaaataaca actataataa taagaataca tttaatttag aaaatgcttg gatttcactg
8221 gaactagaat taattcggct gctgctctaa acgacgcatt tcgtactcca aagtacgaat
8281 tttttccctc aagctcttat tttcattaaa caatgaacag gacctaacgc acagtcacgt
8341 tattgtttac ataaatgatt tttttacta ttcaaactta ctctgtttgt gtactcccac
8401 tggtatagcc ttcttttatc ttttctggtt caggctctat cactttacta ggtacggcat
8461 ctgcgttgag tcgcctcctt ttaaatgtct gacctttttgc aggtgcagcc ttccactgcg
8521 aatctttaaa gtgggtatca caaatttggg agttttcacc aaggctgcac ccaaggctct
8581 gctcccacaa ttttctctta atagcacact tcggcacgtg aattaatttt actccagtca
8641 cagctttgca gcaaaatttg caatatttca ttttttttta ttccacgtaa gggttaatgt
8701 tttcaaaaaa aaattcgtcc gcacacaacc tttcctctca acaagcaaac gtgcactgaa
8761 tttaagtgta tacttcggta agcttcggct atcgacggga ccaccttatg ttatttcatc
8821 atgggccaga cccacgtagt ccagcggcag atcggcggcg gagaagttaa gcgtctccag
8881 gatgaccttg cccgaactgg ggcacgtggt gttcgacgat gtgcagctaa tttcgcccgg
8941 ctccacgtcc gcccattggt taatcagcag accctcgttg gcgtaacgga accatgagag
9001 gtacgacaac catttgaggt atactggcac cgagcccgag ttcaagaaga aggcgttttt
9061 ccataggctc cgcccccctg acgagcatca caaaaatcga cgctcaagtc agaggtggcg
9121 aaacccgaca ggactataaa gataccaggc gtttccccct ggaagctccc tcgtgcgctc
9181 tcctgttccg accctgccgc ttaccggata cctgtccgcc tttctccctt cgggaagcgt
9241 ggcgctttct caatgctcac gctgtaggta tctcagttcg gtgtaggtcg ttcgctccaa
9301 gctgggctgt gtgcacgaac cccccgttca gcccgaccgc tgcgccttat ccggtaacta
9361 tcgtcttgag tccaacccgg taagacacga cttatcgcca ctggcagcag ccactggtaa
9421 caggattagc agagcgaggt atgtaggcgg tgctacagag ttcttgaagt ggtggcctaa
9481 ctacggctac actagaagga cagtatttgg tatctgcgct ctgctgaagc cagttacctt
9541 cggaaaaaga gttggtagct cttgatccgg caaacaaacc accgctggta gcggtggttt
9601 ttttgtttgc aagcagcaga ttacgcgcag aaaaaaagga tctcaagaag atcctttgat
9661 cttttctacg gggtctgacg ctcagtggaa cgaaaactca cgttaaggga ttttggtcat
9721 gagattatca aaaaggatct tcacctagat ccttttaaat taaaaatgaa gttttaaatc
9781 aatctaaagt atatatgagt aaacttggtc tgacagttac caatgcttaa tcagtgaggc
9841 acctatctca gcgatctgtc tatttcgttc atccatagtt gcctgactcc ccgtcgtgta
9901 gataactacg atacgggagg gcttaccatc tggccccagt gctgcaatga taccgcgaga
9961 cccacgctca ccggctccag atttatcagc aataaaccag ccagccggaa gggccgagcg
10021 cagaagtggt cctgcaactt tatccgcctc catccagtct attaattgtt gccgggaagc
10081 tagagtaagt agttcgccag ttaatagttt gcgcaacgtt gttgccattg ctacaggcat
10141 cgtggtgtca cgctcgtcgt ttggtatggc ttcattcagc tccggttccc aacgatcaag
10201 gcgagttaca tgatccccca tgttgtgcaa aaaagcggtt agctccttcg gtcctccgat
10261 cgttgtcaga agtaagttgg ccgcagtgtt atcactcatg gttatggcag cactgcataa
10321 ttctcttact gtcatgccat ccgtaagatg cttttctgtg actggtgagt actcaaccaa
10381 gtcattctga gaatagtgta tgcggcgacc gagttgctct tgcccggcgt caatacggga
10441 taataccgcg ccacatagca gaactttaaa agtgctcatc attggaaaac gttcttcggg
10501 gcgaaaactc tcaaggatct taccgctgtt gagatccagt tcgatgtaac ccactcgtgc
10561 acccaactga tcttcagcat cttttacttt caccagcgtt tctgggtgag caaaaacagg
10621 aaggcaaaat gccgcaaaaa agggaataag ggcgacacgg aaatgttgaa tactcatact
10681 cttccttttt caatattatt gaagcattta tcagggttat tgtctcatga gcggatacat
10741 atttgaatgt atttagaaaa ataaacaaat aggggttccg cgcacatttc cccgaaaagt
10801 gccacctgac gtctaagaaa ccattattat catgacatta acctataaaa ataggcgtat
10861 cacgaggccc tttcgtctcg cgcgtttcgg tgatgacggt gaaaacctct gacacatgca
10921 gctcccggag acggtcacag cttgtctgta agcggatgcc gggagcagac aagcccgtca
10981 gggcgcgtca gcgggtgttg gcgggtgtcg gggctggctt aactatgcgg catcagagca
11041 gattgtactg agagtgcacc atatgcggtg tgaaataccg caccgaatcg cgcggaacta

```
11101 acgacagtcg ctccaaggtc gtcgaacaaa aggtgaatgt gttgcggaga gcgggtggga
11161 gacagcgaaa gagcaactac gaaacgtggt gtggtggagg tgaattatga agagggcgcg
11221 cgatttgaaa agtatgtata taaaaaatat atcccggtgt tttatgtagc gataaacgag
11281 tttttgatgt aaggtatgca ggtgtgtaag tcttttggtt agaagacaaa tccaaagtct
11341 acttgtgggg atgttcgaag gggaaatact tgtattctat aggtcatatc ttgtttttat
11401 tggcacaaat ataattacat tagctttttg agggggcaat aaacagtaaa cacgatggta
11461 ataatggtaa aaaaaaaaac aagcagttat ttcggatata tgtcggctac tccttgcgtc
11521 gggcccgaag tcttagagcc agatatgcga gcacccggaa gctcacgatg agaatggcca
11581 gaccatgatg aaataacata aggtggtccc gtcggcaaga gacatccact taacgtatgc
11641 ttgcaataag tgcgagtgaa aggaatagta ttctgagtgt cgtattgagt ctgagtgaga
11701 cagcgatatg attgttgatt aacccttagc atgtccgtgg ggtttgaatt aactcataat
11761 attaattaga cgaaattatt tttaaagttt tatttttaat aatttgcgag tacgca
//
```
